# Generating protein hydrogels with customizable stress relaxation behavior via deep learning-driven entanglement design

**DOI:** 10.1038/s41467-026-77607-9

**Published:** 2026-09-21

**Authors:** Puqing Deng, Yutong Wu, Hong Kiu Francis Fok, Linyan Li, Wen-Bin Zhang, Fei Sun, Hanyu Gao

**Affiliations:** 1 Department of Chemical and Biological Engineering, The Hong Kong University of Science and Technology, Clear Water Bay, Hong Kong; 2 Department of Data Science, City University of Hong Kong, Kowloon, Hong Kong; 3 Beijing National Laboratory for Molecular Sciences, Key Laboratory of Polymer Chemistry & Physics of Ministry of Education, Center for Soft Matter Science and Engineering, College of Chemistry and Molecular Engineering, Peking University, Beijing, P. R. China; 4 School of AI for Science (AI4S), Shenzhen Graduate School, Peking University, Shenzhen, P. R. China

**Keywords:** Protein design, Machine learning, Biomaterials - proteins

## Abstract

Protein hydrogels are promising artificial extracellular matrices (ECMs) for 3D stem cell and organoid culture due to their favorable stress relaxation behavior (a decrease in stress in response to strain). Inter-chain entangled motifs, in which different protein chains are interlaced, represent a powerful strategy to synthesize such hydrogels. However, designing these motifs with tailored properties such as binding energy remains a major challenge due to the difficulty of simultaneously controlling these properties while ensuring entanglement. Here, we introduce TangleDiff, a deep learning framework for the de novo design of homodimeric entangled proteins with programmable features. TangleDiff generates diverse foldable entangled sequences with an in-silico success rate exceeding 70%, markedly outperforming current models (~1%). By conditioning TangleDiff on inter-chain binding energy, we generate novel protein dimers whose binding energies closely match the specified ranges, with approximately 70% of successful designs conforming to expected values. We experimentally validate TangleDiff by designing nine homodimers targeting various binding energies; seven successfully form hydrogels, with stress relaxation dynamics correlated with specified binding energies. This work establishes a general strategy for entangled protein design, opening avenues for entanglement-based biomaterial innovation.

## Abstract (web summary)

Stress relaxation is increasingly recognized as a critical property of protein hydrogels for tissue engineering. To expand the limited toolbox of inter-chain entangled motifs, the authors develop an integrated workflow combining data mining, generative AI, virtual screening, and experimentation. The platform enables the design of entangled proteins and the fabrication of seven hydrogels with tailored stress relaxation profiles, demonstrating a direct correlation between predicted binding energies and material performance.

## Introduction

Protein hydrogels have been widely utilized as extracellular matrix (ECM) materials in tissue engineering, regenerative medicine and stem-cell culture, providing supportive scaffolds for incubated cells^1–5^. A key enabling factor is the viscoelasticity of protein hydrogels, which facilitates rapid mechanical energy dissipation through matrix remodeling and the rearrangement of cell-binding ligands whenever a traction force from cells is exerted. The viscoelasticity of an extracellular matrix, reflected in its stress relaxation behavior, significantly influences cellular processes including cell sensing, adhesion, spreading, proliferation, and differentiation^6–11^. Therefore, the stress relaxation rate has become a critical design parameter in tissue engineering^12,13^.

The introduction of inter-chain entangled protein domains into protein hydrogels is an effective approach to facilitate stress relaxation behaviors. The inter-chain entangled proteins are assemblies with chains physically interlaced. They provide noncovalent crosslinks in the networks, which enhance chain mobility and allow for faster force dissipation during mechanical remodeling^14^. However, the existing toolbox of inter-chain entangled motifs is limited to a few natural proteins, namely the p53 dimerization domain (p53dim)^15,16^, HP0242^17,18^, and their mutants^19^, resulting in restricted structural diversity and viscoelastic variability. De novo protein design opens a path to overcome this limitation and computationally specify and vary the structure and properties of proteins, with recent advances in generative deep learning algorithms demonstrating their powerful capability in creating diverse protein folds^20–23^. Still, the specific challenge of designing entangled topologies, such as knots (monomers with self-entangling backbones)^24,25^ or links (two chains interlinked through entanglements)^26,27^, from scratch remains particularly difficult, as their sterically entangled interfaces are kinetically unfavorable in protein folding^26,28^. Current efforts in entanglement designs heavily rely on the tandem duplications of naturally occurring repetitive units^29–31^, restricting achievable entangled sequences and structures to a limited subspace. Systematically varying sequential and structural characteristics of entangled dimers across an expansive design space and applying de novo designed entangled domains as physical crosslinks to viscoelastic protein networks remain central challenges in the field.

Here, we present an integrated framework that combines data mining, deep learning-based protein generation, virtual screening and experimentation to design entangled proteins and fabricate protein hydrogels with pre-defined stress relaxation behaviors. Our approach began with mining sequence databases to identify features conducive to chain intertwining (two chains entangled with each other). We then developed TangleDiff, a latent-diffusion-based generative model to generate diverse, foldable entangled sequences satisfying user-specified property constraints. These candidates were computationally screened for high designability before experimental selection. Finally, we experimentally expressed nine designs, eight of which demonstrated effective dimerization, and seven were successfully made into gels. The stress relaxation behaviors were directly correlated with pre-specified binding energies, demonstrating that manipulating the macroscopic properties of protein hydrogels can be achieved through the rational design of molecular-level interactions. This work establishes a general route to bespoke biomaterials, bridging molecular-level entanglement design with tunable functional properties.

## Results

### Entangled homodimer dataset construction

**Fig. 1:**
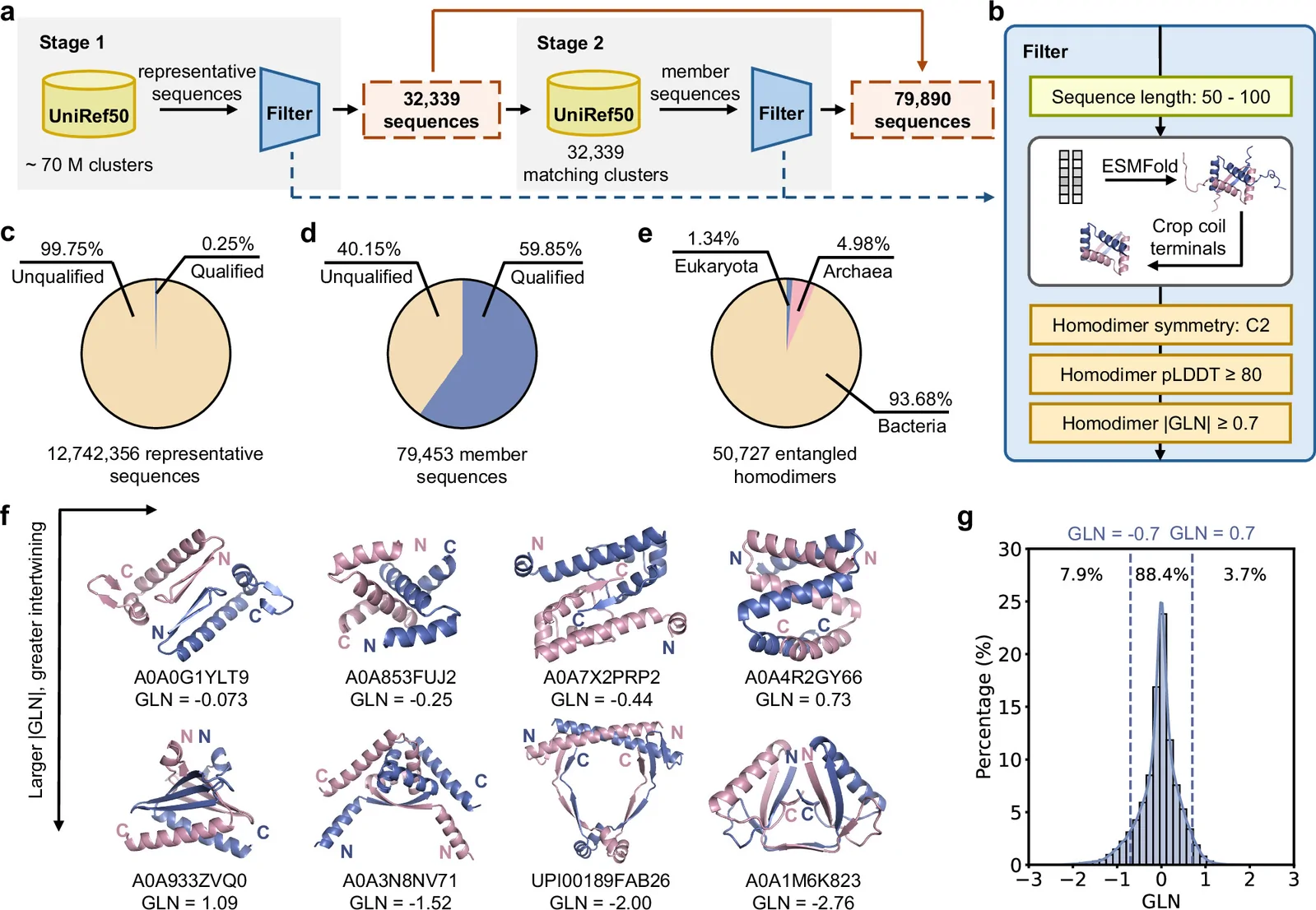
Dataset construction. **a** A two-stage data collection process from UniRef50. **b** Computational sequence filtering workflow applied to identify entangled homodimer structures with high quality and reliability. **c** Sequence screening hit rate of 12,742,356 representative sequences with a sequence length from 50 to 100 in UniRef50. **d** The sequence screening hit rate of 79,453 member sequences with a sequence length from 50 to 100 at the second stage. **e** 50,727 out of 79,890 discovered entangled sequences are successfully assigned into three domain groups. **f** Examples of entangled homodimers with varying |GLN| values; larger |GLN| indicates greater intertwining. Chains are distinguished by unique colors, with N-termini and C-termini labeled. **g** Distribution of GLN values for 100,000 randomly sampled homodimeric structures from the first screening stage, with percentage annotations for GLN ranges: < −0.7, −0.7–0.7, and > 0.7. Source data are provided as a Source Data file.

To identify the entanglement features encoded in protein sequences, we queried and processed sequences from UniRef50^32^, a database comprising approximately 70 million sequence clusters. We designed a two-stage screening strategy that balanced computational cost and traversed sequence diversity (Fig. 1a). In the first stage, we processed the representative sequence from each cluster to gather diverse entangling sequence information. In the second stage, we focused on member sequences from clusters whose representatives passed the first-stage screening. These member sequences typically share a higher sequence identity and are more likely to possess entangled homodimer structures, enabling the efficient identification of numerous sequences with a high hit rate (Fig. 1c, d).

In each stage, a computational filter (Fig. 1b) was employed to determine whether a sequence’s homodimer structure was entangled. We specified a sequence length of 50–100 amino acids. Sequences within this length range are sufficiently long to accommodate an entangled fold while remaining short enough to facilitate rapid computational processing at scale and swift structural folding in experiments. We predicted homodimeric structures using ESMFold^33^ and cropped their terminal coil regions to ensure that the subsequent entanglement detection step excluded structures where entanglement was formed by these terminal regions. The entanglements formed by the terminal coils are not robust due to their inherent flexibility (Supplementary Fig. 2). To collect entangled structures, we queried an absolute Gauss Linking Number (GLN) greater than or equal to 0.7^24,34–36^. GLN is a numerical invariant originally introduced to quantify the number of times that two closed curves wind around each other in three-dimensional space (Supplementary Fig. 3). For two open curves, such as protein chains, GLN is no longer invariant but still captures the entanglement extent, with a larger absolute GLN indicating a more severe entanglement^37,38^ (Fig. 1f). For example, dimers with a |GLN| of 0 have their subchains stacked together without any crossings, while dimers with a |GLN| of 2 exhibit subchains that wind around each other nearly twice. Compared to other entanglement detection techniques that rely on random chain closures, the GLN of two open chains is less computationally intensive and more suitable for large-scale analysis^27,39^. A quick molecular simulation of p53dim^40^, a typical entangled homodimeric domain, demonstrates GLN’s insensitivity to thermal fluctuations and small structural deviations (Supplementary Fig. 4), reinforcing its robustness as a detection criterion (see Methods and Materials for details). In this study, we established a |GLN| cutoff of 0.7 to differentiate between entangled and unentangled structures. Although this cutoff may vary depending on different definitions of entanglement or specific requirements for entanglement extent, previous results have shown that using 0.7 as a |GLN| threshold allows ESMFold to effectively identify entangled homodimers with high precision (> 0.8)^41^. Besides entanglement, we required C2 symmetry and an average predicted local distance difference test (pLDDT) score of 80 or higher, as provided by ESMFold. These two metrics were found to significantly improve both the reliability of the predicted homodimer structures and the entanglement detection precision in previous work^41^.

We used this screening strategy to process tens of millions of sequences and curated a large dataset comprising 79,890 sequences grouped into 32,339 clusters, using a sequence identity threshold of 50%. These entangled homodimers primarily belong to the bacterial domain (Fig. 1e) and encompass over 2951 cluster functions (Supplementary Fig. 5a) and 18,433 organisms (Supplementary Fig. 5b). This dataset provides diverse sequence information for both fundamental studies of entanglement and entanglement-related protein engineering, offering a foundation for investigating the taxonomic distribution and evolutionary significance of entangled homodimers—areas that remain poorly understood.

### Protein language diffusion model design and development

**Fig. 2:**
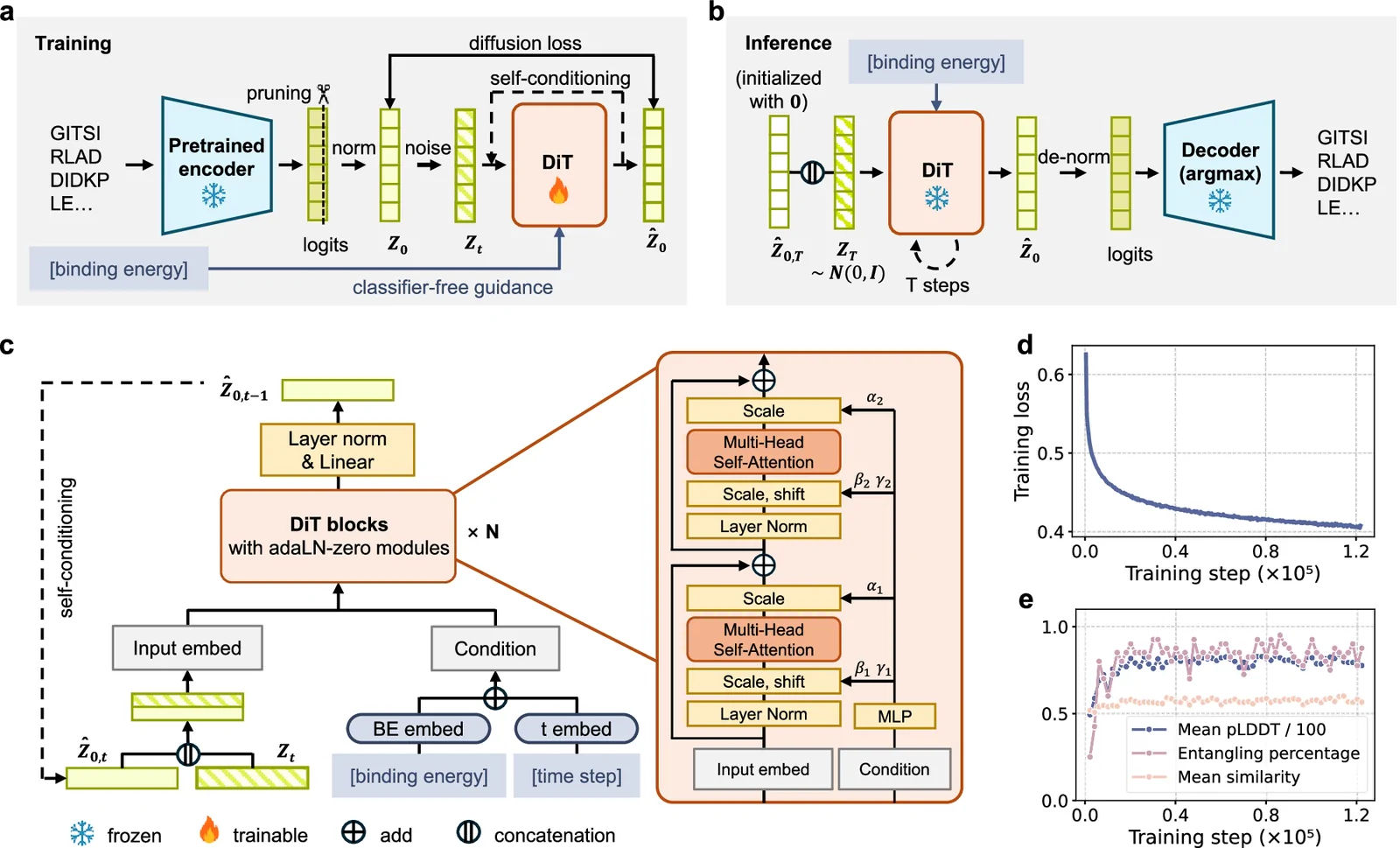
TangleDiff model design and development. **a** Latent diffusion training. Diffusion is applied to model the normalized logit distribution of a pretrained protein language model through learning to recover the noised latent. **b** TangleDiff inference. A randomly initialized Gaussian noise vector ${{{{\boldsymbol{Z}}}}}_{{{{\boldsymbol{T}}}}}$ undergoes a T-step prediction and denoising procedure, after which it is denormalized and decoded into the desired sequences. **c** DiT block architecture. At each diffusion step, the noised latent ${{{{\boldsymbol{Z}}}}}_{{{{\boldsymbol{t}}}}}$ and the un-noised latent predicted from the previous step ${\hat{{{{\boldsymbol{Z}}}}}}_{{{{\boldsymbol{0}}}},{{{\boldsymbol{t}}}}}$ are concatenated and fed into the Diffusion Transformer (DiT) architecture, which uses adaLN-zero DiT blocks to incorporate conditioning information and predicts the denoised latent ${\hat{{{{\boldsymbol{Z}}}}}}_{{{{\boldsymbol{0}}}},{{{\boldsymbol{t}}}}-{{{\boldsymbol{1}}}}}$. **d** The reconstruction loss (MSE) decreases as training progresses. **e** Metrics evaluated across 40 sequences sampled at different training steps indicate successful learning of the features of entangled protein sequences. Similarity refers to the closest sequence identity compared with training sequences. DiT Diffusion Transformer; adaLN-Zero adaptive layer normalization Zero; MSE mean squared error; BE binding energy. Source data are provided as a Source Data file.

Next, we developed a generative model, TangleDiff, to model entangling sequence distributions. We leveraged a pretrained protein language model (PLM) to translate tokenized protein sequences into continuous logit tensors and applied diffusion steps upon the logit tensors. PLMs were trained to extract evolutionary information from millions of sequences, enabling a comprehensive understanding of protein semantics^42,43^. To restrict the output vocabulary to the 20 standard amino acids, we retained only the 20 logit channels corresponding to the standard amino acid types. At each diffusion step, TangleDiff was trained to recover noised logit tensors that have been corrupted with Gaussian noise by minimizing a mean squared error (MSE) loss between the logit predictions and true logit tensors (Fig. 2a). This drove the denoising trajectories to converge on logit tensors that followed the distribution of natural entangled protein sequences. To sample a new protein sequence, we initialized a random Gaussian noise. At each sampling step, TangleDiff made an un-noised logit prediction with a neural network and updated the current noised logits to the next step in the direction of this prediction. As the model has been trained to align the denoising and noising process distribution, the initialized random noise will be mapped to meaningful logit tensors after enough diffusion steps and finally translated into generated protein sequences with an argmax decoder (Fig. 2b). We employed the Diffusion Transformer (DiT) architecture^44^, an architecture introduced for image generation, to make un-noised logit predictions at each step (Fig. 2c). Transformers effectively capture both local and global dependencies in sequential data while enabling parallel computation, making them suitable for extracting sequential features. Conditional generation was achieved by inputting conditions through an adaLN-Zero module^44^, which utilizes only a handful of parameters to bias the diffusion generation. A self-conditioning strategy^45^, which demonstrated great improvement in protein-generative performance^21^, was implemented by concatenating previously predicted logits with noised logits at each timestep (Fig. 2c and Supplementary Methods). The model was effectively trained and converged, as evidenced by the decreasing MSE (Fig. 2d) and improved generation performance (Fig. 2e) throughout the training process.

### Unconditional design of entangled homodimers

During training, we randomly dropped the condition c and replaced it with a learned null embedding (c=∅). This procedure enabled the model to learn an additional null-condition label and perform unconditional sampling when this label was specified, a procedure known as classifier-free guidance^46^. Through this method, TangleDiff combined the capabilities of both unconditional and conditional generation in a single trained model.

**Fig. 3:**
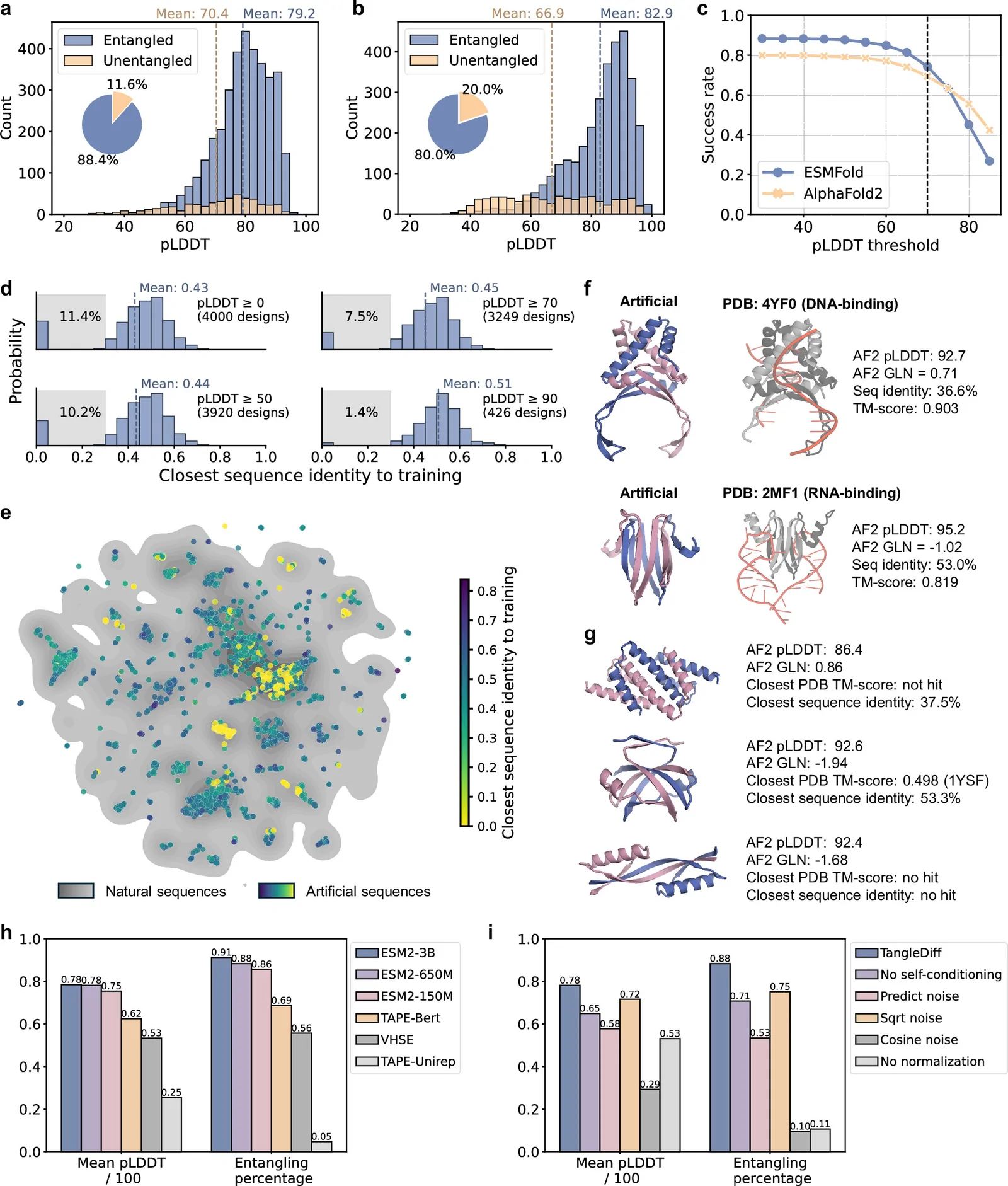
TangleDiff unconditionally generates novel and diverse protein sequences with high foldability and high tangling rate. **a** pLDDT distribution and entangling percentage of 4000 designed sequences as reported by ESMFold. **b** pLDDT distribution and entangling percentage of 4000 designed sequences as reported by AlphaFold2. **c** Success rate versus pLDDT threshold. The success rate is defined as the ratio of predicted structures that are both entangled and have a pLDDT above the specified threshold, calculated based on 4000 generated designs. **d** Distribution of the closest sequence identity of designed sequences to the training set across different pLDDT ranges. Closest sequence identity is reported by Blastp^64^ through a search against training sequences. The percentages of designs that have the closest sequence identities smaller than 0.3 (evolutionarily distant) are annotated. **e** Uniform Manifold Approximation and Projection (UMAP) of the protein-level ESM2-650M sequence embeddings of both natural and designed entangled homodimers. UMAP is fitted on 79,890 training sequences. Designed sequences are colored by their highest sequence identities compared to the training sequences. Natural entangling sequences are represented in gray, with deeper shades indicating higher density. **f** TangleDiff designs functional entangled homodimers that exhibit low sequence identity compared to known examples. Sequence identity and TM-score are reported according to the alignment part by Foldseek^65^. **g** TangleDiff designs novel entangled homodimers that do not resemble any structures in the known structure database. Closest sequence identity is reported by Blastp^64^ through a search against training sequences. **h** Comparison of different encoders used to map discrete protein sequences into continuous embeddings. Metrics are computed on 4000 unconditionally generated sequences for each encoder. **i** Ablation study. TangleDiff uses ESM2-650M as a sequence encoder and is trained to predict denoised latent with linear noise scheduling, normalization and self-conditioning techniques. Metrics are computed on 4000 unconditionally generated sequences for each setting. Source data are provided as a Source Data file.

To evaluate the unconditional generation performance of TangleDiff, we designed 4000 sequences with lengths sampled from an empirical distribution and used both ESMFold and AlphaFold2 to predict their homodimeric structures for comparison. We assessed the generation performance based on metrics including pLDDT and the percentage of entanglement derived from these predictions. TangleDiff can produce reasonable sequences that fold into reliable entangled homodimeric structures, with an average pLDDT of approximately 80 and an entangling percentage exceeding 80%, as reported by both ESMFold (Fig. 3a) and AlphaFold2 (Fig. 3b). The extent of entanglement in designed sequences shows a mild correlation with pLDDT, where sequences with low pLDDT values tend to be unentangled (Supplementary Fig. 6a). We considered a design to be successful if it was predicted to be entangled and had a pLDDT greater than 70, a threshold at which the structure prediction is deemed confident^33,47^. Consequently, TangleDiff demonstrates an overall in silico design success rate exceeding 70% (Fig. 3c), substantially surpassing that of current sequence generation models, with the best alternative achieving only 1.02% (Supplementary Fig. 8). The consistency of metrics derived from both ESMFold and AlphaFold2 predictions underscores TangleDiff’s robust generation performance, regardless of the structure predictor used. Additionally, TangleDiff designs novel entangled sequences. Most of the designed sequences exhibit closest sequence identities ranging from 0.4 to 0.6 compared to the training sequences, even among those with high pLDDT scores (Fig. 3d) and across different sequence lengths (Supplementary Fig. 6b). Some designs are evolutionarily distant (closest sequence identity < 30%), highlighting TangleDiff’s ability to generalize beyond natural entangled homodimers. The Uniform Manifold Approximation and Projection (UMAP) analysis of both training and generated sequences highlights the diversity of the designs, with 4000 designs effectively covering the sequence space defined by approximately 80,000 training sequences (Fig. 3e). The distribution of generated sequences closely aligns with that of the training data, demonstrating that TangleDiff successfully captures the characteristics of natural entangled sequences. Although TangleDiff defaults to 1000 denoising steps during sampling, a plausible sequence can be generated with significantly fewer steps and less time. Both foldability and entangling percentage remain high when the number of sampling steps is reduced to 500 and decrease only slightly when it is further reduced to 100 (Supplementary Fig. 6d), highlighting the rapid generation capabilities of TangleDiff.

The generated designs are also structurally diverse, spanning a wide range of both alpha-dominant and beta-dominant topologies (Fig. 3f, g). On one hand, TangleDiff can design functional entangled homodimers, such as DNA-binding and RNA-binding proteins (Fig. 3f), whose functionality can be inferred from closely related structures in the PDB, while exhibiting significant sequence novelty that surpasses what traditional mutagenesis methods can achieve. On the other hand, TangleDiff generates sequences that fold into novel structures without any resembling counterparts in the PDB, thereby expanding the landscape of dimeric entanglement (Fig. 3g).

To measure the contribution of the PLM to TangleDiff, we investigated different sequence encoders to obtain our diffusion latent. These included Evolutionary Scale Modeling (ESM2), a series of pretrained protein language models of different sizes; principal component score vectors of hydrophobic, steric, and electronic properties (VHSE-scales)^48^; and two representation learning models from the Tasks Assessing Protein Embedding (TAPE) series^49^: TAPE-Bert, which is transformer-based, and TAPE-Unirep^50^, which is based on recurrent neural networks. Our findings indicate that ESM2 models of all three sizes substantially outperformed the other encoders (Fig. 3h). Additionally, the performance of ESM2 improved slightly with increasing model size. Importantly, since we froze the parameters in the encoder during training, this performance enhancement is not due to model overfitting, but rather to a more effective latent embedding learned from a larger pretrained language model. These results highlight the importance of training diffusion on a meaningful sequence latent for TangleDiff’s performance. We also conducted an ablation study to evaluate various strategies employed during training (Fig. 3i). Results revealed that the prediction of the initial latent instead of the noise at each timestep, the self-conditioning applied, the linear noise scheduling, and the normalization of ESM2 logits all significantly contributed to the strong performance of TangleDiff.

### Conditional design of entangled homodimers with different binding energies

**Fig. 4:**
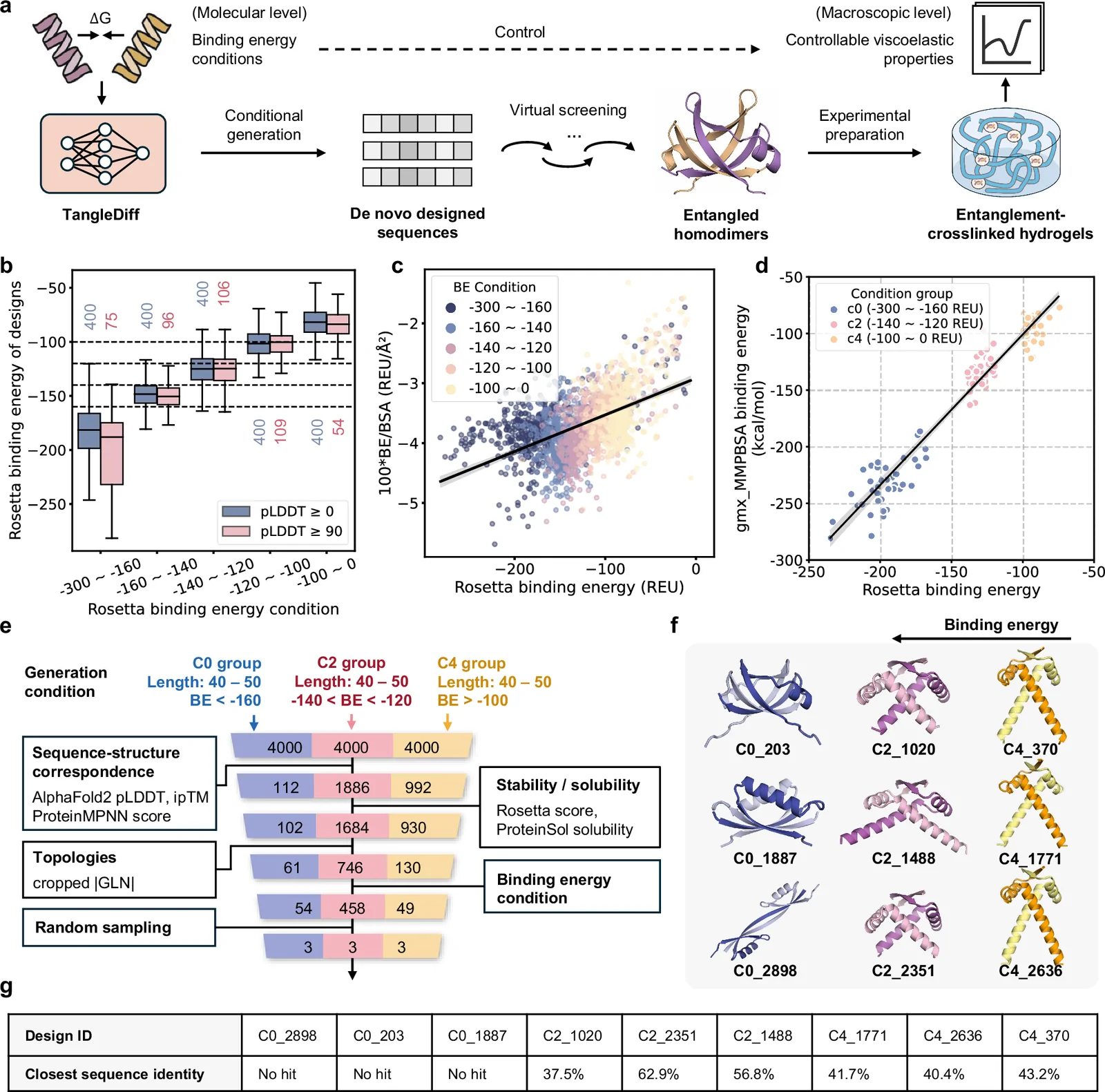
Design of entangled homodimers with different inter-chain binding energies and virtual screening. **a** Schematic of controlling rheological behaviors of entanglement-crosslinked hydrogels by conditioning the generation of TangleDiff on binding energies. **b** The inter-chain binding energies of the designed entangled homodimers closely match the specified binding energy range used during their generation. 400 sequences were generated under each condition. The numbers refer to the number of designs represented by each bin. The central line indicates the median, the box bounds indicate the 25th and 75th percentiles, and the whiskers indicate the minimum and maximum values within 1.5 times the interquartile range. Outliers beyond the whiskers are not shown. **c** Correlation between Rosetta binding energy and Rosetta binding energy per binding surface area. BE Condition refers to the specified condition during generation. 100*BE/BSA refers to 100 times Rosetta binding energy over buried surface area. 400 sequences were generated under each condition. **d** Correlation between the binding energies calculated by Rosetta and gmx_MMPBSA. Different colors indicate different Rosetta binding energy ranges. **c**, **d** The solid black lines indicate linear fits, and the shaded bands indicate 95% confidence intervals estimated using 1000 bootstrap resamples. **e** Virtual screening process of 4000 generated sequences under each binding energy condition. BE refers to Rosetta binding energy. Numbers in black refer to the number of sequences initially generated or kept after each screening step. Sequences generated by different binding energy conditions are marked by different colors. **f** The AlphaFold2-predicted structures of nine designed entangled homodimers selected for experimental validation and hydrogel preparation. **g** Closest sequence identities to the training sequences of nine selected designs. Source data are provided as a Source Data file.

TangleDiff can be trained and conditioned on any protein-level labels with its adaLN-Zero modules. To demonstrate this capability, we trained TangleDiff to generate entangled proteins with specific inter-chain binding energies, which are important molecular-level properties for modulating the stress-relaxation behaviors of entanglement-crosslinked protein hydrogels, a crucial design parameter for tissue engineering biomaterials^51,52^. We hypothesized that protein hydrogels crosslinked by entangled domains with varying binding energies could lead to different crosslinking strengths, resulting in distinct stress-relaxation rates (Fig. 4a).

We started by calculating binding energies between protein chains in our training set with Rosetta^53^. Rosetta defines its energy term by weighing a series of measurable geometric statistics or classical physics interaction energies with the unit of this energy term as Rosetta Energy Unit (REU). Rosetta calculates the binding energy by subtracting the energy of two proteins when pulled apart from the energy of the complex. Although this score does not provide absolute binding energies, it still measures a relative magnitude between different protein dimers and runs much faster compared with other molecular-simulation-based methods^54^, making it suitable for an initial generation conditioning and large-scale computing. Consequently, almost all homodimers in the training set were calculated with a Rosetta binding energy range from -300 to 0 REU, with values around -150 REU being the most prevalent (Supplementary Fig. 9).

During training, protein sequences were assigned to five binding-energy bins. During the generation process, starting from random noise, we specified a binding energy bin to guide the generation towards proteins belonging to the corresponding binding energy class. The binding energies of the generated proteins could be effectively controlled by these condition labels. For all five condition ranges, most of the generated proteins exhibited binding energies that fell within the specified bins (Fig. 4b). Although not explicitly specified during generation, the binding energy per area of generated designs exhibited a trend consistent with the overall binding energy (Fig. 4c). Furthermore, the binding energy range of these designs can even extend beyond that of the natural sequences mined from UniRef (Supplementary Fig. 11), demonstrating TangleDiff’s strong generalizability beyond nature. The binding energy differences between different designs can be further validated by gmx_MMPBSA^55^, a tool performing End-State free energy calculations based on molecular simulation trajectories (Fig. 4d).

We utilized TangleDiff to generate sequences conditioned on three specific binding energy groups: less than −160 REU (named C0), greater than −100 REU (named C4) and between −140 and −120 REU (named C2); 4000 sequences for each condition. All sequences were sampled with short sequence lengths from 40 to 50 amino acids, as shorter sequences are more kinetically favored to fold into entangled structures. To facilitate experimental validation, we designed a virtual screening process to filter the generated sequences (Fig. 4e). This process was based on criteria ensuring that (1) the designed sequences correspond to the predicted structures (sequence-structure correspondence), (2) the structures are entangled and stably folded, and (3) the proteins can be expressed in a soluble form (see Methods for details). The distribution of different metrics employed and the number of designs retained after each screening step in each group are presented in Supplementary Fig. 12 and Supplementary Table 1, respectively. Ultimately, we identified 54, 458, and 49 proteins for the different binding energy conditions. To ensure a manageable set for experimental validation while maintaining representativeness, three entangled designs from each binding energy range were randomly selected for further experimental validation and protein hydrogel preparation (Fig. 4f, g and Supplementary Fig. 1). Clustering analyses demonstrated that these designs are diversely distributed in the design space (Supplementary Figs. 13 and 14). Interestingly, designs with distinct binding energies exhibited different entanglement structure patterns in their AlphaFold2-predicted structures. In designs with strong binding energies (< −160 REU), entanglements are primarily predicted to be formed by beta sheets. In contrast, designs with intermediate (−140–−120 REU) and weak (> −100 REU) binding energies displayed entanglements associated with the ribbon-helix-helix (RHH) superfamily, with the weaker ones showing shallower entangled structures. All designs with strong binding energies (< −160 REU) lack similar sequence matches in the training set, demonstrating the highest novelty among the three groups. This observation may be attributed to the greater structural diversity and variability of beta sheets compared to alpha helices^56^.

### Experimental validation and preparation of entanglement-crosslinked protein hydrogels

**Fig. 5:**
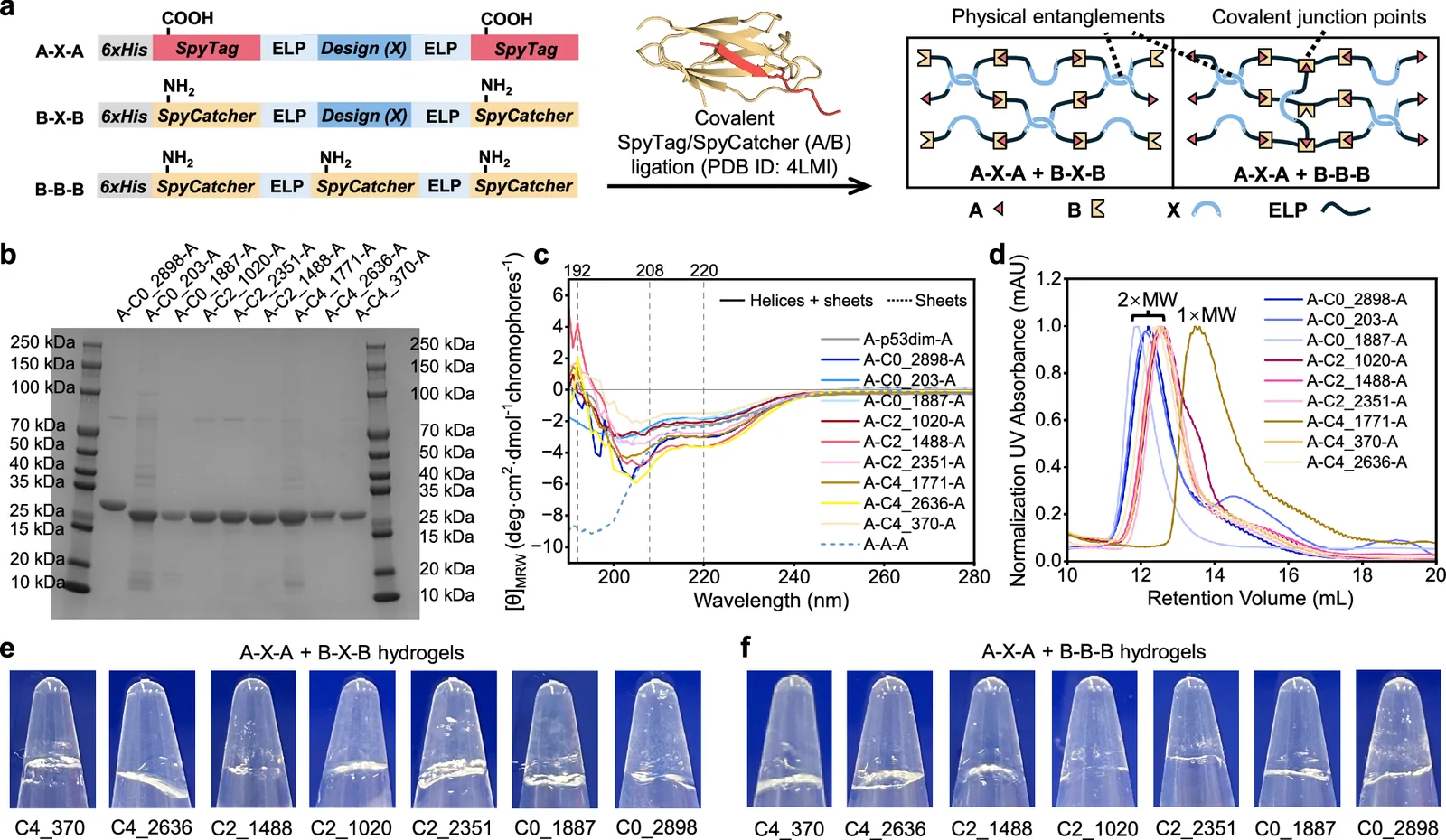
Experimental validation and preparation of entanglement-crosslinked protein hydrogels. **a** Constructs of A-X-A, B-X-B and B-B-B and the resulting networks formed through SpyTag/SpyCatcher covalent isopeptide ligation. A-X-A + B-X-B forms linear covalent chains whose gel-like properties arise from physical entanglement of the designed X domains. A-X-A + B-B-B forms a covalently crosslinked branched network in which both covalent junctions and physical entanglements contribute to the mechanical properties. **b** SDS-PAGE of nine A-X-A designs. The experiment was independently repeated 3 times with similar results. **c** CD spectroscopy was performed on nine A-X-A designs, as well as on A-A-A and p53dim as controls. **d** SEC-MALS results of nine A-X-A designs. The measurements were independently repeated 3 times with similar results. **e** Seven prepared protein hydrogels of A-X-A + B-X-B type. **f** Seven prepared protein hydrogels of A-X-A + B-B-B type. Source data are provided as a Source Data file.

To experimentally validate our entangled designs with different binding energies and create corresponding protein hydrogel networks, we developed three types of genetic constructs: SpyTag-ELP-design-ELP-SpyTag (A-X-A), SpyCatcher-ELP-design-ELP-SpyCatcher (B-X-B), and SpyCatcher-ELP-SpyCatcher-ELP-SpyCatcher (B-B-B). These constructs are similar to those used in previous studies^14^, but with the X domain replaced by our nine designed entangled domains, resulting in a total of 19 distinct gene constructs. The ELP domains, made up of repetitive pentapeptides (VPGXG)_15_, where X can be either Val or Glu at a 4:1 ratio, serve as connection spacers within the constructs^57^. By utilizing these constructs as building blocks and the SpyTag/SpyCatcher system as ligation tools, we synthesized two types of protein networks: A-X-A + B-B-B and A-X-A + B-X-B. Both A-X-A + B-X-B and A-X-A + B-B-B networks are formed through SpyTag/SpyCatcher covalent isopeptide ligation. However, the A-X-A + B-X-B network has a building block functionality of 2, resulting in linear covalent chains whose gel-like properties arise from physical entanglement of the designed X domains. In contrast, the A-X-A + B-B-B network has a functionality of 3, forming a covalently crosslinked branched network in which both network junction points and physical entanglements contribute to mechanical properties (Fig. 5a).

All fused proteins were successfully expressed in a soluble form in *Escherichia coli* (Fig. 5b and Supplementary Fig. 16), demonstrating the effectiveness of TangleDiff and the designed virtual screening workflow in producing soluble and properly folded proteins. The secondary structures of all nine designs were verified using circular dichroism (CD) spectroscopy, revealing an alpha-beta mixing pattern similar to that of p53 dimers, but distinct from that of A-A-A proteins, which contain only random coils and a small portion of beta sheets (Fig. 5c). The dimeric states of eight out of the nine designs were confirmed by size-exclusion chromatography with multi-angle light scattering (SEC-MALS), with the MALS detector indicating molecular weights approximately double that of the monomer (Fig. 5d, Supplementary Fig. 17 and Supplementary Table 3). The design C4_1771, from the weakest binding energy group (C4 group), was observed in a monomeric form in solution, which aligns with the computational results that the C4_1771 homodimer exhibited a large structural deviation during 200 ns of molecular simulation (Supplementary Fig. 15).

**Fig. 6:**
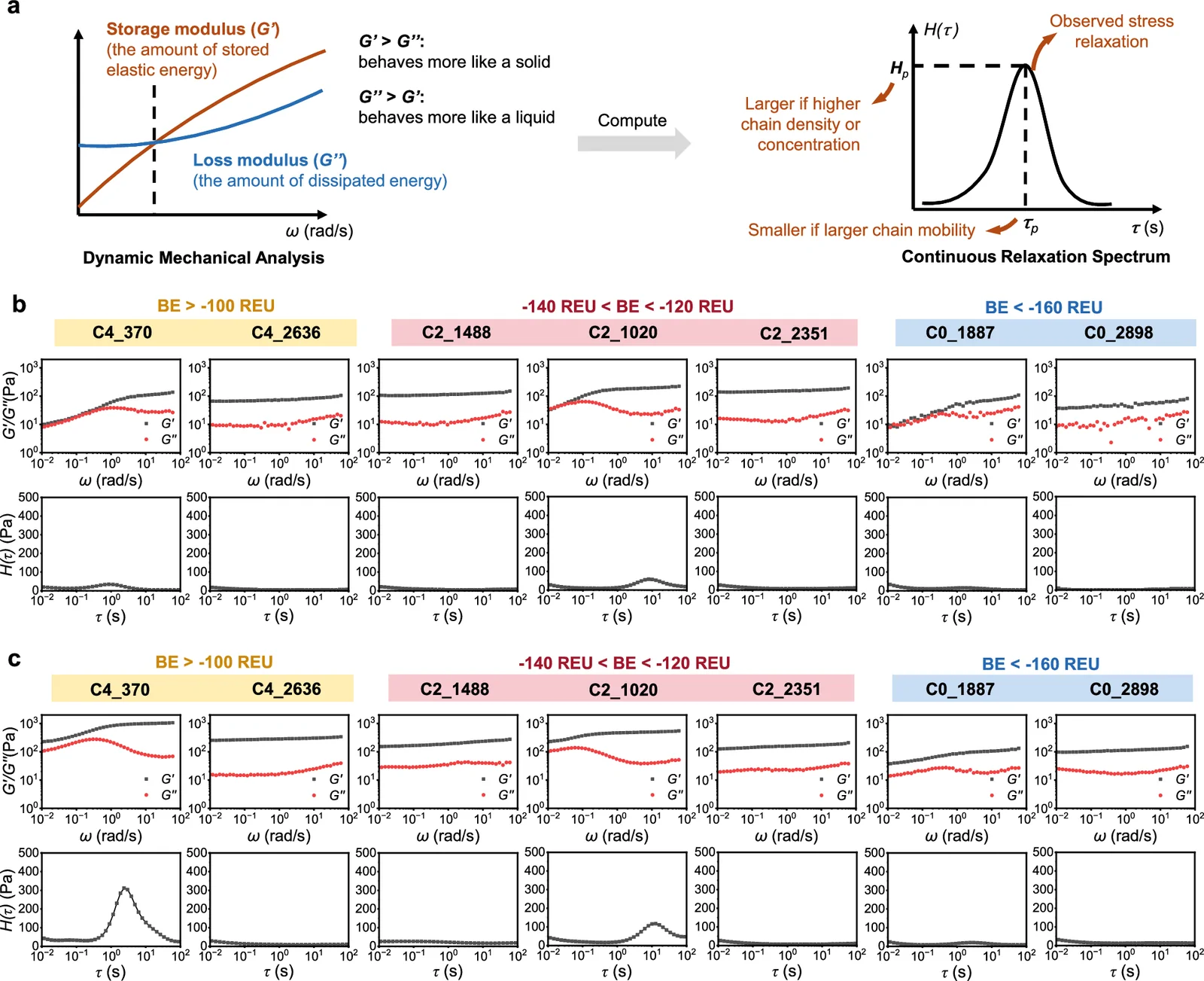
Rheological tests of 14 prepared protein hydrogel networks. **a** Schematic of dynamic mechanical analysis and continuous relaxation spectrum. **b** Dynamic mechanical analysis and computed continuous relaxation spectra of the A-X-A + B-X-B networks. **c** Dynamic mechanical analysis and continuous relaxation spectra of A-X-A + B-B-B networks. For both **b** and **c**, BE refers to the computed Rosetta binding energy. The continuous relaxation spectra H(τ) were calculated based on their mathematical relation to G′(ω) and G″(ω) (Methods). Source data are provided as a Source Data file.

By mixing the precursor solutions followed by the ligation reaction between the A and B, seven out of eight designs were successfully made into both types of gel-like material: A-X-A + B-X-B (Fig. 5e) and A-X-A + B-B-B (Fig. 5f). Although the A-X-A + B-B-B networks demonstrate greater applicability for cell incubation due to their higher mechanical strength, we also prepared the A-X-A + B-X-B networks, whose gel-like properties only arise from physical entanglements, to highlight the contribution of chain entanglements. Dynamic mechanical analysis was performed to assess the rheological behaviors of the prepared networks by measuring the storage (${G}^{{\prime} }$) and loss modulus (${G}^{{\prime} {\prime} }$). This assessment indicates whether the materials exhibit predominantly solid-like or fluid-like behavior (Fig. 6a). To quantify the full distribution of relaxation times and correlate the macroscopic stress-relaxing behavior with binding energies at the molecular level, we converted the frequency sweep data (${G}^{{\prime} }$ and ${G}^{{\prime} {\prime} }$) into a series of continuous relaxation spectra H(τ) (Methods). Peaks in the continuous relaxation spectra reflect the relaxation behavior, with their positions (relaxation times) determined by the chain mobility (the ability of chains or chain segments to undergo conformational rearrangements, translation, or reorientation over time), which we hypothesize is dominantly influenced by inter-chain binding energy (Fig. 6a).

As a result, A-X-A + B-X-B hydrogels made from C4_370 (C4 group, binding energy > −100 REU) and C2_1020 (C2 group, binding energy between −140 and −120 REU) exhibited a significant local maximum of loss modulus ${G}^{{\prime} {\prime} }$ and crossovers of storage modulus ${G}^{{\prime} }$ and loss modulus ${G}^{{\prime} {\prime} }$ were observed at low frequencies, indicative of the formation of viscoelastic liquids. In contrast, A-X-A + B-X-B hydrogels made from designs with the strongest binding energy (C0 group, binding energy < −160 REU) demonstrated elastic properties with no significant stress-relaxation behavior (Fig. 6b). Similarly, frequency sweep curves of A-X-A + B-B-B networks exhibited the same trend as A-X-A + B-X-B, except that due to the introduction of covalent crosslinks, the ${G}^{{\prime} }$ was much larger and no crossover of ${G}^{{\prime} }$ and ${G}^{{\prime} {\prime} }$ was observed, indicating the formation of viscoelastic solids (Fig. 6c). The relaxation spectra of the A-X-A + B-X-B networks formed by C4_370 and C2_1020 revealed a clear dissipation peak at ω ~ 0.3 rad/s (τ ~ 2.4 s) and 0.06 rad/s (τ ~ 12.8 s), respectively, indicating their rapid stress-relaxation behaviors due to the inclusion of weakly-bound physical entanglement crosslinks. The appearance of the dissipation peak for C4_370 at a higher frequency than that for C2_1020 confirmed its weaker binding energy relative to C2_1020. The absence of relaxation peaks in the C0 group reflects the strongest binding energy. In contrast, the A-X-A + B-B-B networks exhibited relaxation peaks at the same positions but with higher peak heights, demonstrating that the introduction of covalent junction points increases the relaxation strength without affecting the relaxation time or introducing new relaxation behaviors. Additionally, relaxation peaks were not observed in hydrogels prepared from designs C4_2636, C2_1488, and C2_2351. We propose that these unexpected findings may be attributed to various factors, such as differing structural features (e.g., buried surface area, secondary structure content, terminal distances) or additional intermolecular interactions.

As a negative control, we prepared A-X-A + B-X-B and A-X-A + B-B-B hydrogels from the design C4_1771, which has a similar length to the other designs but does not exhibit dimerization or entanglement (as indicated by the SEC-MALS results). Unlike the other designs that formed stable gels, both network types prepared from C4_1771 remained viscous liquids that gradually flowed down the walls of the container (Supplementary Fig. 18). Their ${G}^{{\prime} }$ was significantly lower than that of the other designs, and the relaxation spectra also showed no evidence of stress relaxation behavior. This proves that extra physical entanglements were necessary for the gel formation and that the relaxation behavior observed in the hydrogels originated from physical entanglements within the networks, rather than from other compositions such as covalent junction points or ELP chain dynamics.

To experimentally confirm the structural entanglement, we selected C4_370, which exhibited the most pronounced relaxation behaviors in the prepared hydrogels, as a representative design. We designed the constructs (B-C4_370-A) using the gene cassette previously applied to catenane synthesis^58,59^. Following purification of the dimeric products, we conducted tobacco etch virus (TEV) protease-mediated cleavage (Supplementary Method). The production of cyclic monomers during the digestion process provides evidence for the existence of catenanes in the dimeric products, as well as for the structural entanglement present in the designs prior to the formation of covalent linkages (Supplementary Fig. 19).

Collectively, these experimental results highlight TangleDiff’s capability in designing entangled proteins for the preparation of stress-relaxation hydrogels and in controlling their stress-relaxation rates through biased generation.

## Discussion

Compared to previous work that designs knots and domain-swapping intertwined dimers through tandem duplications of repetitive protein units^29–31^, our data-driven design pipeline offers several advantages: the design process requires minimal expertise, spans a much larger structural and sequence design space, and enables controls according to user-defined molecular properties. In contrast to entangled protein mining from natural sources^41,60^, our de novo design approach generates novel protein sequences and structures that may not exist in nature while enabling tailored designs that meet specific criteria. TangleDiff implicitly learns entanglement features encoded in our curated sequence dataset and directly generates complete entangled domains, circumventing the need for domain expertise and precise geometric control over repetitive units. The designed intertwined dimers demonstrated significant sequence and structural diversity, with their binding energies closely matching the specified ranges. In our model architecture, the binding energy condition can be replaced by other molecular-level properties, such as entanglement extent and secondary structure ratios, allowing for various types of manipulation. Moreover, entangled proteins serve as significant precursors to topological proteins beyond hydrogels, broadening the potential impact of TangleDiff on biomaterial design.

Integration of entanglement crosslinks into protein networks largely facilitates their stress relaxation properties^14^. However, the limited availability of entangled protein domains and the substantial challenges associated with the de novo design of sterically entangled interfaces raise concerns regarding the structural diversity and tunability of stress relaxations within these networks. Our method advances the field of entanglement-crosslinked protein hydrogels by facilitating the efficient design of foldable, novel, and diverse entangled proteins with controllable binding energy through TangleDiff, a transformer-based diffusion model integrated with the adaLN-Zero module. By utilizing this model to conditionally generate entangled designs with pre-specified inter-chain binding energies and incorporating these designs into protein networks, our approach facilitates the preparation of protein hydrogels with tunable stress relaxation rates. As entangled proteins continue to advance biomaterial design, our method can significantly enhance the design space and tunability of entangled protein domains. Our data-driven method integrates data mining, machine learning, virtual screening and experimentation, providing an end-to-end strategy for controlling macroscopic gel properties and opening avenues for ECM design in 3D stem cell and organoid culture.

Although TangleDiff generates some novel sequences that are homologously or structurally distant from the training data (with closest sequence identities < 30% or closest TM-scores < 0.5), the proportion of such sequences is limited, which makes TangleDiff more suited for sequence-sensitive property design. This limitation could be mitigated by either increasing the diversity of training sequences through a broader-scale search or training the model with a lower clustering threshold (e.g., 30%).

It is also important to note that while this study trained TangleDiff with a focus on inter-chain binding energy, the conditioning framework can be extended to other customized molecular-level properties, such as the extent of entanglement, kinetic stability, secondary structure composition, which affects stiffness and terminal distance, a crucial parameter in topological engineering^37^. The diffusion framework of TangleDiff also enables seamless integration of a property discriminator, facilitating customized conditional generation based on various criteria without the need for additional training^61^. As TangleDiff is a sequence generation model, it can easily be extended to generate entangled homotrimers with C3 symmetry or even higher-order structures by training with appropriate data. This work focuses solely on entangled homodimers because the accuracy of ESMFold in identifying entangled homotrimers or higher-order assemblies is currently limited, which also constrains the performance of our computational screening pipeline. Therefore, there is a need for the development of high-throughput algorithms to discover complex entangling motifs. We believe that entangled proteins with higher-order symmetries, such as C3 or D2, would significantly enhance the complexity and diversity of entanglement-crosslinked protein networks, making this an attractive direction for future work.

## Methods

### Dataset construction

We queried all representative sequences from UniRef50 with a length ranging from 50 a.a. to 100 a.a. (12,742,356 sequences released on and before 2 December 2024) and used ESMFold (v2023_02) to predict their homodimer structures with two identical sequences linked by a soft linker (25 consecutive glycine residues). MDTraj^62^ was used to assign 3-category secondary structures, based on which the terminal coil regions were cropped. Symmetries were determined with an acceptable root mean square deviation (RMSD) of 3 Å by AnAnaS^63^.

After ESMFold homodimeric structure prediction, we cropped the random coil terminals for two reasons: ESMFold tends to report low pLDDT scores in terminal disordered regions, which reduces the overall mean pLDDT across the sequence. By removing these regions, we increase the amount of data that meets the specified pLDDT threshold. Second, the coil terminals of the two chains in ESMFold predictions can be sufficiently long to intertwine, resulting in entanglements that, despite having high pLDDT scores, are formed through coil structures that are not robust due to their highly flexible nature.

Entanglement was identified by GLN. In contrast to other entanglement detection methods that necessitate random chain closures, GLN for open chains is less computationally intensive and more suitable for large-scale analysis. Generally, two protein chains with a more severe entanglement will result in a larger absolute GLN value. The GLN for two open backbones can be derived by:

1 $${{{\rm{GLN}}}}={\sum }_{i=1}^{{N}_{A}-1}{\sum }_{j=1}^{{N}_{B}-1}\frac{({{{{\boldsymbol{r}}}}}_{{{{\boldsymbol{i}}}}}^{{{{\boldsymbol{A}}}}}\times {{{{\boldsymbol{r}}}}}_{{{{\boldsymbol{j}}}}}^{{{{\boldsymbol{B}}}}})\cdot {{{{\boldsymbol{l}}}}}_{{{{\boldsymbol{ij}}}}}}{4\pi {\parallel {{{{\boldsymbol{l}}}}}_{{{{\boldsymbol{ij}}}}}\parallel }^{3}}$$

where ${{{{\boldsymbol{r}}}}}_{i}^{A}$ is the vector between the i^th^ and the (i+1)^th^ α-carbon in chain A, pointing from N-terminus to C-terminus and ${{{{\boldsymbol{r}}}}}_{j}^{B}$ is the vector between the j^th^ and the (j+1)^th^ α-carbon in chain B. ${{{{\rm{GLN}}}}}_{{ij}}$ is the GLN between any two vectors ${{{{\boldsymbol{r}}}}}_{i}^{A}$ and ${{{{\boldsymbol{r}}}}}_{j}^{B}$ from different protein chains. ${N}_{A}$ and ${N}_{B}$ are the number of residues in chain A and B, respectively. ${{{{\boldsymbol{l}}}}}_{{ij}}$ is the vector between the middle point of ${{{{\boldsymbol{r}}}}}_{i}^{A}$ and ${{{{\boldsymbol{r}}}}}_{j}^{B}$.

We only kept cropped structures with a C2 symmetry, pLDDT ≥ 80 and |GLN| ≥ 0.7, yielding 32,339 representative sequences. These representative sequences correspond to 32,339 sequence clusters. To collect more data, we further queried 79,453 extra sequences with their sequence identity smaller than 90% and a sequence length smaller than 100 from these clusters and applied the same filtration steps previously discussed, leading to 47,551 extra qualified sequences. Our final curated dataset consists of 79,890 entangled proteins whose sequences can be grouped into 32,339 clusters at a sequence identity threshold of 50%.

### Model training

The model was trained to minimize the MSE loss between the predicted un-noised logits and the true logits. In each epoch, we randomly sampled one sequence from each cluster and padded it to the maximum length in each batch. Masks were applied in the self-attention layers, with 1 indicating residue positions and 0 indicating padding positions. Parameters were updated with an Adam optimizer (β_1_: 0.9, β_2_: 0.999) and a learning rate of 1 × 10^−4^. We trained our model on four GeForce RTX3090 GPUs with a global batch size of 128. To monitor the training process, we sampled 40 sequences every 2000 steps and measured their average ESMFold-predicted homodimer pLDDT, entangling percentage of homodimers and their closest sequence identities compared to the training sequences. We ended our training when all these metrics converged (118,000 steps, about 20 h).

### Sampling and evaluation

TangleDiff sampled sequences in batches, with mask tokens padded at the ends to define the lengths of each sample within the batch. Users can specify the lengths, or, if not provided, lengths will be randomly sampled from the empirical distribution of training sequences. The sequences were generated through 1000 denoising steps by default.

We used a threshold of |GLN| ≥ 0.7 to identify chain entanglement in generated samples. Blastp^64^ was used to search training sequences for the closest sequence identity of designs with an E-value of 0.00001 and the number of alignments set to one. Foldseek^65^ was used to report the closest TM-score in training sequences with the ‘easy-complexsearch’ command and a minimum fraction of aligned residues set to 0.7.

### Rosetta binding energy calculation

We relaxed all homodimer structures predicted by ESMFold in the training set using Rosetta before binding energy calculation. To calculate the Rosetta binding energy from the relaxed structures, we employed the ‘InterfaceAnalyzer’ protocol with the ‘-pack_input’ and ‘-pack_separate’ options. The ‘-pack_input’ option pre-packed the structures prior to separating the chains, while the ‘-pack_separate’ option repacked the exposed interfaces during the binding energy calculation.

### Virtual screening

Starting from random Gaussian noise, we generated 4000 sequences with lengths randomly sampled between 40 and 50 amino acids, conditioned on three binding energy ranges: less than −160 REU, between −140 and −120 REU, and greater than −100 REU. AlphaFold2 was employed to predict the homodimer structures of the generated sequences. To enhance the reliability of these predictions, we selected structures with an ipTM score greater than 0.8 and a pLDDT score exceeding 90. Subsequently, we utilized ProteinMPNN^66^ to score the predicted structures and sequences, retaining designs with scores below 1.4906, which is the score of p53dim^67^, a widely recognized entangled domain. We then assessed the stability and solubility of the designs using Rosetta and ProteinSol^68^. Designs with a single-chain Rosetta score of less than −65 (as determined by p53dim), a complex Rosetta score of less than −200 (as determined by p53dim), and a ProteinSol-predicted solubility greater than 0.45, which is the average solubility of proteins in *E. coli*, were subjected to further filtration. Finally, we required designs to exhibit entanglements (|GLN| greater than or equal to 0.7) and binding energies within the specified ranges. Three designs from each binding energy range were randomly picked for experimental validation.

### Molecular dynamics

Molecular dynamics (MD) simulations of nine protein homodimer structures selected for experimental validation were performed for 200 ns using GROMACS (2024.3)^69^ with the Amber99SB force field to evaluate the stability of the protein homodimer structures. The Amber99SB force field was chosen for its proven accuracy in simulating protein folding and dynamics, and the SPC/E water model was selected for its compatibility with Amber force fields. All simulations were performed in explicit solvent to accurately capture protein-water interactions and conformational dynamics. We defined a cubic simulation box filled with water. The protein was centered in the box, and the dimensions of the box were set to ensure a minimum distance of 1 nm between the protein and the box edge. A specific number of water molecules was replaced with either Na⁺ or Cl^−^ ions to neutralize the charge of the simulated protein. Prior to the MD run, the energy of the system was minimized to ensure that there were no steric clashes or inappropriate geometries. The systems were gently annealed at 300 K under the NVT ensemble (constant Number of particles, Volume, and Temperature) for 100 ps, followed by the NPT ensemble (constant Number of particles, Volume, and Pressure) for an additional 100 ps to achieve equilibrium. Following the 100-ps NVT and 100-ps NPT equilibration, a 200-ns production run was performed for each system. Data from the production run were analyzed for subsequent stability assessments. The RMSD relative to the structure present in the minimized and equilibrated system was calculated at each timestep to assess the stability of the protein homodimers. Equilibration was assessed by monitoring the root-mean-square deviation (RMSD) of the protein backbone and the system potential energy as a function of simulation time. The systems were considered equilibrated when both RMSD and potential energy plateaued (Supplementary Fig. 15). Due to the extensive 200-ns simulation time and the observation of stable RMSD plateaus, the results are considered independent of the initial configuration.

### Gene construction, protein expression and purification

Custom plasmids harboring genes encoding the A-X-A, B-X-B or B-B-B proteins were obtained from Genewiz. The plasmids were transformed into *E. coli* strain BL21(DE3) and cultured in LB medium at 37 °C with shaking at 220 rpm until reaching an optical density (OD_600_) of 0.5–0.6. Protein expression was induced with 320 μM isopropyl β-D-1-thiogalactopyranoside (IPTG) at 37 °C for 3 h. Cells were then harvested by centrifugation at 5150 × *g* and 4 °C for 20 min. Cell pellets were resuspended in lysis buffer (300 mM NaCl, 50 mM Tris-HCl, pH 8.0) and were stored at −80 °C overnight prior to protein purification.

The frozen cell pellets were thawed, resuspended in lysis buffer supplemented with 1 mM phenylmethylsulfonyl fluoride (PMSF), and lysed by sonication. The proteins were purified using HisTrap HP Ni-NTA columns (Cytiva). SDS-PAGE was used to assess protein purity. The purified proteins were desalted using a HiTrap Desalting column (Cytiva), snap-frozen in liquid nitrogen, lyophilized at −80 °C for 5 days and stored at −80 °C until use.

### Secondary structure and oligomerization state measurement

CD spectroscopy was conducted with a JASCO J-815 spectropolarimeter to confirm the secondary structures of designed proteins. The spectra were recorded in the wavelength range of 190–280 nm at a scanning rate of 50 nm/min. Measurements were performed at room temperature using a 1 mm path length quartz cuvette. Protein samples were prepared at a concentration of 20 μM in PBS. Curves were averaged over 3 scans to improve the signal-to-noise ratio. Background subtraction was performed using a buffer as the blank. The SEC-MALS system from Wyatt Technology, equipped with a Superose 12 10/300 GL column, was utilized to test the oligomerization state of purified proteins.

### Hydrogel preparation and dynamic shear rheology

Lyophilized protein powders were dissolved in phosphate-buffered saline (PBS) buffer (pH 7.4) to prepare 8 wt% protein solutions. The A-X-A + B-B-B network was produced by mixing A-X-A solutions and B-B-B solutions with a molar ratio of 3:2, whereas the A-X-A + B-X-B network was prepared by mixing two solutions with a molar ratio of 1:1. For both A-X-A + B-B-B and A-X-A + B-X-B, the total volume of each mixed solution was 50 µL. Rheological measurements were performed on a TA Instruments ARES-G2 strain-controlled rheometer with a standard steel parallel-plate geometry (8-mm diameter). Test modes included dynamic time sweep, frequency sweep, and strain sweep. Hydrogels of both A-X-A + B-B-B and A-X-A + B-X-B networks were monitored at 25 °C via time-sweep measurements while the strain was held constant at 5% and the test frequency was 1 rad/s. The ${G}^{{\prime} }$ and ${G}^{{\prime} {\prime} }$ were recorded over a period of 10,800 s after mixing the proteins. Frequency-sweep measurements were subsequently performed at room temperature with the strain maintained at 5%. The relaxation spectra H(τ) were calculated from the frequency sweep data by

2 $${G}^{{\prime} }\left(\omega \right)={\int }_{\!\!\!\!\! -{\infty }}^{{\infty }}H(\tau )\frac{{w}^{2}{\tau }^{2}}{1+{w}^{2}{\tau }^{2}}{{{\rm{dln}}}}(\tau )$$

3 $${G}^{{\prime} {\prime} }\left(\omega \right)={\int }_{\!\!\!\!\! -{\infty }}^{{\infty }}H\left(\tau \right)\frac{w\tau }{1+{w}^{2}{\tau }^{2}}{{{\rm{dln}}}}\left(\tau \right)$$

A subsequent strain sweep was performed on each sample at 10 rad/s and 25 °C, with the strain amplitude varied from 0.1% to 300%.

### Software and computational tools

Sequence and structure analyses were performed using Foldseek (8979d230fb64c7089380b652758d8705493ed4a5), MMseqs2 (13-45111+ds-2), ESMFold (v2023_02), AlphaFold2 (v2.3.0), BLASTp (v2.12.0+), Rosetta (2024.09+release.06b3cf8), gmx_MMPBSA (v1.6.4), MDTraj (v1.9.9), ProteinMPNN (v1.0.1), Biopython (v1.79) and GROMACS (v2024.3). Data processing and visualization were performed using matplotlib (v3.5.3) and OriginLab (2026b). Custom scripts are available as described in the Code Availability section.

### Statistics and reproducibility

No statistical method was used to predetermine sample size. Computational sample sizes were selected to provide sufficiently large datasets for model training and evaluation and are specified in the corresponding Methods sections and figure legends. For conditional generation, 400 sequences were generated for each binding-energy condition in Fig. 4b, c, and 4000 sequences were generated for each condition for virtual screening. For unconditional-generation evaluation, 4000 designed sequences were analyzed.

The SDS-PAGE experiment was independently repeated three times with similar results. CD spectra were obtained by averaging three scans of each sample; these scans represent repeated measurements of the same sample rather than independent biological replicates. Hydrogel preparation and rheological tests were independently repeated three times with similar results. No data were excluded from the analyses unless otherwise stated. Blinding and randomized allocation to experimental groups were not applicable because the study did not involve treatment-group comparisons; the nine designs were randomly selected from predefined computational binding-energy groups as described above.

### Reporting summary

Further information on research design is available in the Nature Portfolio Reporting Summary linked to this article.

## Supplementary information

Supplementary information file

Reporting Summary

Transparent Peer Review file

## Source data

Source Data

## Data Availability

The UniRef50 clusters are available at https://www.uniprot.org/. Training sequences with their calculated features are available at https://github.com/daniel-dpq/TangleDiff.git. The ESMFold-predicted homodimeric structures of training sequences (with their random coil terminals cropped) are available at Figshare (https://doi.org/10.6084/m9.figshare.29604998.v1). Raw data used for all figures and tables are also available at https://doi.org/10.6084/m9.figshare.32658021. Raw SDS-PAGE data are available in the supplementary file. Source data are provided with this paper.

## Code availability

The code used for model training and inference, training-data screening, metric calculation and the evaluation pipeline is available at GitHub (https://github.com/daniel-dpq/TangleDiff) and has been archived in Zenodo as version 1.0.0 at https://doi.org/10.5281/zenodo.21866213^70^. Scripts for MD simulations and virtual screening of designed sequences prior to experiments are available at https://doi.org/10.6084/m9.figshare.32658021.
