# Supplementary material for: Generating protein hydrogels with customizable stress relaxation behavior via deep learning-driven entanglement design
Source: Nat Commun. 2026 Sep 21;17:11006. doi: 10.1038/s41467-026-77607-9 (PMC13687093; doi:10.1038/s41467-026-77607-9)
Supplement: Supplementary file 1 [file 41467_2026_77607_MOESM1_ESM.pdf]

Supplementary Information

Generating Protein Hydrogels with Customizable Stress Relaxation Behavior via Deep Learning-Driven Entanglement Design

Puqing Deng[1,†], Yutong Wu[1,†], Hong Kiu Francis Fok[1], Linyan Li[2], Wen-Bin Zhang[3, 4], Fei Sun[1,*], Hanyu Gao[1,*]

[1]Department of Chemical and Biological Engineering, Hong Kong University of Science and Technology, Clear Water Bay, Hong Kong

[2]Department of Data Science, City University of Hong Kong, Kowloon, Hong Kong.

[3]Beijing National Laboratory for Molecular Sciences, Key Laboratory of Polymer Chemistry & Physics of Ministry of Education, Center for Soft Matter Science and Engineering, College of Chemistry and Molecular Engineering, Peking University, Beijing 100871, P. R. China

[4]AI for Science (AI4S)-Preferred Program, Shenzhen Graduate School, Peking University, Shenzhen 518055, P. R. China

* To whom correspondence should be addressed. Email: hanyugao@ust.hk, kefsun@ust.hk

†: These authors contributed equally to this work.

Table of Contents

Supplementary Methods..2

Supplementary Figures..4

Supplementary Tables..24

Supplementary References..28

Supplementary Methods

Denoising Diffusion Probabilistic Models (DDPM)

Denoising Diffusion Probabilistic Models (DDPMs)[1] learn to reverse a gradual noising process to progressively transform random noise into generated data through a sequence of steps. To learn this reverse transition, a forward transition from $\boldsymbol{x_0}$ to $\boldsymbol{x_t}$ is first defined:

$$\boldsymbol{x_t} = \sqrt{\gamma(t)}\,\boldsymbol{x_0} + \sqrt{1-\gamma(t)}\,\boldsymbol{\epsilon}, \quad (1)$$

where $\boldsymbol{\epsilon} \sim \boldsymbol{\mathcal{N}}(\boldsymbol{0}, \boldsymbol{I})$, $t \sim \mathcal{U}(\boldsymbol{0}, \boldsymbol{T})$, $\gamma(\boldsymbol{t})$ is a decreasing function from 1 to 0. A neural network $f(\boldsymbol{x_t}, t)$ is trained to predict $\boldsymbol{x_0}$ (or $\boldsymbol{\epsilon}$) from $\boldsymbol{x_t}$, and one can estimate $\boldsymbol{x_{t-1}}$ from $\boldsymbol{x_t}$ and predicted $\widetilde{\boldsymbol{x}}_{\boldsymbol{0}}$ (or $\tilde{\boldsymbol{\epsilon}}$). The training target is the MSE between $\widetilde{\boldsymbol{x}}_{\boldsymbol{0}}$ and $\boldsymbol{x_0}$ (or $\tilde{\boldsymbol{\epsilon}}$ and $\boldsymbol{\epsilon}$):

$$\mathcal{L}_{x_0} = E_{t \sim U(0,T); \epsilon \sim \mathrm{N}(0,\mathrm{I})} \left| f\left(\sqrt{\gamma(t)}\,\boldsymbol{x_0} + \sqrt{1-\gamma(t)}\,\boldsymbol{\epsilon}, t\right) - \boldsymbol{x_0} \right|^2. \quad (2)$$

Then a sample $\boldsymbol{x_0}$ can be generated by transforming a random noise $\boldsymbol{x_T}$ through a series of steps: $\boldsymbol{x_T} \rightarrow \boldsymbol{x_{T-1}} \rightarrow \ldots \rightarrow \boldsymbol{x_1} \rightarrow \boldsymbol{x_0}$. At each step, $f(\boldsymbol{x_t}, t)$ is applied to predict $\boldsymbol{x_0}$ and transition rules[1,2] are applied to estimate $\boldsymbol{x_{t-1}}$.

Self-conditioning[3]

In the previously described denoising process, the predicted $\boldsymbol{x_0}$ at timestep t is simply discarded when predicting $\boldsymbol{x_0}$ at a new timestep. To utilize this information, $\widetilde{\boldsymbol{x}}_{\boldsymbol{0}}$ from the previous timestep is taken as the input when predicting $\boldsymbol{x_0}$ at the current timestep. Then the neural network becomes $f(\boldsymbol{x_t}, \widetilde{\boldsymbol{x}}_{\boldsymbol{0}}, t)$. During training, with a probability of 50%, $\widetilde{\boldsymbol{x}}_{\boldsymbol{0}}$ is set to $\boldsymbol{0}$, which falls back to modeling without self-conditioning.

Experimental validation of the structural entanglement

To experimentally validate the structural entanglement in C4_370, we designed the constructs (B-C4_370-A) using the gene cassette previously applied to catenane synthesis (Supplementary Fig. 19).[4,5] We purified the dimeric products and conducted tobacco etch virus (TEV) protease-mediated cleavage. The purified, partially digested and fully digested products were analyzed using SDS-PAGE.

Plasmids harbouring genes encoding the B-X-A proteins were transformed into *E. coli* BL21(DE3), and the cells were cultured in LB medium at 37 °C with shaking at 220 rpm until OD_{600} reached 0.5 - 0.6. Protein expression was induced with 200 µM IPTG at 30 °C for 4 h. The cells were harvested by centrifugation at 4,200 × *g* and 4 °C for 30 min. The cell pellets were resuspended in lysis buffer containing 300 mM NaCl and 50 mM Tris-HCl (pH 8.0) and stored at -80 °C overnight before protein purification.

The frozen cell pellets were thawed, resuspended in lysis buffer supplemented with 1 mM phenylmethylsulfonyl fluoride (PMSF), and lysed by sonication. The recombinant protein was purified using HisTrap HP Ni-NTA columns (Cytiva). To remove residual impurities, aggregates, imidazole and other small molecules, the partially purified sample was further purified by size-exclusion chromatography using a HiLoad 26/600 Superdex 200 pg column, yielding high-purity dimeric protein for subsequent experiments.

Site-specific proteolytic digestion using tobacco etch virus (TEV) protease was performed on SEC-purified recombinant B-X-A protein to verify its mechanically interlocked topological structure. Purified B-X-A protein was subjected to digestion using TEV protease. TEV protease was mixed with the protein

substrate at a molar ratio of 1:20. For partial digestion, the reaction mixture was incubated at 4 °C for 5 min. For complete digestion, the mixture was incubated overnight at 4 °C. All digestion products were subsequently analyzed by sodium dodecyl sulfate polyacrylamide gel electrophoresis (SDS-PAGE).

Supplementary Figures

green: The polyhistidine tag used for the affinity purification of proteins
red: SpyTag
orange: SpyCatcher
blue: Designed sequences
grey: TEV protease site

(1) B-B-B (MW: 54.9 kDa)

```
001 MKGSSHHHHHHVDIPTTENLYFQGAMVDTLSGLSSEQGQSGDMTIEEDSA
051 THIKFSKRDEDGKELAGATMELRDSSGKTISTWISDGQVKDFYLYPGKYT
101 FVETAAPDGYEVATAITFTVNEQGQVTVNGKATKGDAHIDGPQGIWGQLD
151 GHGVGVPGVGVPGVGVPGEGVPGVGVPGVGVPGVGVPGVGVPGEGVPGVG
201 VPGVGVPGVGVPGVGVPGEGVPGVGVPGVGELGRGDDSATHIKFSKRDED
251 GKELAGATMELRDSSGKTISTWISDGQVKDFYLYPGKYTFVETAAPDGYE
301 VATAITFTVNEQGQVTVNGTSVPGVGVPGVGVPGEGVPGVGVPGVGVPGV
351 GVPGVGVPGEGVPGVGVPGVGVPGVGVPGVGVPGEGVPGVGVPGVGVPGG
401 LLDIPTTENLYFQGAMVDTLSGLSSEQGQSGDMTIEEDSATHIKFSKRDE
451 DGKELAGATMELRDSSGKTISTWISDGQVKDFYLYPGKYTFVETAAPDGY
501 EVATAITFTVNEQGQVTVNGKATKGDAHIDGPQGIWGQLEWKK
```

(2) A-C0_203-A (MW: 24.5 kDa)

```
001 MKGSSHHHHHHVDAHIVMVDAYKPTKLDGHGVGVPGVGVPGVGVPGEGVP
051 GVGVPGVGVPGVGVPGVGVPGEGVPGVGVPGVGVPGVGVPGVGVPGEGVP
101 GVGVPGVGELMVIVRELKFITKVEEKGRIIIPKRVREVLGIGEGDEVEII
151 IKKIGGTSVPGVGVPGVGVPGEGVPGVGVPGVGVPGVGVPGVGVPGEGVP
201 GVGVPGVGVPGVGVPGVGVPGEGVPGVGVPGVGVPGGLLDAHIVMVDAYK
251 PTKLEWKK
```

(3) B-C0_203-B (MW: 50.7 kDa)

```
001 MKGSSHHHHHHVDIPTTENLYFQGAMVDTLSGLSSEQGQSGDMTIEEDSA
051 THIKFSKRDEDGKELAGATMELRDSSGKTISTWISDGQVKDFYLYPGKYT
101 FVETAAPDGYEVATAITFTVNEQGQVTVNGKATKGDAHIDGPQGIWGQLD
151 GHGVGVPGVGVPGVGVPGEGVPGVGVPGVGVPGVGVPGVGVPGEGVPGVG
201 VPGVGVPGVGVPGVGVPGEGVPGVGVPGVGELMVIVRELKFITKVEEKGR
251 IIIPKRVREVLGIGEGDEVEIIIKKIGGTSVPGVGVPGVGVPGEGVPGVG
301 VPGVGVPGVGVPGVGVPGEGVPGVGVPGVGVPGVGVPGVGVPGEGVPGVG
351 VPGVGVPGGLLDIPTTENLYFQGAMVDTLSGLSSEQGQSGDMTIEEDSAT
401 HIKFSKRDEDGKELAGATMELRDSSGKTISTWISDGQVKDFYLYPGKYTF
451 VETAAPDGYEVATAITFTVNEQGQVTVNGKATKGDAHIDGPQGIWGQLEW
501 KK
```

(4) A-C0_1887-A (MW: 24.2 kDa)

```
001 MKGSSHHHHHHVDAHIVMVDAYKPTKLDGHGVGVPGVGVPGVGVPGEGVP
051 GVGVPGVGVPGVGVPGVGVPGEGVPGVGVPGVGVPGVGVPGVGVPGEGVP
101 GVGVPGVGELMKEVEVIANIRVPAGITDELIEEAFLAFARDNGWEVGGGE
151 IEEVETSVPGVGVPGVGVPGEGVPGVGVPGVGVPGVGVPGVGVPGEGVPG
201 VGVPGVGVPGVGVPGVGVPGEGVPGVGVPGVGVPGGLLDAHIVMVDAYKP
251 TKLEWKK
```

(5) B-C0_1887-B (MW: 51.0 kDa)

001 MKGSSHHHHHHVDIPTTENLYFQGAMVDTLSGLSSEQGQSGDMTIEEDSA
051 THIKFSKRDEDGKELAGATMELRDSSGKTISTWISDGQVKDFYLYPGKYT
101 FVETAAPDGYEVATAITFTVNEQGQVTVNGKATKGDAHIDGPQGIWGQLD
151 GHGVGVPGVGVPGVGVPGEGVPGVGVPGVGVPGVGVPGVGVPGEGVPGVG
201 VPGVGVPGVGVPGVGVPGEGVPGVGVPGVGELMKEVEVIANIRVPAGITD
251 ELIEEAFLAFARDNGWEVGGGEIEEVETSVPGVGVPGVGVPGEGVPGVGV
301 PGVGVPGVGVPGVGVPGEGVPGVGVPGVGVPGVGVPGVGVPGEGVPGVGV
351 PGVGVPGGLLDIPTTENLYFQGAMVDTLSGLSSEQGQSGDMTIEEDSATH
401 IKFSKRDEDGKELAGATMELRDSSGKTISTWISDGQVKDFYLYPGKYTFV
451 ETAAPDGYEVATAITFTVNEQGQVTVNGKATKGDAHIDGPQGIWGQLEWK
501 k

(6) A-C0_2898-A (MW: 25.0 kDa)
001 MKGSSHHHHHHVDAHIVMVDAYKPTKLDGHGVGVPGVGVPGVGVPGEGVP
051 GVGVPGVGVPGVGVPGVGVPGEGVPGVGVPGVGVPGVGVPGVGVPGEGVP
101 GVGVPGVGELMLYRVTLEERVRRTIEVEARSAQEAENIALELVEDGYEVA
151 DIEIVEIRETSVPGVGVPGVGVPGEGVPGVGVPGVGVPGVGVPGVGVPGE
201 GVPGVGVPGVGVPGVGVPGVGVPGEGVPGVGVPGVGVPGGLLDAHIVMVD
251 AYKPTKLEWKK

(7) B-C0_2898-B (MW: 51.2 kDa)
001 MKGSSHHHHHHVDIPTTENLYFQGAMVDTLSGLSSEQGQSGDMTIEEDSA
051 THIKFSKRDEDGKELAGATMELRDSSGKTISTWISDGQVKDFYLYPGKYT
101 FVETAAPDGYEVATAITFTVNEQGQVTVNGKATKGDAHIDGPQGIWGQLD
151 GHGVGVPGVGVPGVGVPGEGVPGVGVPGVGVPGVGVPGVGVPGEGVPGVG
201 VPGVGVPGVGVPGVGVPGEGVPGVGVPGVGELMLYRVTLEERVRRTIEVE
251 ARSAQEAENIALELVEDGYEVADIEIVEIRETSVPGVGVPGVGVPGEGVP
301 GVGVPGVGVPGVGVPGVGVPGEGVPGVGVPGVGVPGVGVPGVGVPGEGVP
351 GVGVPGVGVPGGLLDIPTTENLYFQGAMVDTLSGLSSEQGQSGDMTIEED
401 SATHIKFSKRDEDGKELAGATMELRDSSGKTISTWISDGQVKDFYLYPGK
451 YTFVETAAPDGYEVATAITFTVNEQGQVTVNGKATKGDAHIDGPQGIWGQ
501 LEWKK

(8) A-C2_1488-A (MW: 24.6 kDa)
001 MKGSSHHHHHHVDAHIVMVDAYKPTKLDGHGVGVPGVGVPGVGVPGEGVP
051 GVGVPGVGVPGVGVPGVGVPGEGVPGVGVPGVGVPGVGVPGVGVPGEGVP
101 GVGVPGVGELMRATNLYIDADLLAQARDLGLNVSAVAEEALRREIEAARR
151 RRLREAATSVPGVGVPGVGVPGEGVPGVGVPGVGVPGVGVPGVGVPGEGV
201 PGVGVPGVGVPGVGVPGVGVPGEGVPGVGVPGVGVPGGLLDAHIVMVDAY
251 KPTKLEWKK

(9) B-C2_1488-B (MW: 50.8 kDa)
001 MKGSSHHHHHHVDIPTTENLYFQGAMVDTLSGLSSEQGQSGDMTIEEDSA
051 THIKFSKRDEDGKELAGATMELRDSSGKTISTWISDGQVKDFYLYPGKYT
101 FVETAAPDGYEVATAITFTVNEQGQVTVNGKATKGDAHIDGPQGIWGQLD
151 GHGVGVPGVGVPGVGVPGEGVPGVGVPGVGVPGVGVPGVGVPGEGVPGVG
201 VPGVGVPGVGVPGVGVPGEGVPGVGVPGVGELMRATNLYIDADLLAQARD
251 LGLNVSAVAEEALRREIEAARRRRLREAATSVPGVGVPGVGVPGEGVPGV
301 GVPGVGVPGVGVPGVGVPGEGVPGVGVPGVGVPGVGVPGVGVPGEGVPGV

351 GVPGVGVPGGLLDIPTTENLYFQGAMVDTLSGLSSEQGQSGDMTIEEDSA
401 THIKFSKRDEDGKELAGATMELRDSSGKTISTWISDGQVKDFYLYPGKYT
451 FVETAAPDGYEVATAITFTVNEQGQVTVNGKATKGDAHIDGPQGIWGQLE
501 WKK

(10) A-C2_2351-A (MW: 24.6 kDa)

001 MKGSSHHHHHHVDAHIVMVDAYKPTKLDGHGVGVPGVGVPGVGVPGEGVP
051 GVGVPGVGVPGVGVPGVGVPGEGVPGVGVPGVGVPGVGVPGVGVPGEGVP
101 GVGVPGVGELMVQYPLRMPSELKAKLDEIAKLNGRSLNAEIVQILREYVQ
151 RREQQTSVPGVGVPGVGVPGEGVPGVGVPGVGVPGVGVPGVGVPGEGVPG
201 VGVPGVGVPGVGVPGVGVPGEGVPGVGVPGVGVPGGLLDAHIVMVDAYKP
251 TKLEWKK

(11) B-C2_2351-B (MW: 50.8 kDa)

001 MKGSSHHHHHHVDIPTTENLYFQGAMVDTLSGLSSEQGQSGDMTIEEDSA
051 THIKFSKRDEDGKELAGATMELRDSSGKTISTWISDGQVKDFYLYPGKYT
101 FVETAAPDGYEVATAITFTVNEQGQVTVNGKATKGDAHIDGPQGIWGQLD
151 GHGVGVPGVGVPGVGVPGEGVPGVGVPGVGVPGVGVPGVGVPGEGVPGVG
201 VPGVGVPGVGVPGVGVPGEGVPGVGVPGVGELMVQYPLRMPSELKAKLDE
251 IAKLNGRSLNAEIVQILREYVQRREQQTSVPGVGVPGVGVPGEGVPGVGV
301 PGVGVPGVGVPGVGVPGEGVPGVGVPGVGVPGVGVPGVGVPGEGVPGVGV
351 PGVGVPGGLLDIPTTENLYFQGAMVDTLSGLSSEQGQSGDMTIEEDSATH
401 IKFSKRDEDGKELAGATMELRDSSGKTISTWISDGQVKDFYLYPGKYTFV
451 ETAAPDGYEVATAITFTVNEQGQVTVNGKATKGDAHIDGPQGIWGQLEWK
501 K

(12) A-C2_1020-A (MW: 24.7 kDa)

001 MKGSSHHHHHHVDAHIVMVDAYKPTKLDGHGVGVPGVGVPGVGVPGEGVP
051 GVGVPGVGVPGVGVPGVGVPGEGVPGVGVPGVGVPGVGVPGVGVPGEGVP
101 GVGVPGVGELMKSIQLRIPEKMMEEIETIAEKKGYGTAADAIREYVRKGM
151 EIEKKAATSVPGVGVPGVGVPGEGVPGVGVPGVGVPGVGVPGVGVPGEGV
201 PGVGVPGVGVPGVGVPGVGVPGEGVPGVGVPGVGVPGGLLDAHIVMVDAY
251 KPTKLEWKK

(13) B-C2_1020-B (MW: 50.8 kDa)

001 MKGSSHHHHHHVDIPTTENLYFQGAMVDTLSGLSSEQGQSGDMTIEEDSA
051 THIKFSKRDEDGKELAGATMELRDSSGKTISTWISDGQVKDFYLYPGKYT
101 FVETAAPDGYEVATAITFTVNEQGQVTVNGKATKGDAHIDGPQGIWGQLD
151 GHGVGVPGVGVPGVGVPGEGVPGVGVPGVGVPGVGVPGVGVPGEGVPGVG
201 VPGVGVPGVGVPGVGVPGEGVPGVGVPGVGELMKSIQLRIPEKMMEEIET
251 IAEKKGYGTAADAIREYVRKGMEIEKKAATSVPGVGVPGVGVPGEGVPGV
301 GVPGVGVPGVGVPGVGVPGEGVPGVGVPGVGVPGVGVPGVGVPGEGVPGV
351 GVPGVGVPGGLLDIPTTENLYFQGAMVDTLSGLSSEQGQSGDMTIEEDSA
401 THIKFSKRDEDGKELAGATMELRDSSGKTISTWISDGQVKDFYLYPGKYT
451 FVETAAPDGYEVATAITFTVNEQGQVTVNGKATKGDAHIDGPQGIWGQLE

501 WKK

(14) A-C4_370-A (MW: 24.9 kDa)

001 MKGSSHHHHHHVDAHIVMVDAYKPTKLDGHGVGVPGVGVPGVGVPGEGVP
051 GVGVPGVGVPGVGVPGVGVPGEGVPGVGVPGVGVPGVGVPGVGVPGEGVP
101 GVGVPGVGELMTRIPVSTALRDELAALKRDGETYAEVIARLVEERQRQQL
151 ARDIERAAGTSVPGVGVPGVGVPGEGVPGVGVPGVGVPGVGVPGVGVPGE
201 GVPGVGVPGVGVPGVGVPGVGVPGEGVPGVGVPGVGVPGGLLDAHIVMVD
251 AYKPTKLEWKK

(15) B-C4_370-B (MW: 51.0 kDa)

001 MKGSSHHHHHHVDIPTTENLYFQGAMVDTLSGLSSEQGQSGDMTIEEDSA
051 THIKFSKRDEDGKELAGATMELRDSSGKTISTWISDGQVKDFYLYPGKYT
101 FVETAAPDGYEVATAITFTVNEQGQVTVNGKATKGDAHIDGPQGIWGQLD
151 GHGVGVPGVGVPGVGVPGEGVPGVGVPGVGVPGVGVPGVGVPGEGVPGVG
201 VPGVGVPGVGVPGVGVPGEGVPGVGVPGVGELMTRIPVSTALRDELAALK
251 RDGETYAEVIARLVEERQRQQLARDIERAAGTSVPGVGVPGVGVPGEGVP
301 GVGVPGVGVPGVGVPGVGVPGEGVPGVGVPGVGVPGVGVPGVGVPGEGVP
351 GVGVPGVGVPGGLLDIPTTENLYFQGAMVDTLSGLSSEQGQSGDMTIEED
401 SATHIKFSKRDEDGKELAGATMELRDSSGKTISTWISDGQVKDFYLYPGK
451 YTFVETAAPDGYEVATAITFTVNEQGQVTVNGKATKGDAHIDGPQGIWGQ
501 LEWKK

(16) A-C4_1771-A (MW: 24.8 kDa)

001 MKGSSHHHHHHVDAHIVMVDAYKPTKLDGHGVGVPGVGVPGVGVPGEGVP
051 GVGVPGVGVPGVGVPGVGVPGEGVPGVGVPGVGVPGVGVPGVGVPGEGVP
101 GVGVPGVGELMTRIPTSSALKAALASLREPGETANDVLARLIEERRRQAI
151 LAMAQKIAEETSVPGVGVPGVGVPGEGVPGVGVPGVGVPGVGVPGVGVPG
201 EGVPGVGVPGVGVPGVGVPGVGVPGEGVPGVGVPGVGVPGGLLDAHIVMV
251 DAYKPTKLEWKK

(17) B-C4_1771-B (MW: 50.95 kDa)

001 MKGSSHHHHHHVDIPTTENLYFQGAMVDTLSGLSSEQGQSGDMTIEEDSA
051 THIKFSKRDEDGKELAGATMELRDSSGKTISTWISDGQVKDFYLYPGKYT
101 FVETAAPDGYEVATAITFTVNEQGQVTVNGKATKGDAHIDGPQGIWGQLD
151 GHGVGVPGVGVPGVGVPGEGVPGVGVPGVGVPGVGVPGVGVPGEGVPGVG
201 VPGVGVPGVGVPGVGVPGEGVPGVGVPGVGELMTRIPTSSALKAALASLR
251 EPGETANDVLARLIEERRRQAILAMAQKIAEETSVPGVGVPGVGVPGEGV
301 PGVGVPGVGVPGVGVPGVGVPGEGVPGVGVPGVGVPGVGVPGVGVPGEGV
351 PGVGVPGVGVPGGLLDIPTTENLYFQGAMVDTLSGLSSEQGQSGDMTIEE
401 DSATHIKFSKRDEDGKELAGATMELRDSSGKTISTWISDGQVKDFYLYPG
451 KYTFVETAAPDGYEVATAITFTVNEQGQVTVNGKATKGDAHIDGPQGIWG
501 QLEWKK

(18) A-C4_2636-A (MW: 25.0 kDa)

001 MKGSSHHHHHHVDAHIVMVDAYKPTKLDGHGVGVPGVGVPGVGVPGEGVP
051 GVGVPGVGVPGVGVPGVGVPGEGVPGVGVPGVGVPGVGVPGVGVPGEGVP
101 GVGVPGVGELMTTIRVSTETRDALAALKRDGESYNDVIARLIEQRAREEF
151 GAAMKQIAEDTSVPGVGVPGVGVPGEGVPGVGVPGVGVPGVGVPGVGVPG

201 EGVPGVGVPGVGVPGVGVPGVGVPGEGVPGVGVPGVGVPGGLLDAHIVMV
251 DAYKPTKLEWKK

(19) B-C4_2636-B (MW: 51.1 kDa)
001 MKGSSHHHHHHVDIPTTENLYFQGAMVDTLSGLSSEQGQSGDMTIEEDSA
051 THIKFSKRDEDGKELAGATMELRDSSGKTISTWISDGQVKDFYLYPGKYT
101 FVETAAPDGYEVATAITFTVNEQGQVTVNGKATKGDAHIDGPQGIWGQLD
151 GHGVGVPGVGVPGVGVPGEGVPGVGVPGVGVPGVGVPGVGVPGEGVPGVG
201 VPGVGVPGVGVPGVGVPGEGVPGVGVPGVGELMTTIRVSTETRDALAALK
251 RDGESYNDVIARLIEQRAREEFGAAMKQIAEDTSVPGVGVPGVGVPGEGV
301 PGVGVPGVGVPGVGVPGVGVPGEGVPGVGVPGVGVPGVGVPGVGVPGEGV
351 PGVGVPGVGVPGGLLDIPTTENLYFQGAMVDTLSGLSSEQGQSGDMTIEE
401 DSATHIKFSKRDEDGKELAGATMELRDSSGKTISTWISDGQVKDFYLYPG
451 KYTFVETAAPDGYEVATAITFTVNEQGQVTVNGKATKGDAHIDGPQGIWG
501 QLEWKK

(20) B-C0_2898-A (MW: 26.1 kDa)
001 MRGSSHHHHHHVEASDIPTTENLYRQGAMVDTLSGLSSEQGQSGDMTIEE
051 DSATHIKFSKRDEDGKELAGATMELRDSSGKTISTWISDGQVKDFYLYPG
101 KYTFVETAAPDGYEVATAITFTVNEQGQVTVNGKATKGDAHIDGPQGIWG
151 QENLYFQGELGSGSMLYRVTLEERVRRTIEVEARSAQEAENIALELVEDG
201 YEVADIEIVEIRESGGSGAHIVMVDAYKPTKGTVEKKM

(21) B-C0_1887-A (MW: 25.3 kDa)
001 MRGSSHHHHHHVEASDIPTTENLYRQGAMVDTLSGLSSEQGQSGDMTIEE
051 DSATHIKFSKRDEDGKELAGATMELRDSSGKTISTWISDGQVKDFYLYPG
101 KYTFVETAAPDGYEVATAITFTVNEQGQVTVNGKATKGDAHIDGPQGIWG
151 QENLYFQGELGSGSMKEVEVIANIRVPAGITDELIEEAFLAFARDNGWEV
201 GGGEIEEVESGGSGAHIVMVDAYKPTKGTVEKKM

(22) B-C0_203-A (MW: 25.5 kDa)
001 MRGSSHHHHHHVEASDIPTTENLYRQGAMVDTLSGLSSEQGQSGDMTIEE
051 DSATHIKFSKRDEDGKELAGATMELRDSSGKTISTWISDGQVKDFYLYPG
101 KYTFVETAAPDGYEVATAITFTVNEQGQVTVNGKATKGDAHIDGPQGIWG
151 QENLYFQGELGSGSMVIVRELKFITKVEEKGRIIIPKRVREVLGIGEGDE
201 VEIIIKKIGGSGGSGAHIVMVDAYKPTKGTVEKKM

(23) B-C2_1488-A (MW: 25.6 kDa)
001 MRGSSHHHHHHVEASDIPTTENLYRQGAMVDTLSGLSSEQGQSGDMTIEE
051 DSATHIKFSKRDEDGKELAGATMELRDSSGKTISTWISDGQVKDFYLYPG
101 KYTFVETAAPDGYEVATAITFTVNEQGQVTVNGKATKGDAHIDGPQGIWG
151 QENLYFQGELGSGSMRATNLYIDADLLAQARDLGLNVSAVAEEALRREIE
201 AARRRRLREAASGGSGAHIVMVDAYKPTKGTVEKKM

(24) B-C2_2351_A (MW: 25.7 kDa)
001 MRGSSHHHHHHVEASDIPTTENLYRQGAMVDTLSGLSSEQGQSGDMTIEE
051 DSATHIKFSKRDEDGKELAGATMELRDSSGKTISTWISDGQVKDFYLYPG
101 KYTFVETAAPDGYEVATAITFTVNEQGQVTVNGKATKGDAHIDGPQGIWG
151 QENLYFQGELGSGSMVQYPLRMPSELKAKLDEIAKLNGRSLNAEIVQILR

201 EYVQRREQQSGGSGAHIVMVDAYKPTKGTVEKKM

(25) B-C2_1020-A (MW: 25.7 kDa)
001 MRGSSHHHHHHVEASDIPTTENLYRQGAMVDTLSGLSSEQGQSGDMTIEE
051 DSATHIKFSKRDEDGKELAGATMELRDSSGKTISTWISDGQVKDFYLYPG
101 KYTFVETAAPDGYEVATAITFTVNEQGQVTVNGKATKGDAHIDGPQGIWG
151 QENLYFQGELGSGSMKSIQLRIPEKMMEEIETIAEKKGYGTAADAIREYV
201 RKGMEIEKKAASGGSGAHIVMVDAYKPTKGTVEKKM

(26) B-C4_2636-A (MW: 25.9 kDa)
001 MRGSSHHHHHHVEASDIPTTENLYRQGAMVDTLSGLSSEQGQSGDMTIEE
051 DSATHIKFSKRDEDGKELAGATMELRDSSGKTISTWISDGQVKDFYLYPG
101 KYTFVETAAPDGYEVATAITFTVNEQGQVTVNGKATKGDAHIDGPQGIWG
151 QENLYFQGELGSGSMTTIRVSTETRDALAALKRDGESYNDVIARLIEQRA
201 REEFGAAMKQIAEDSGGSGAHIVMVDAYKPTKGTVEKKM

(27) B-C4_370-A (MW: 25.9 kDa)
001 MRGSSHHHHHHVEASDIPTTENLYRQGAMVDTLSGLSSEQGQSGDMTIEE
051 DSATHIKFSKRDEDGKELAGATMELRDSSGKTISTWISDGQVKDFYLYPG
101 KYTFVETAAPDGYEVATAITFTVNEQGQVTVNGKATKGDAHIDGPQGIWG
151 QENLYFQGELGSGSMTRIPVSTALRDELAALKRDGETYAEVIARLVEERQ
201 RQQLARDIERAAGSGGSGAHIVMVDAYKPTKGTVEKKM

(28) A-A-A (MW: 20.8 kDa)
001 MKGSSHHHHHHVDAHIVMVDAYKPTKLDGHGVGVPGVGVPGVGVPGEGVP
051 GVGVPGVGVPGVGVPGVGVPGEGVPGVGVPGVGVPGVGVPGVGVPGEGVP
101 GVGVPGVGELAHIVMVDAYKPTKTSVPGVGVPGVGVPGEGVPGVGVPGVG
151 VPGVGVPGVGVPGEGVPGVGVPGVGVPGVGVPGVGVPGEGVPGVGVPGVG
201 VPGGLLDAHIVMVDAYKPTKLEWKK

Supplementary Fig. 1. Amino acid sequences of designed constructs and their corresponding molecular weights (MW). Segments are highlighted in different colors corresponding to the gene-cassette schematic.

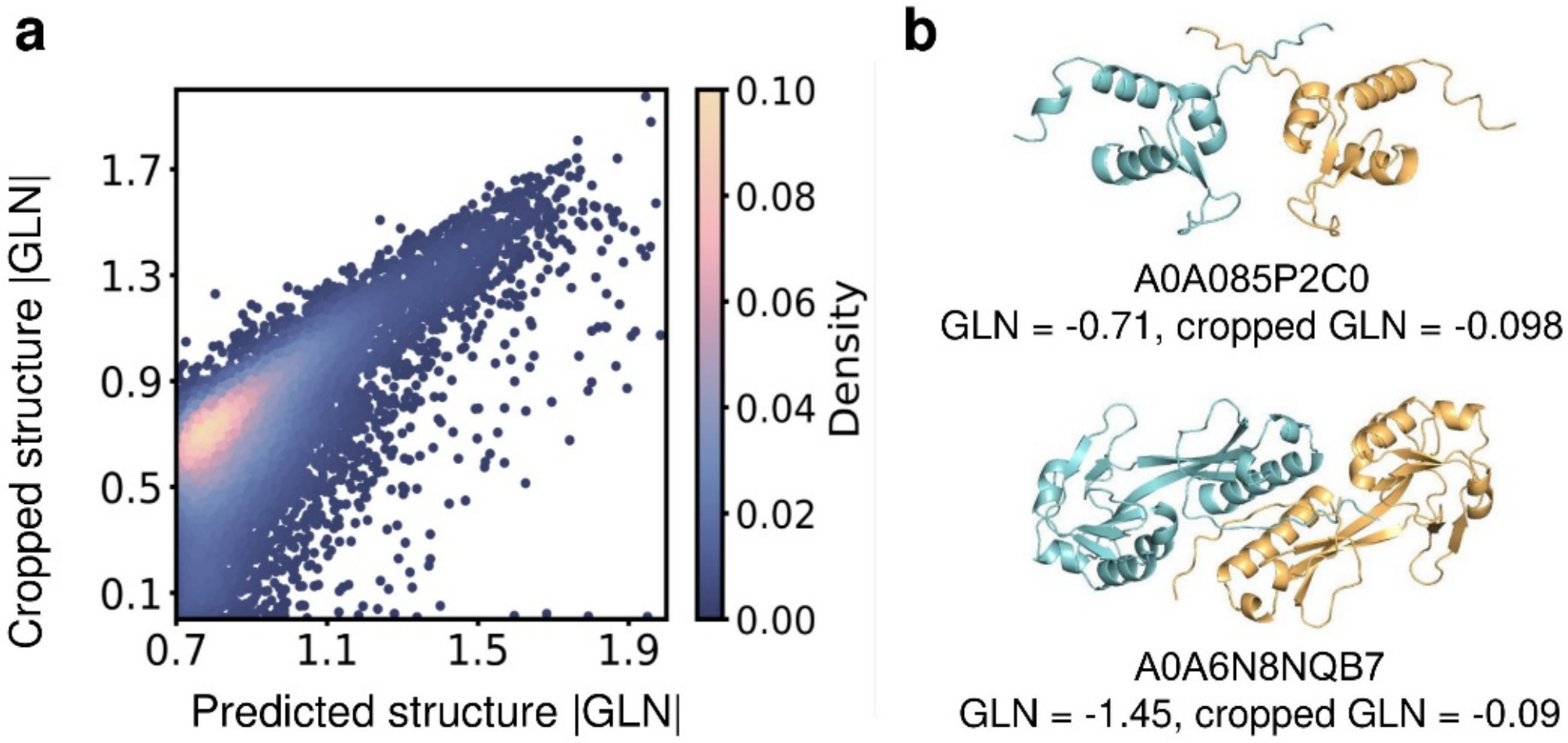

Supplementary Fig. 2. Random coil terminal cropping. (a) A certain number of structures are entangled prior to cropping but become disentangled afterward. (large |GLN| but small cropped |GLN|). (b) Structure examples in which entanglements are formed by random coil terminals, with large |GLN| but small cropped |GLN|.

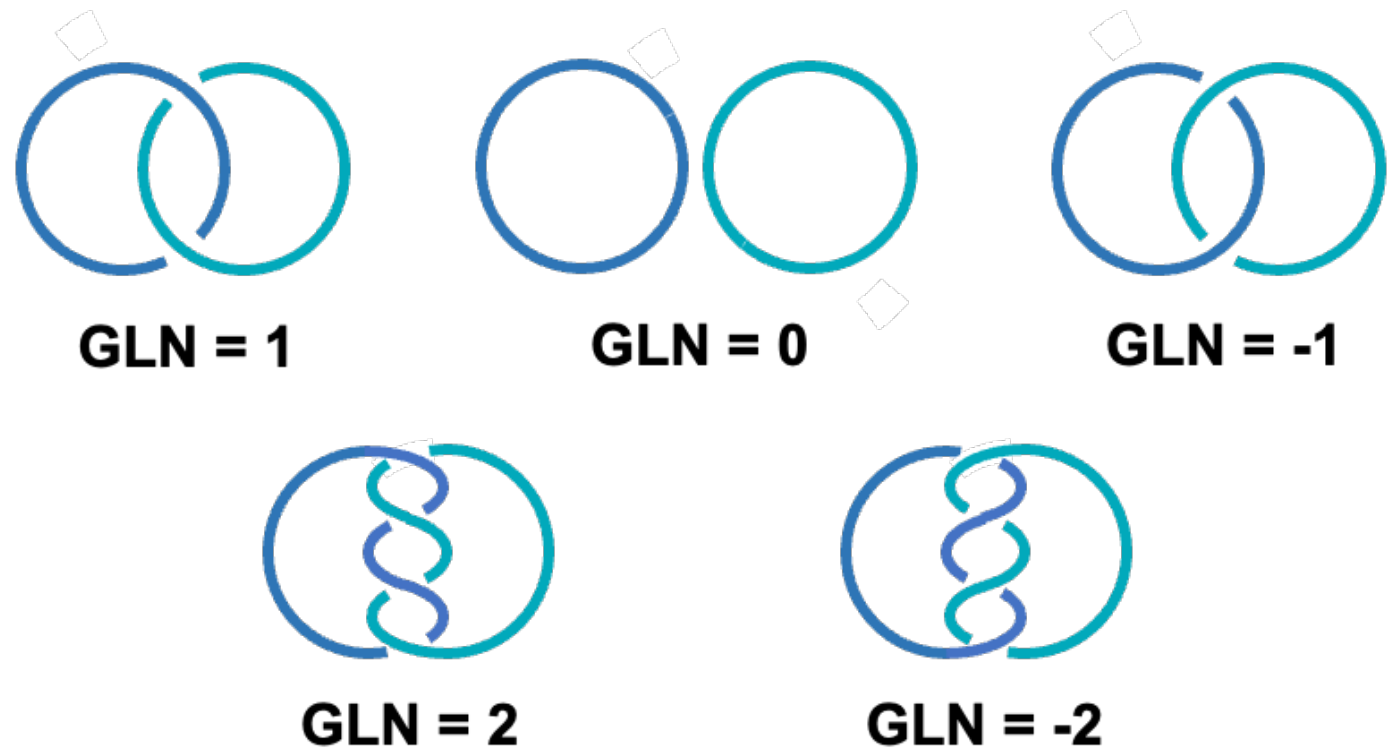

Supplementary Fig. 3. Gauss Linking Number for two closed curves. The absolute value quantifies the number of times that two closed curves wind around each other. The sign indicates the winding direction.

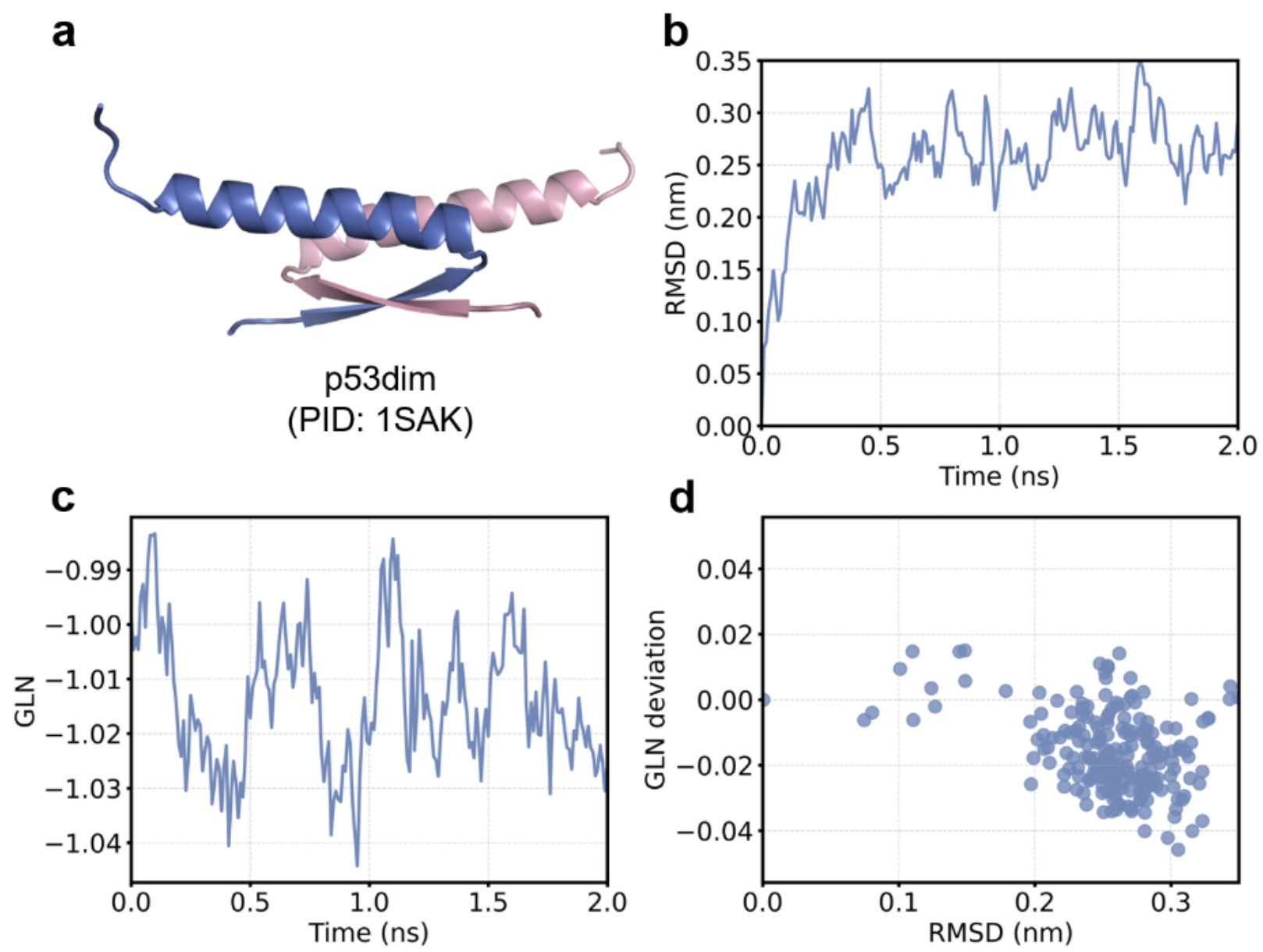

Supplementary Fig. 4. Sensitivity analysis of GLN values against structural deviations (e.g. thermal fluctuations). (a) The structure of p53dim, a typical intertwined homodimeric domain used for topological engineering. (b) Root mean square deviation (RMSD) versus time during a 2 ns molecular simulation of p53dim at 300 K. (c) Gauss Linking Number (GLN) versus time during a 2 ns molecular simulation of p53dim at 300 K. (d) Correlation between RMSD and GLN deviation. GLN changes little during the simulation.

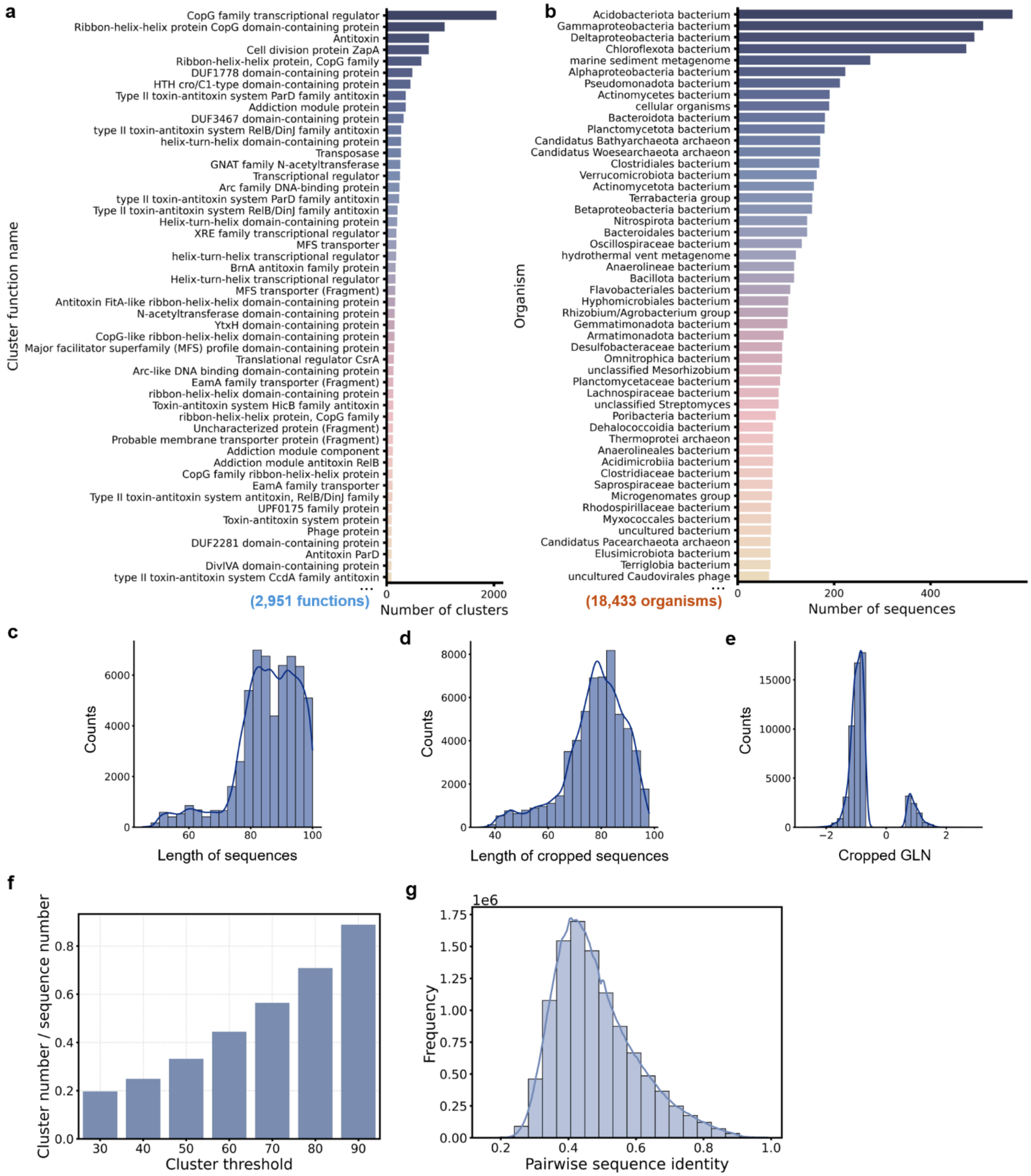

Supplementary Fig. 5. Statistics of entangled homodimer data. (a) Top 50 most popular cluster function labels in 32,339 discovered clusters. (b) Top 50 most popular organisms from which the 79,890 discovered entangled homodimers originate. Some sequences are not annotated with a specific organism in the UniProt database but are categorized broadly as cellular organisms (living entities composed of one or more cells, which are distinguished from non-cellular entities such as viruses). (c) Sequence length distribution of discovered entangled homodimers before the random coil terminal cropping. (d) Sequence length distribution of discovered entangled homodimers after the random coil terminal cropping. (e) Distribution of GLN computed after the random coil terminal cropping. (f) The number of training clusters / the number of training sequences when clustered at various thresholds (minimum sequence identity between clusters). (g) Distribution of pairwise sequence identities among training sequences.

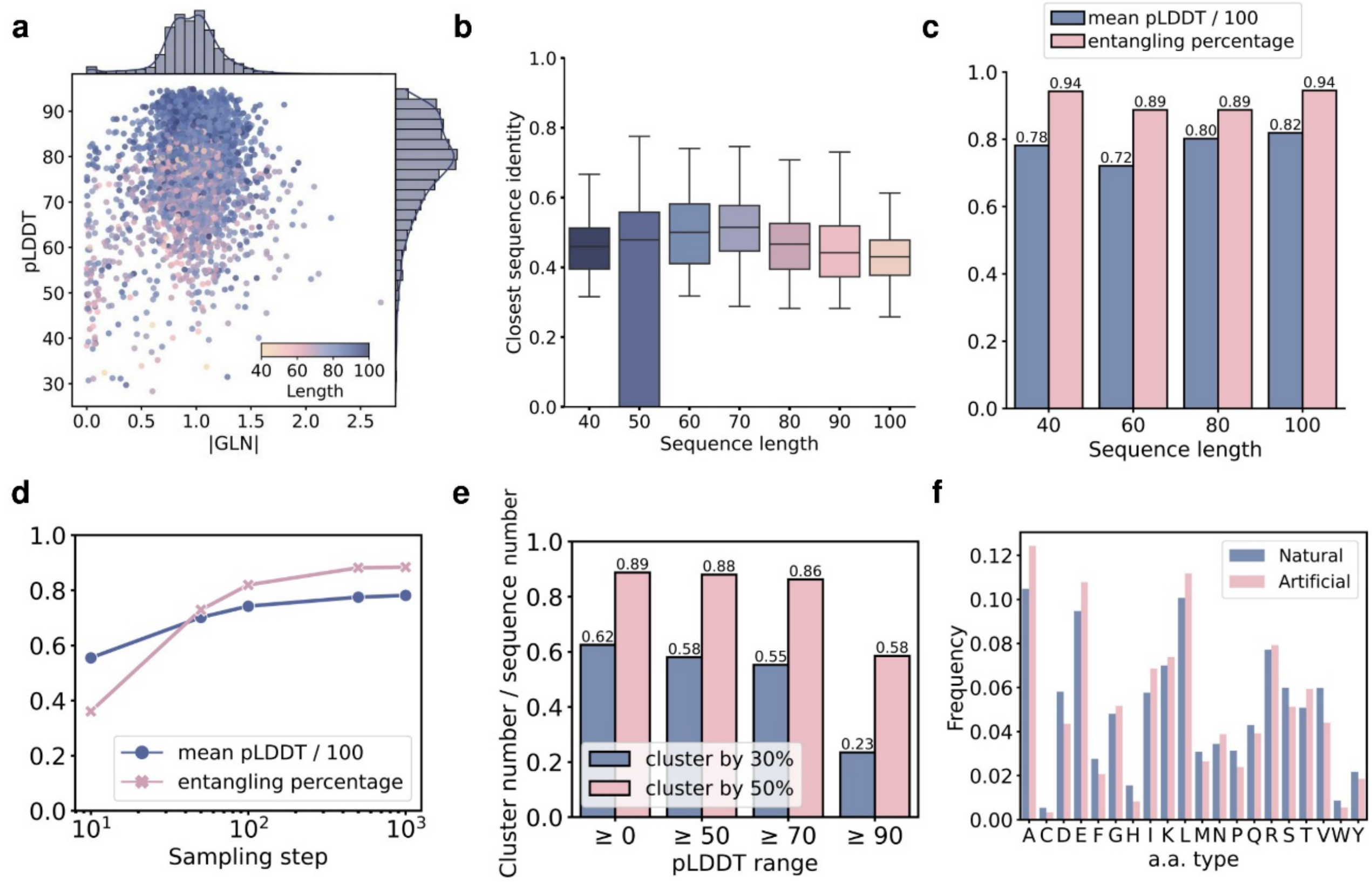

Supplementary Fig. 6. Extra data for unconditional generation by TangleDiff. (a) pLDDT by ESMFold versus |GLN| of predicted structures for 4,000 unconditionally generated designs. (b) TangleDiff generates novel sequences across different lengths. 400 designs were generated for each length. (c) Unconditional designs demonstrate high foldability and entangling percentage under different length constraints. 400 sequences are sampled and measured for each length. (d) Unconditional generation performance versus different numbers of sampling steps. TangleDiff uses 1,000 sampling steps by default. (e) Unconditional designs are diverse as quantified by the ratio of the number of clusters to the number of sequences when subjected to a sequence clustering at a threshold of 30% and 50% in different pLDDT ranges. 400 generated sequences are used for clustering. Sequence clustering is done by MMseqs2[6]. (f) Generated sequences show a similar amino-acid distribution to natural sequences.

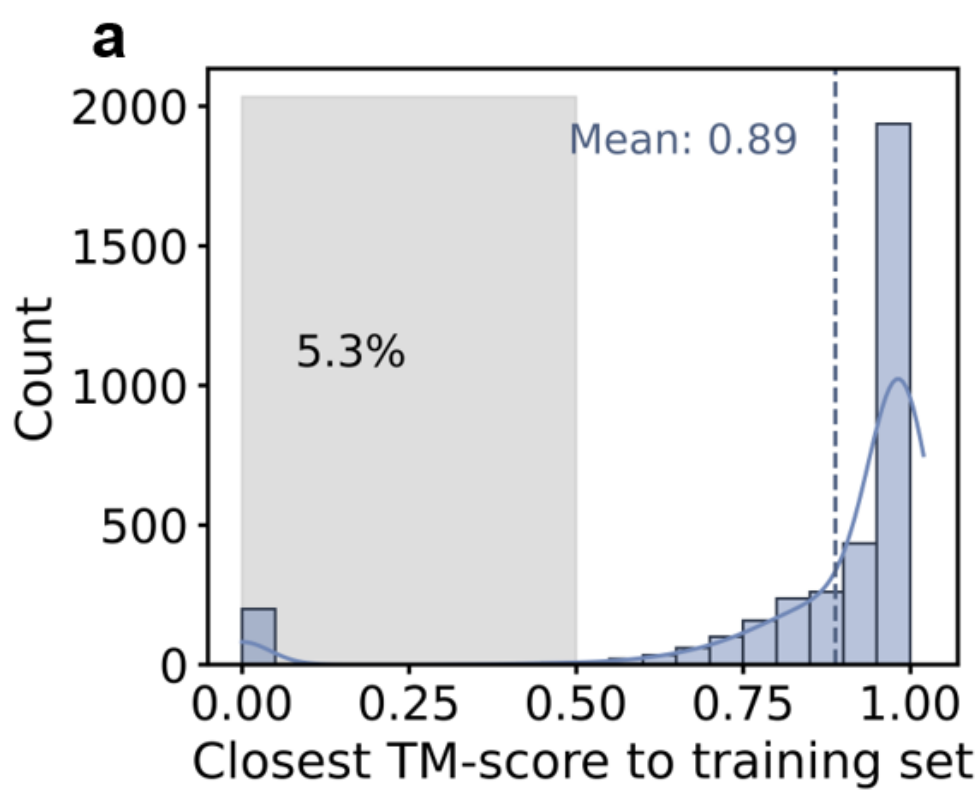

Supplementary Fig. 7. Structural distance of 4,000 unconditionally sampled designs to the (a) training structures and (b) PDB database. The closest TM-score was reported by Foldseek.[7]

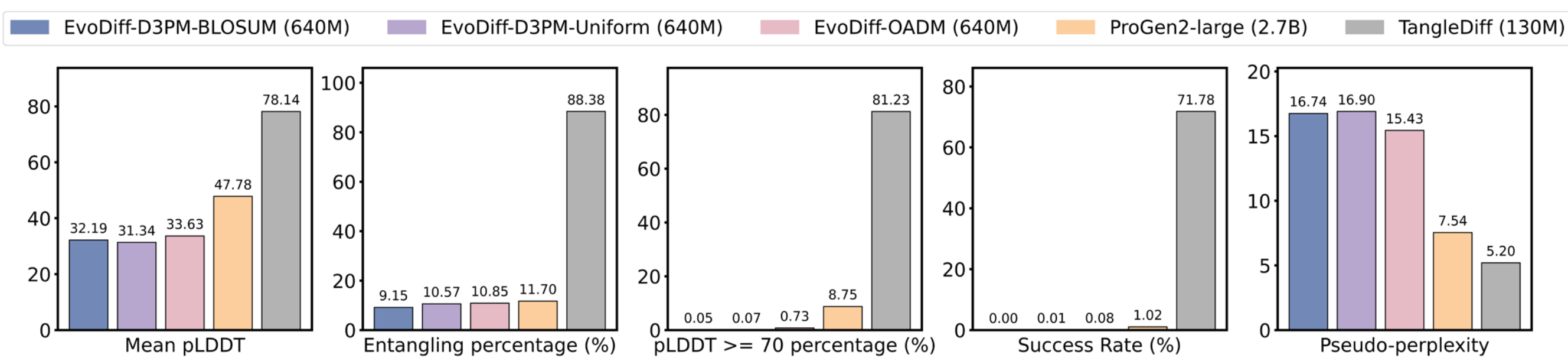

Supplementary Fig. 8. Generation performance of TangleDiff compared to other sequence generation models, including EvoDiff[8] and ProGen2[9]. TangleDiff outperformed the other models in both sequence quality and entangling percentage, achieving a success rate of 71.78% (percentage of designs that are both entangled and have a pLDDT > 70), significantly higher than the next best model, which achieved only 1.02%. All structures were predicted using ESMFold. Pseudo-perplexity is calculated by averaging the prediction probabilities provided by ESM2-650M across all positions in a sequence. This metric estimates how well the masked language model (in this case, ESM2-650M) comprehends the sequence. A lower pseudo-perplexity suggests that the sequence appears more natural and predictable to the model, while a higher pseudo-perplexity indicates that the sequence is unusual or unexpected. 4,000 sequences were generated for each model.

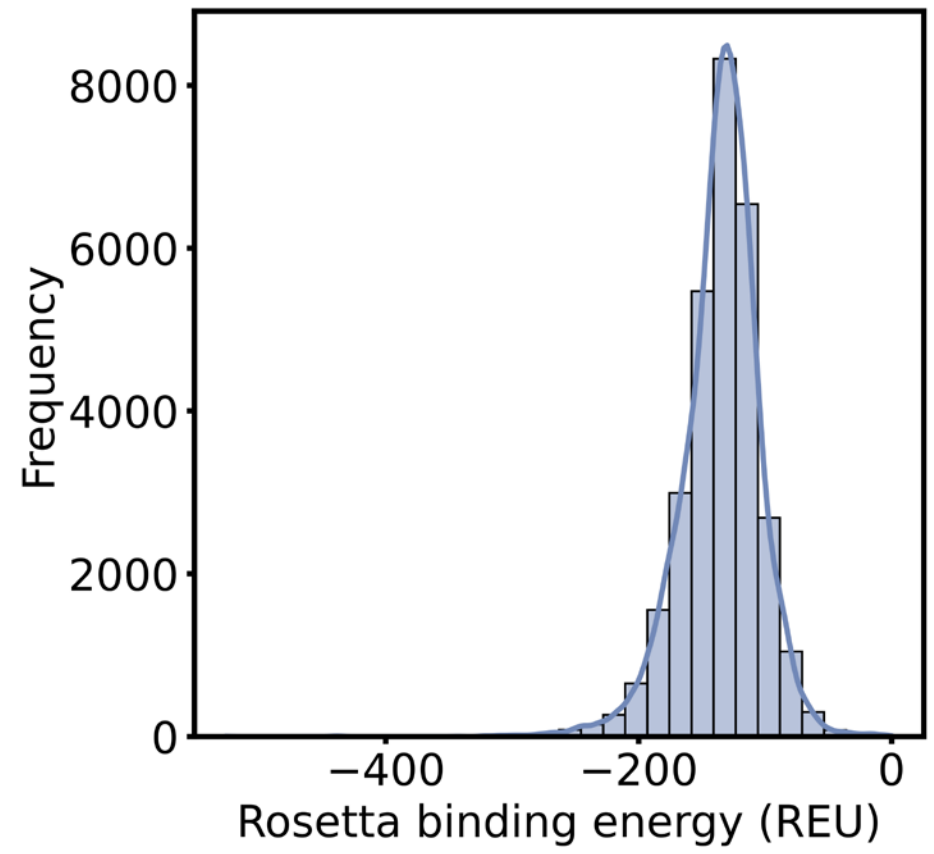

Supplementary Fig. 9. Rosetta binding energy distribution of training sequences (n = 79,890 sequences).

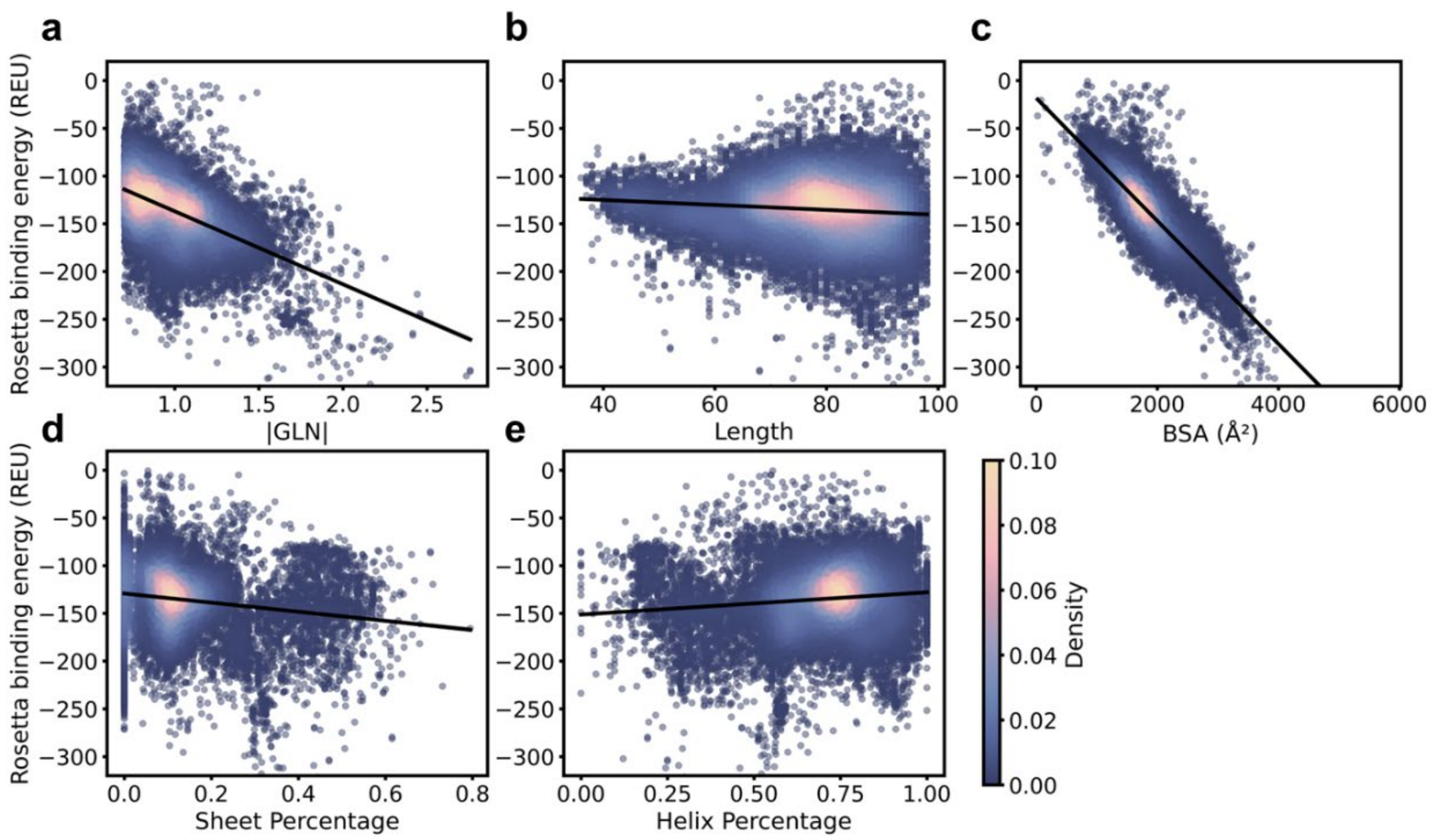

Supplementary Fig. 10. Correlation between Rosetta binding energy and (a) the absolute value of GLN (|GLN|), (b) sequence length, (c) buried surface area (BSA), (d) percentage of beta sheets, and (e) percentage of alpha helices. Binding energies were computed using Rosetta following structure relaxations. Data points represent individual training sequences (n = 79,890 sequences). The solid lines indicate linear fits, and the shaded bands indicate 95% confidence intervals estimated using 1,000 bootstrap resamples. No null-hypothesis significance tests were performed.

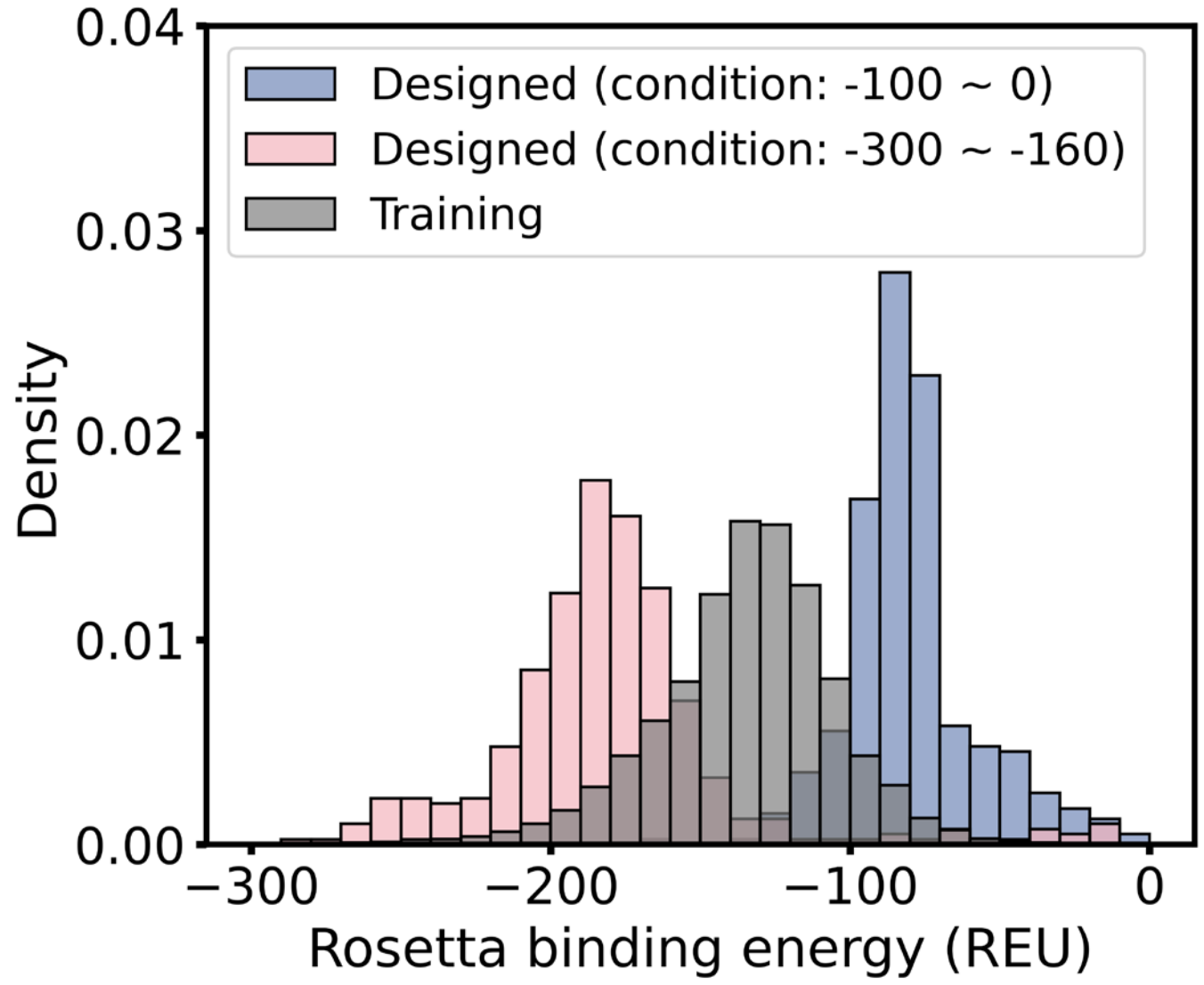

Supplementary Fig. 11. Binding energy distribution of 400 designed sequences generated with condition -100 to 0 REU, 400 designed sequences generated with condition -300 to -160 REU, and 79,890 training sequences.

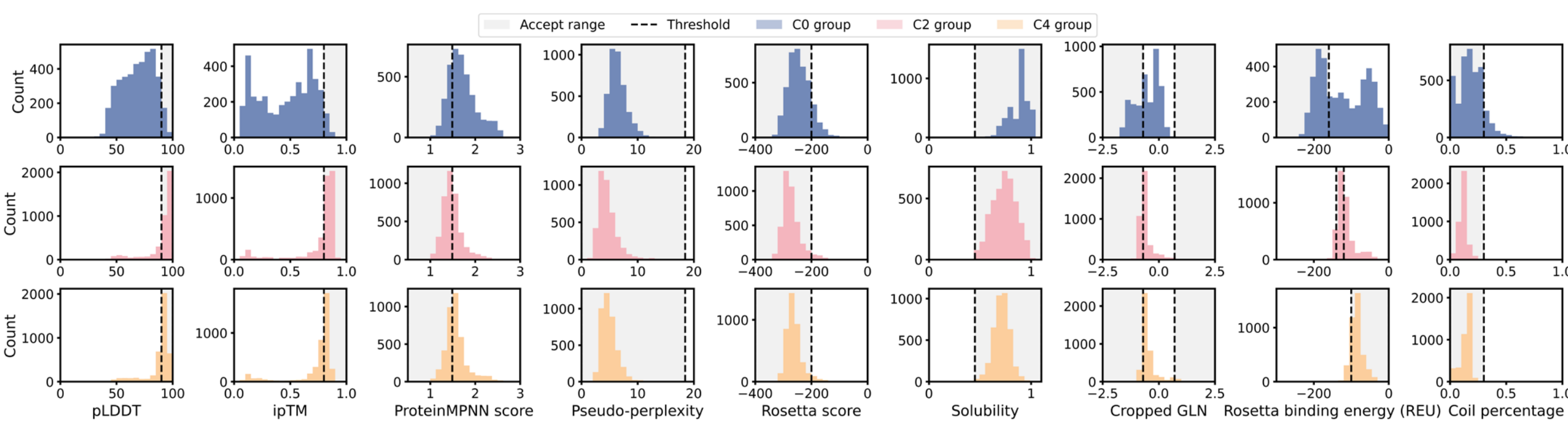

Supplementary Fig. 12. Distribution of various metrics employed during virtual screening for conditionally generated designs. Colours identify the different binding-energy condition groups, as indicated in the in-panel key. Shaded regions indicate the ranges containing the designs selected for experimental validation.

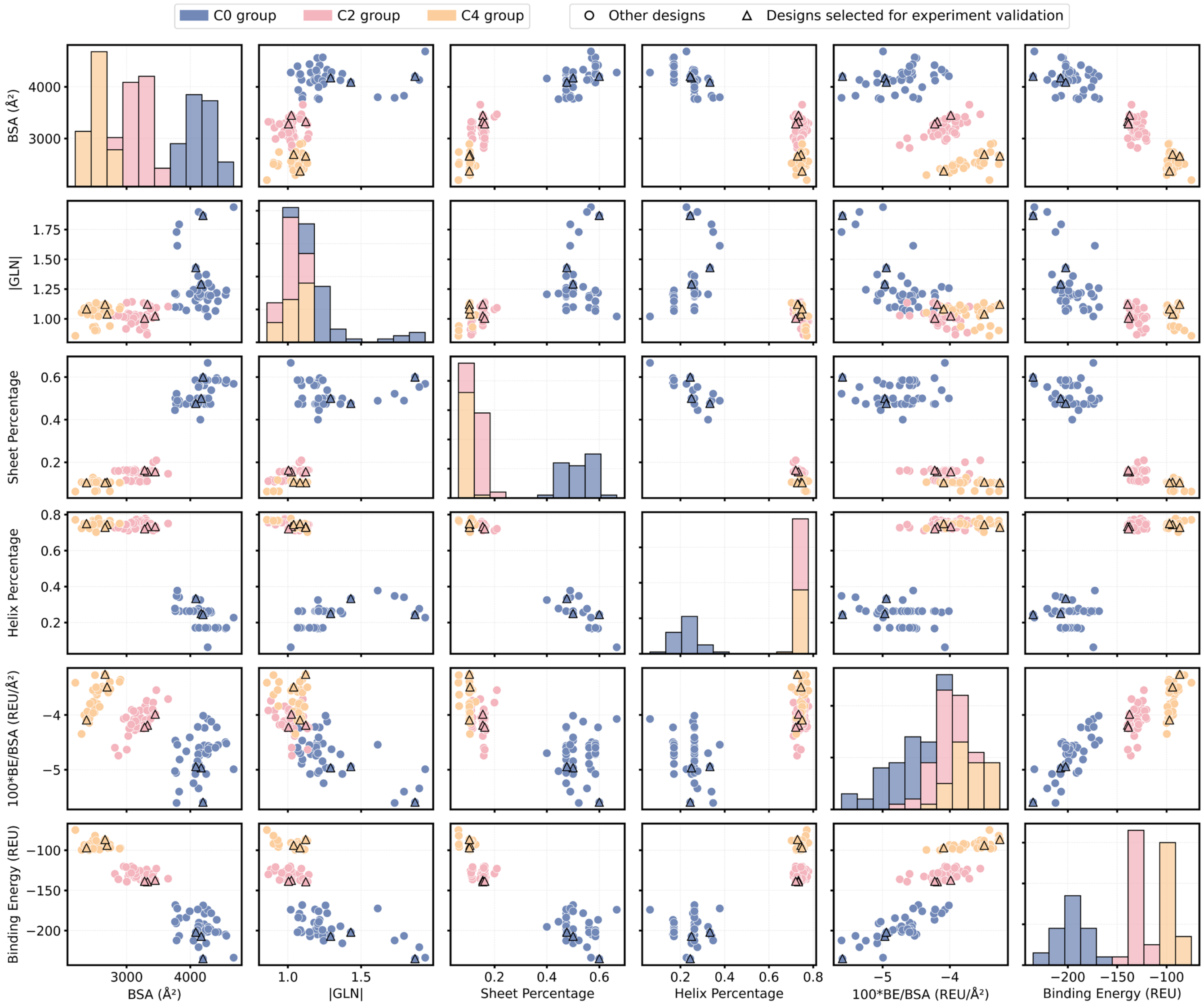

Supplementary Fig. 13. Correlation between various structural features of conditionally generated sequences following filtration through the virtual screening pipeline. The quantity 100*BE/BSA denotes 100 times the Rosetta binding energy divided by the buried surface area (BSA). Designs selected for experimental validation are indicated with triangles. (n = 561 designs)

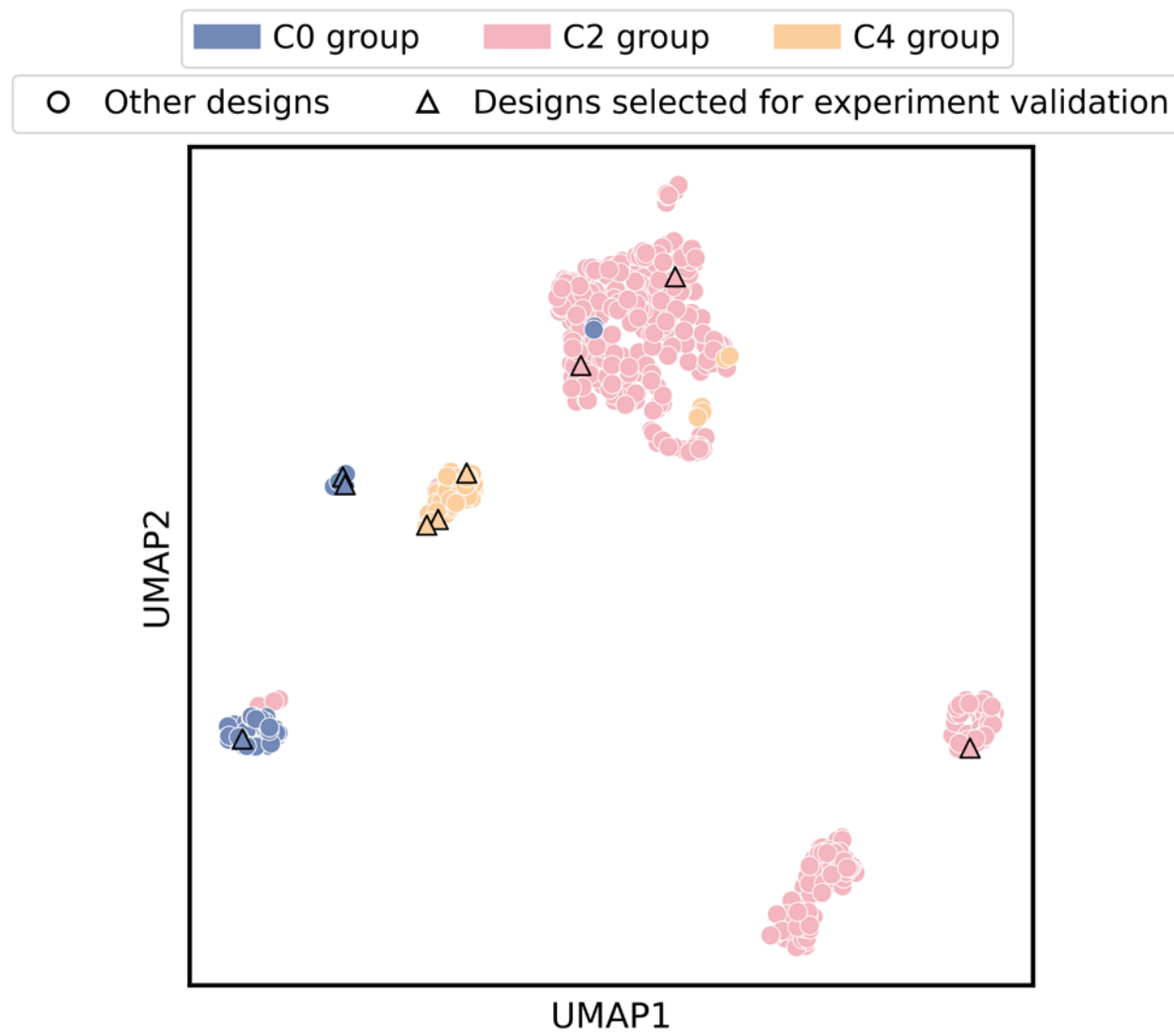

Supplementary Fig. 14. Conditionally generated sequences from distinct binding energy groups are embedded and projected into a two-dimensional space. Each sequence was embedded using ESM2 (650M) to create a token-level representation with dimensions of (Length, 1280). This representation was then averaged over the length dimension, yielding a sequence-level representation with dimensions of (1280,). These sequence-level representations were subsequently projected into two dimensions using Uniform Manifold Approximation and Projection (UMAP). Designs selected for experimental validation are marked with triangles. (n = 561 designs)

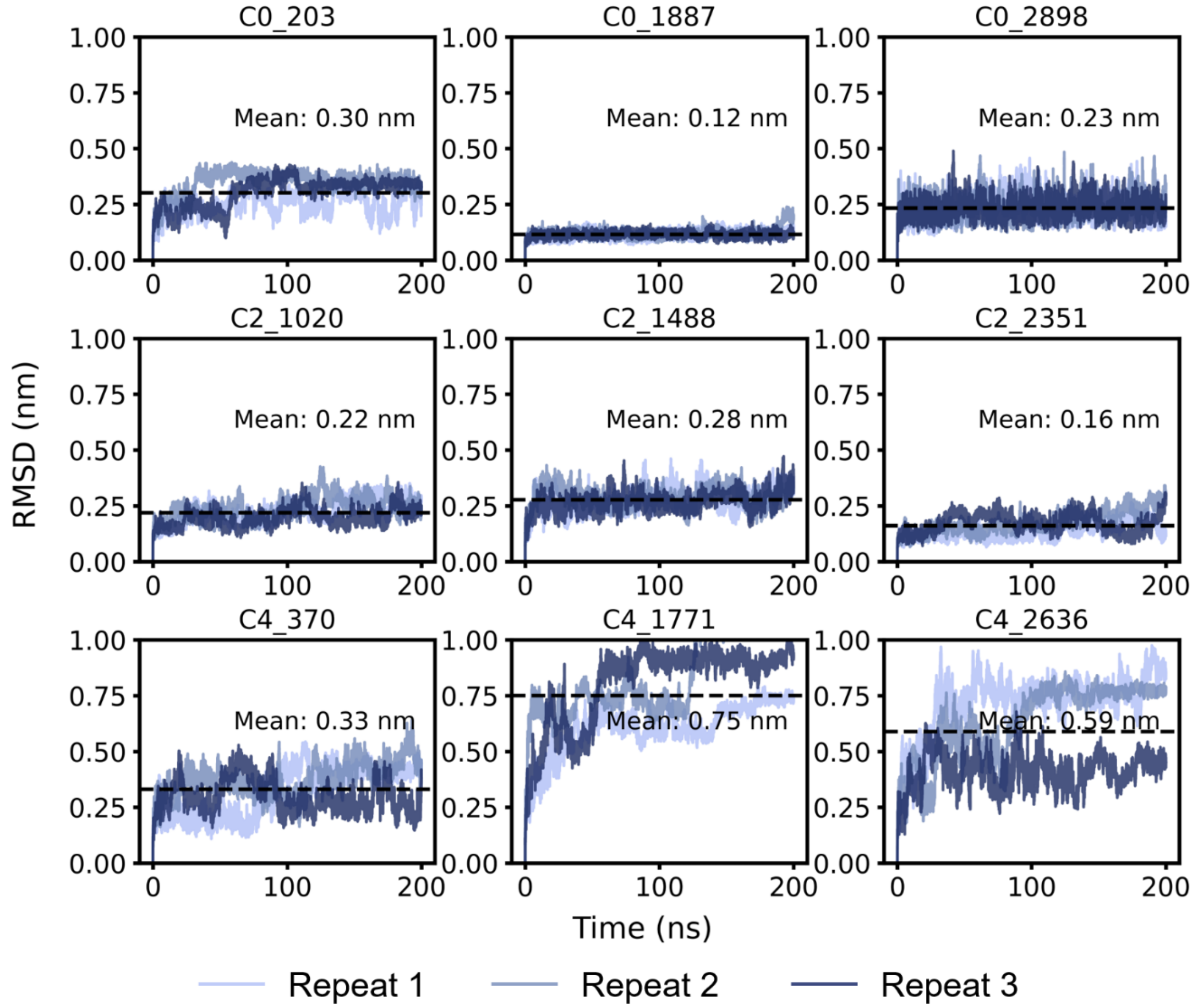

Supplementary Fig. 15. Root-mean-square deviation (RMSD) versus molecular simulation time for nine experimentally validated designs. In the design IDs, C0 indicates the strongest binding energy, while C4 represents the weakest. Designs with higher binding energy tend to exhibit less structural deviation and fluctuation during simulation, indicating greater stability. Each curve represents one 200-ns molecular-dynamics simulation of an individual design (n = 9 designs, with three independent simulation replicates per design).

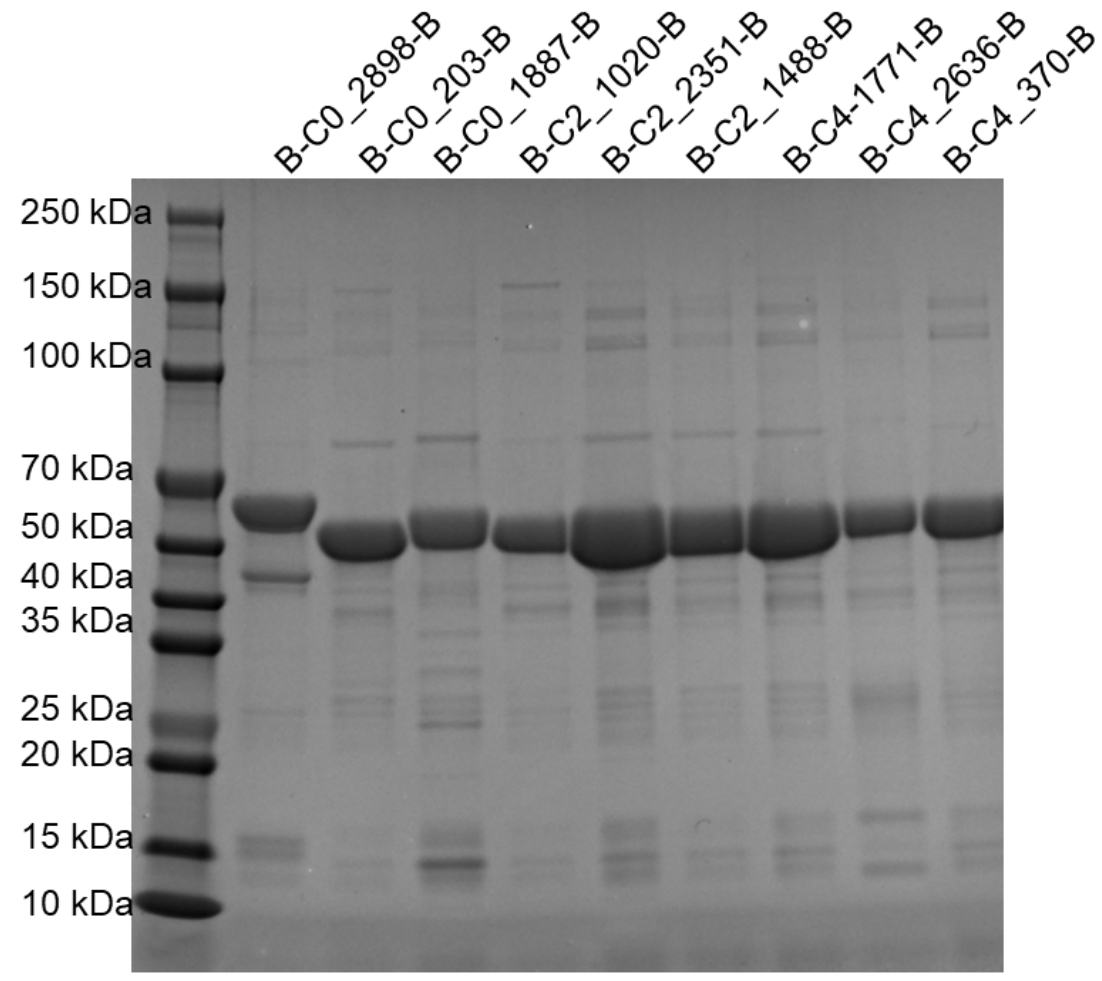

Supplementary Fig. 16. SDS-PAGE of nine B-X-B designs. The experiment was independently repeated 3 times with similar results.

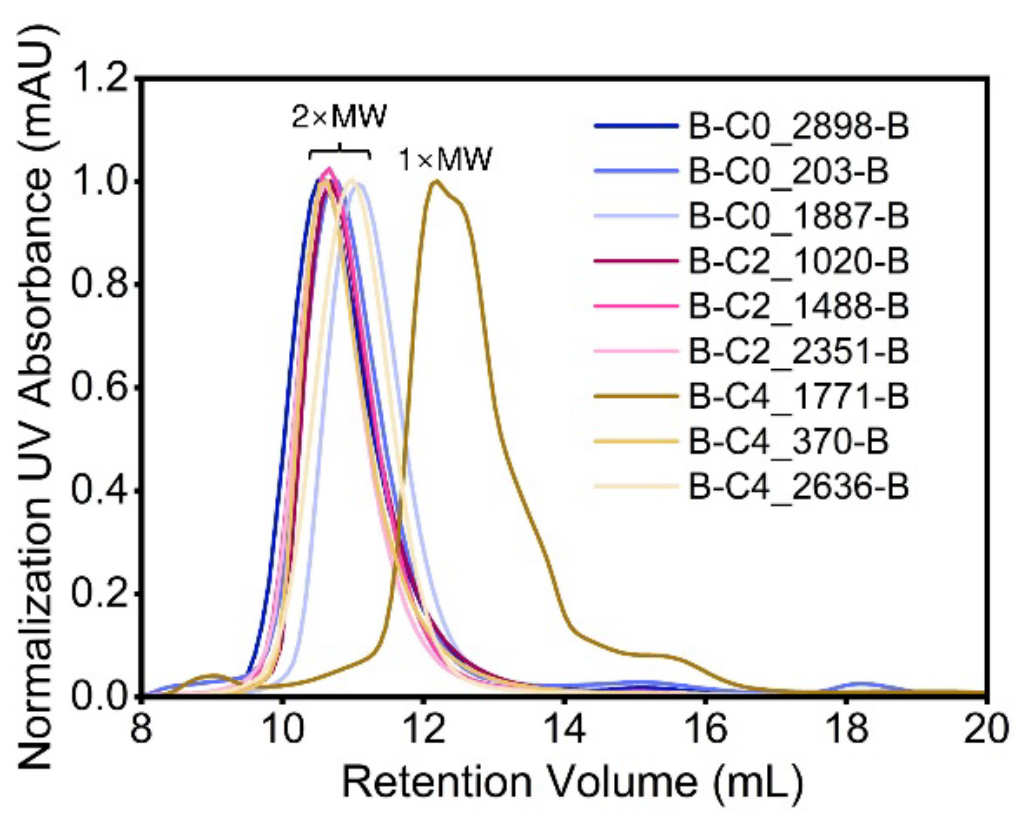

Supplementary Fig. 17. SEC-MALS measurement of B-X-B. B-C4_1771-B is monomeric in solution, whereas the other constructs are homodimeric, consistent with the SEC-MALS results for the corresponding A-X-A constructs shown in Figure 5d. MW refers to molecular weight. Measurements were independently repeated twice with similar results.

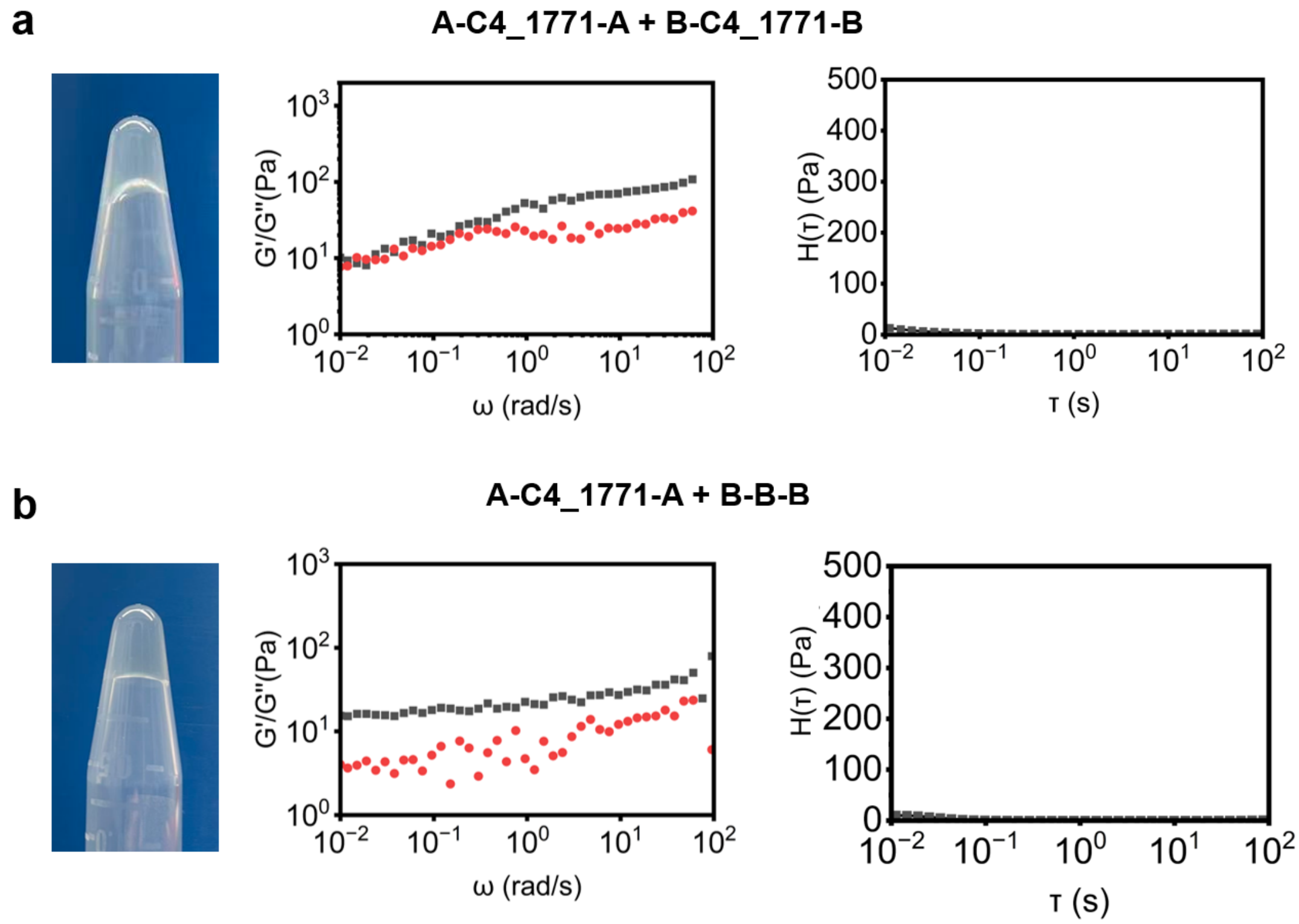

Supplementary Fig. 18. Protein networks prepared using the C4_1771 design. (a) A-X-A + B-X-B and (b) A-X-A + B-B-B networks prepared using C4_1771. Left, photographs of the protein mixtures; middle, dynamic mechanical analysis; right, continuous relaxation spectra. C4_1771 was monomeric and served as a negative control for the dimeric designs. Unlike the other designs, which formed gels, both the A-C4_1771-A + B-C4_1771-B and A-C4_1771-A + B-B-B mixtures remained viscous liquids and gradually flowed down the vial wall. Neither network showed continuous frequency-sweep curves or detectable relaxation peaks. Their moduli were substantially lower than those of networks prepared using the dimeric designs. Hydrogel preparation and rheological tests were independently repeated three times with similar results.

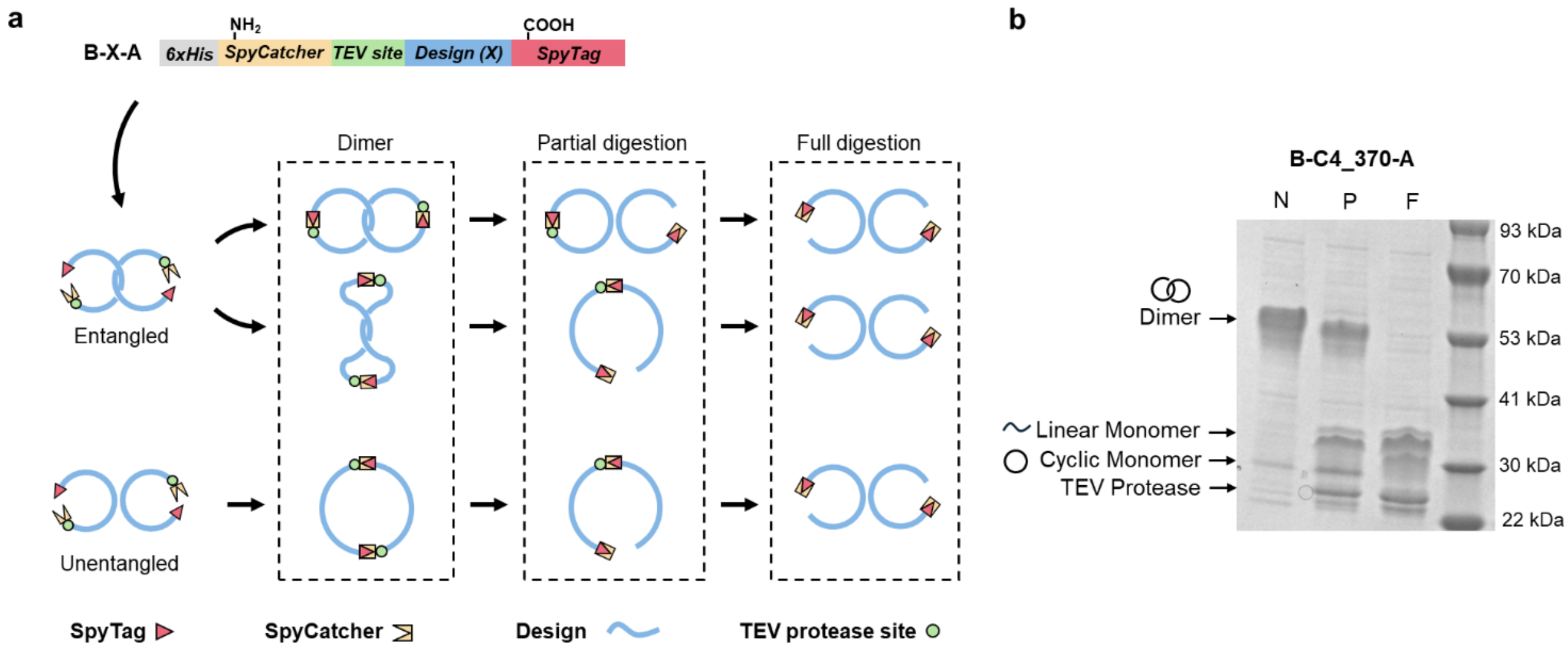

Supplementary Fig. 19. Experimental validation of structural entanglement in the designs. (a) Schematic of the mechanically interlocked protein construct used to test structural entanglement. B-X-A constructs were prepared using the gene cassette previously applied to catenane synthesis.[4,5] The dimeric products were purified and subjected to tobacco etch virus (TEV) protease-mediated cleavage. Purified, partially digested and fully digested products were analysed by SDS-PAGE. The formation of cyclic monomers after digestion supports the presence of catenanes in the dimeric products and, consequently, structural entanglement in the designs before covalent ligation. (b) SDS-PAGE analysis of the TEV protease digestion products of B-C4_370-A. N, no digestion; P, partial digestion; F, full digestion. The experiment was independently repeated twice with similar results.

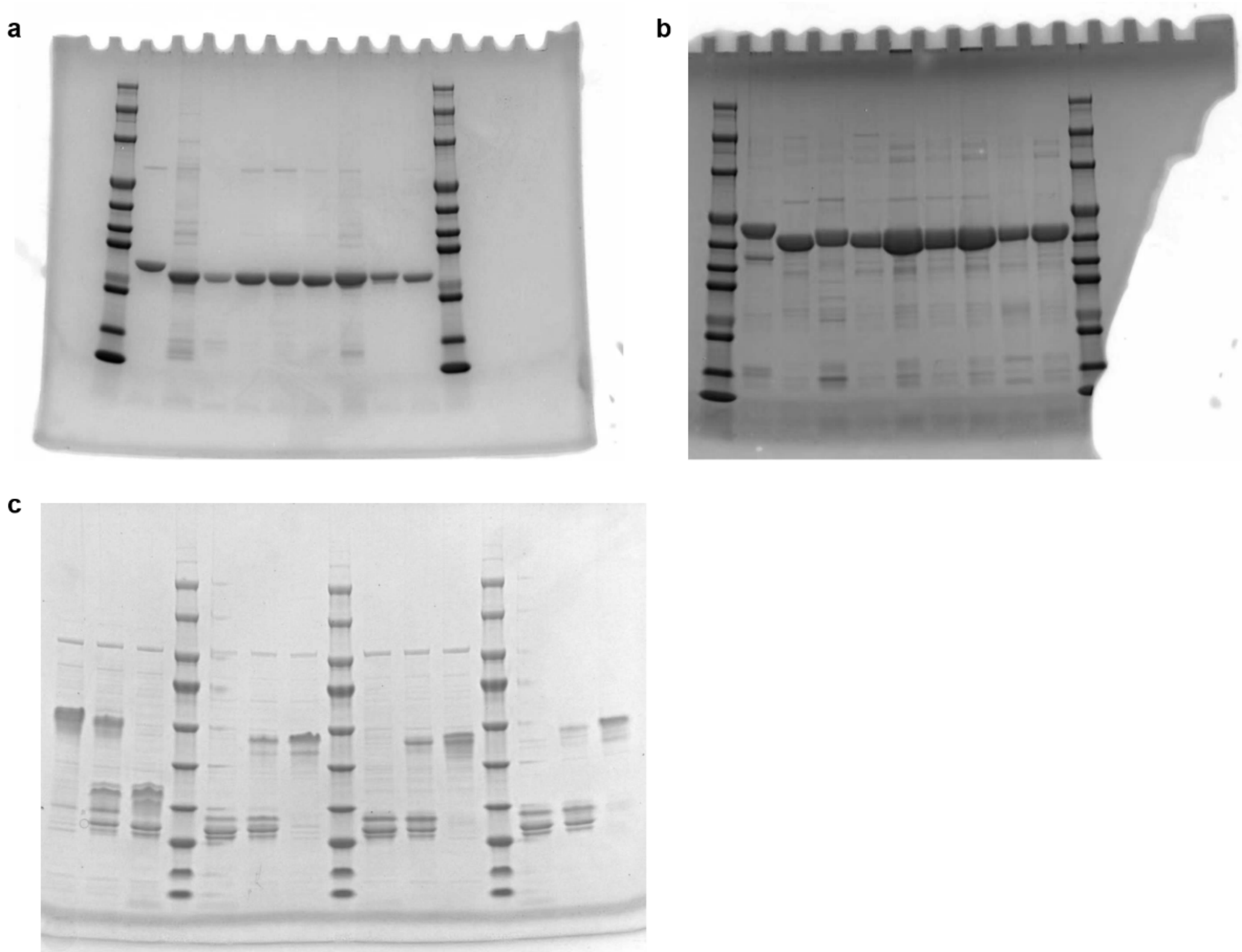

Supplementary Fig. 20. Source data of SDS-PAGE in (a) Figure 5b; (b) Supplementary Fig. 16; (c) Supplementary Fig. 19b.

Supplementary Tables

Supplementary Table 1. Virtual screening steps of generated sequences under different Rosetta binding energy conditions. BE refers to the binding energy given by Rosetta.

**Filtering criteria**		**Length 40 – 50<br>BE: < -160**	**Length 40 – 50<br>BE: -140~ -120**	**Length 40 – 50<br>BE: -100 ~ 0**
generated		4000	4000	4000
Structure-sequence pair reliability	AF2 pLDDT ≥ 90<br>ipTM ≥ 0.8	140	2801	2069
	ProteinMPNN score <1.4906	112	1886	992
Stability/solubility	Rosetta score < -200<br>Single chain Rosetta score < -65	111	1886	989
	ProteinSol solubility ≥ 0.45	111	1883	985
	No Cysteine	102	1684	930
Geometries	Cropped |GLN| ≥ 0.7<br>Coil percentage < 0.3	54	746	130
Binding energy condition		54	458	49

Supplementary Table 2. Detailed metrics of nine designs for experiments.

Design ID	pLDDT	ipTM	GLN	Length	Rosetta energy	Rosetta binding energy	gmx_MMPBSA binding energy (kcal/mol)
C0_2898	92.14	0.80	-1.87	49	-318.4	-235.1	-280.3
C0_203	93.42	0.84	-1.29	46	-298.1	-207.2	-260.2
C0_1887	96.60	0.86	-1.43	45	-286.1	-202.1	-213.5
C2_1020	90.09	0.82	-1.02	47	-305.9	-137.5	-162.9
C2_2351	98.18	0.90	-1.13	45	-318.0	-138.7	-137.9
C2_1488	96.34	0.85	-1.12	47	-298.1	-139.3	-133.7
C4_1771	93.64	0.80	-1.12	50	-294.7	-86.7	-100.9

C4_2636	97.33	0.87	-1.08	50	-298.3	-97.0	-95.4
C4_370	97.24	0.87	-1.04	49	-285.1	-94.0	-89.3

Design ID	BSA (Å^2)	100*BE/BSA (REU/Å^2)	Protein-Sol solubility	Intra-chain terminal distance A (Å)	Intra-chain terminal distance A (Å)	Inter-chain terminal distance (N-N termini, Å)	Intra-chain terminal distance (C-C termini, Å)
C0_2898	4200.7	-5.60	0.881	65.4	65.4	57.3	73.6
C0_203	4172.2	-4.96	0.907	39.6	39.6	39.3	32.7
C0_1887	4087.8	-4.94	0.885	35.4	35.4	35.9	31.1
C2_1020	3450.2	-3.99	0.842	26.6	26.6	24.7	25.4
C2_2351	3283.3	-4.22	0.772	27.0	27.0	24.6	23.4
C2_1488	3327.9	-4.19	0.633	34.4	34.4	24.6	50.2
C4_1771	2662.0	-3.25	0.581	43.7	43.7	19.0	34.0
C4_2636	2371.1	-4.09	0.797	44.0	44.0	19.5	27.6
C4_370	2693.9	-3.49	0.669	42.7	42.7	19.2	24.5

Supplementary Table 3. Corresponding molecular weights of chromatographic peaks measured by SEC-MALS.

	Measured MW (kDa)	Theoretical MW (kDa)
A- C0_1887-A	44.0 (± 2.8 %)	24.2
A- C0_203-A	58.8 (± 2.2 %)	24.5
A- C2_1020-A	48.3 (± 1.4 %)	24.7
A- C2_2351-A	51.5 (± 2.3 %)	24.6
A- C0_2898-A	52.1 (± 1.5 %)	25.0
A- C4_370-A	48.6 (± 1.6 %)	24.9
A- C2_1488-A	52.3 (± 3.7 %)	24.6

A- C4_1771-A	28.9 (± 3.7 %)	24.8
A- C4_2636-A	51.8 (± 2.8 %)	24.95
B- C0_1887-B	94.0 (± 2.1 %)	51.0
B- C0_203-B	107.8 (± 1.4 %)	50.7
B- C2_1020-B	88.8 (± 1.2 %)	50.8
B- C2_2351-B	105.4 (± 1.1 %)	50.8
B- C0_2898-B	95.8 (± 1.4 %)	51.2
B- C4_370-B	92.5 (± 1.6 %)	51.0
B- C2_1488-B	102.2 (± 1.5 %)	50.8
B- C4_1771-B	58.9 (± 3.7 %)	50.95
B- C4_2636-B	87.3 (± 1.4 %)	51.1

Supplementary Table 4. Parameters of molecular simulations.

System ID	Box Dimensions (nm)	Total Atoms	Water Molecules	Ions Added	Salt Concentration (mM)
C0_2898	9.806 x 9.806 x 9.806	93,247	30,541	16 x NA^+	281.7
C0_203	6.537 x 6.537 x 6.537	27,302	8,580	4 x CL^-	237.7
C0_1887	5.993 x 5.993 x 5.993	21,089	6,569	18 x NA^+	1,388.2
C2_1020	5.995 x 5.995 x 5.995	21,197	6,551	2 x CL^-	154.1
C2_2351	7.123 x 7.123 x 7.123 (cubic)	35,493	11,291	4 x CL^-	~142
C2_1488	7.107 x 7.107 x 7.107 (cubic)	35,254	11,212	2 x NA^+	~72

System ID	Box Dimensions (nm)	Total Atoms	Water Molecules	Ions Added	Salt Concentration (mM)
C4_2636	7.201 x 7.201 x 7.201	36,544	11,654	4 x NA^+	177.9
C4_370	7.092 x 7.092 x 7.092	35,078	11,164	None	0
C4_1771	7.035 x 7.035 x 7.035	34,189	10,867	2 x CL^-	95.4

Supplementary References

1. Ho, J., Jain, A. & Abbeel, P. Denoising diffusion probabilistic models. *In Advances in Neural Information Processing Systems* **33**, 6840–6851 (2020).
2. Song, J., Meng, C. & Ermon, S. Denoising diffusion implicit models. *In International Conference on Learning Representations* (2021).
3. Chen, T., Zhang, R. & Hinton, G. Analog bits: generating discrete data using diffusion models with self-conditioning. *In International Conference on Learning Representations* (2023).
4. Wu, W.-H., Guo, J., Zhang, L., Zhang, W.-B. & Gao, W. Peptide/protein-based macrocycles: from biological synthesis to biomedical applications. *RSC Chem. Biol*. **3**, 815–829 (2022).
5. Liu, D. et al. Topology engineering of proteins in vivo using genetically encoded, mechanically interlocking SpyX modules for enhanced stability. *ACS Cent. Sci*. **3**, 473–481 (2017).
6. Steinegger, M. & Söding, J. MMseqs2 enables sensitive protein sequence searching for the analysis of massive data sets. *Nat. Biotechnol*. **35**, 1026–1028 (2017).
7. Van Kempen, M. et al. Fast and accurate protein structure search with Foldseek. *Nat. Biotechnol*. **42**, 243–246 (2024).
8. Alamdari, S. et al. Protein generation with evolutionary diffusion: sequence is all you need. *NeurIPS 2023 Generative AI and Biology Workshop* (2023). https://openreview.net/forum?id=qP69kXPdJM.
9. Nijkamp, E., Ruffolo, J. A., Weinstein, E. N., Naik, N. & Madani, A. ProGen2: exploring the boundaries of protein language models. *Cell Syst*. **14**, 968–978.e3 (2023).
