# Supplementary material for: Generating protein hydrogels with customizable stress relaxation behavior via deep learning-driven entanglement design
Source: Nat Commun. 2026 Sep 21;17:11006. doi: 10.1038/s41467-026-77607-9 (PMC13687093; doi:10.1038/s41467-026-77607-9)
Supplement: Supplementary file 2 [file 41467_2026_77607_MOESM2_ESM.pdf]

Corresponding author(s): Hanyu Gao

Last updated by author(s): Aug 24, 2026

Reporting Summary

Nature Portfolio wishes to improve the reproducibility of the work that we publish. This form provides structure for consistency and transparency in reporting. For further information on Nature Portfolio policies, see our Editorial Policies and the Editorial Policy Checklist.

Statistics

For all statistical analyses, confirm that the following items are present in the figure legend, table legend, main text, or Methods section.

n/a	Confirmed	
☐	☒	The exact sample size (*n*) for each experimental group/condition, given as a discrete number and unit of measurement
☐	☒	A statement on whether measurements were taken from distinct samples or whether the same sample was measured repeatedly
☒	☐	The statistical test(s) used AND whether they are one- or two-sided<br>*Only common tests should be described solely by name; describe more complex techniques in the Methods section.*
☐	☒	A description of all covariates tested
☐	☒	A description of any assumptions or corrections, such as tests of normality and adjustment for multiple comparisons
☐	☒	A full description of the statistical parameters including central tendency (e.g. means) or other basic estimates (e.g. regression coefficient) AND variation (e.g. standard deviation) or associated estimates of uncertainty (e.g. confidence intervals)
☒	☐	For null hypothesis testing, the test statistic (e.g. *F*, *t*, *r*) with confidence intervals, effect sizes, degrees of freedom and *P* value noted<br>*Give P values as exact values whenever suitable.*
☐	☒	For Bayesian analysis, information on the choice of priors and Markov chain Monte Carlo settings
☐	☒	For hierarchical and complex designs, identification of the appropriate level for tests and full reporting of outcomes
☒	☐	Estimates of effect sizes (e.g. Cohen's *d*, Pearson's *r*), indicating how they were calculated

Our web collection on statistics for biologists contains articles on many of the points above.

Software and code

Policy information about availability of computer code

Data collection	Custom code was used to collect the data. (https://github.com/daniel-dpq/TangleDiff.git / https://doi.org/10.6084/m9.figshare.32658021)
Data analysis	Foldseek (8979d230fb64c7089380b652758d8705493ed4a5), MMseqs2(13-45111+ds-2), ESMFold(v2023_02), AlphaFold(v2.3.0), matplotlib(3.5.3), OriginLab(2026b), GROMACS (2024.3), BLASTp(2.12.0+), Rosetta(2024.09+release.06b3cf8), gmx_MMPBSA(1.6.4), MDTraj(1.9.9), ProteinMPNN(v1.0.1), Biopython(1.79)

For manuscripts utilizing custom algorithms or software that are central to the research but not yet described in published literature, software must be made available to editors and reviewers. We strongly encourage code deposition in a community repository (e.g. GitHub). See the Nature Portfolio guidelines for submitting code & software for further information.

Data

Policy information about availability of data

All manuscripts must include a data availability statement. This statement should provide the following information, where applicable:

- Accession codes, unique identifiers, or web links for publicly available datasets
- A description of any restrictions on data availability
- For clinical datasets or third party data, please ensure that the statement adheres to our policy

The UniRef50 clusters are available at https://www.uniprot.org/. Training sequences with their calculated features are available at https://github.com/daniel-dpq/TangleDiff.git. The ESMFold-predicted homodimeric structures of training sequences (with their random coil terminals cropped) are available at FigShare (https://

doi.org/10.6084/m9.figshare.29604998.v1). Source data are provided with this paper. Raw data used for all figures/tables are also available at https://doi.org/10.6084/m9.figshare.32658021. Raw data of SDS-PAGE are available in the supplementary file.

Research involving human participants, their data, or biological material

Policy information about studies with human participants or human data. See also policy information about sex, gender (identity/presentation), and sexual orientation and race, ethnicity and racism.

Reporting on sex and gender	n/a
Reporting on race, ethnicity, or other socially relevant groupings	n/a
Population characteristics	n/a
Recruitment	n/a
Ethics oversight	n/a

Note that full information on the approval of the study protocol must also be provided in the manuscript.

Field-specific reporting

Please select the one below that is the best fit for your research. If you are not sure, read the appropriate sections before making your selection.

☒ Life sciences ☐ Behavioural & social sciences ☐ Ecological, evolutionary & environmental sciences

For a reference copy of the document with all sections, see nature.com/documents/nr-reporting-summary-flat.pdf

Life sciences study design

All studies must disclose on these points even when the disclosure is negative.

Sample size	No statistical method was used to predetermine sample size. Computational sample sizes were selected to provide sufficiently large datasets for model training and evaluation. For conditional generation, 400 sequences were generated for each binding-energy condition in Fig. 4b–c, and 4,000 sequences were generated for each condition for virtual screening. For unconditional-generation evaluation, 4,000 designed sequences were analysed. Nine designs spanning the predefined binding-energy groups were selected for experimental validation. Experimental replicate numbers are reported in the corresponding figure legends and in the Statistics and reproducibility subsection.
Data exclusions	No data were excluded from the analysis
Replication	The SDS-PAGE experiments shown in Fig. 5b and Supplementary Fig. 16 were independently repeated three times with similar results. CD spectra were obtained by averaging three consecutive scans of the same sample; these scans were repeated technical measurements rather than independent biological replicates. Hydrogel preparation and rheological tests were independently repeated three times with similar results. Independent-repeat information for the other experimental measurements is provided in the corresponding figure legends and in the Statistics and reproducibility subsection. All attempted independent replications were successful, unless otherwise stated.
Randomization	Randomized allocation to experimental treatment groups was not applicable because the study did not involve treatment-group comparisons. The nine protein designs were randomly selected from predefined computational binding-energy groups as described in the Methods.
Blinding	Blinding was not applicable because the study did not involve allocation to experimental treatment groups, and the identities of the protein designs were required during protein production and characterization.

Reporting for specific materials, systems and methods

We require information from authors about some types of materials, experimental systems and methods used in many studies. Here, indicate whether each material, system or method listed is relevant to your study. If you are not sure if a list item applies to your research, read the appropriate section before selecting a response.

Materials & experimental systems

n/a	Involved in the study
☒	☐ Antibodies
☒	☐ Eukaryotic cell lines
☒	☐ Palaeontology and archaeology
☒	☐ Animals and other organisms
☒	☐ Clinical data
☒	☐ Dual use research of concern
☒	☐ Plants

Methods

n/a	Involved in the study
☒	☐ ChIP-seq
☒	☐ Flow cytometry
☒	☐ MRI-based neuroimaging

Plants

Seed stocks

n/a

Novel plant genotypes

n/a

Authentication

n/a
