# Supplementary material for: Generating protein hydrogels with customizable stress relaxation behavior via deep learning-driven entanglement design
Source: Nat Commun. 2026 Sep 21;17:11006. doi: 10.1038/s41467-026-77607-9 (PMC13687093; doi:10.1038/s41467-026-77607-9)
Supplement: Supplementary file 3 [file 41467_2026_77607_MOESM3_ESM.pdf]

nature portfolio

Peer Review File

Generating Protein Hydrogels with Customizable Stress Relaxation Behavior via Deep Learning-Driven Entanglement Design

Corresponding Author: Professor Hanyu Gao

This file contains all reviewer reports in order by version, followed by all author rebuttals in order by version.

Version 0:

Reviewer comments:

Reviewer #1

(Remarks to the Author)
Overall comments

The authors present an impressive and well-executed study that integrates pretrained protein language models with physically grounded molecular interaction constraints to enable conditional de novo design of entangled protein complexes with programmable binding strengths and topological features. The computational framework is elegant, and the experimental validation—particularly the successful expression, assembly, and rheological characterization of the designed proteins—provides compelling evidence for the effectiveness of the approach.

The work convincingly demonstrates that pretrained protein language models can function as powerful latent encoders for conditional protein generation when combined with physically meaningful constraints, bridging molecular-level design and macroscopic material properties. Overall, this is a high-quality contribution with clear conceptual novelty and strong experimental support.

Specific comments

(1) Figures 1f and 1g primarily provide taxonomic and functional distribution statistics of the curated dataset. While informative, they are not essential for supporting the central methodological and conceptual advances of the manuscript. Consider moving these panels to the Supplementary Information to improve the focus and pacing of the main text.

(2) In line 137, the word “nosing” in the phrase “trained to align the denoising and nosing process” should be corrected to “noising.”

(3) In line 159, the capitalization of adaLN-zero is inconsistent with other occurrences in the manuscript (e.g., AdaLN-Zero). Please ensure consistent capitalization and nomenclature throughout the text and figures.

(4) In Figure 3a and the associated text, the structure prediction pipeline should be described more explicitly as involving both ESMFold and AlphaFold2, rather than being presented as image-based results. Clarifying how predictions from the two models are used (e.g., comparison, validation, or filtering) would improve methodological transparency.

(5) The Molecular Dynamics section in the Methods requires further clarification and correction. In particular, the reported simulation box length of 1 nm is unrealistically small for a protein homodimer system and would not yield physically meaningful results. The authors should clarify whether this value is a typographical error. If so, the correct box dimensions should be reported. Otherwise, the simulations should be repeated using an appropriately sized solvation box, and the revised setup and results should be described.

(6) The manuscript relies heavily on the absolute Gauss Linking Number (GLN) as the primary metric for identifying and quantifying entanglement. While GLN is well motivated, it would strengthen the work to briefly discuss its robustness with respect to structural uncertainty (e.g., prediction noise or thermal fluctuations), or to justify the chosen threshold (|GLN| ≥ 0.7)

more explicitly, possibly with references or sensitivity analysis.

(7) The authors note that the conditioning framework can, in principle, be extended to other molecular-level properties. Including a brief discussion or example (even speculative) of additional conditioning targets—such as entanglement depth, secondary-structure composition, or kinetic stability—would further highlight the generality and future potential of the approach.

(8) Given the complexity of the computational pipeline, it would be beneficial to clearly indicate which components (trained models, datasets, scripts) are publicly available and which are not, and to provide guidance on how a reader might reproduce or adapt the workflow for related protein design problems.

(Remarks on code availability)
I didn't run the code by myself. I am not able to assess the code as I am not an expert in this field.

Reviewer #2

(Remarks to the Author)
In this manuscript, the authors made a study on the computational–experimental framework of the design of entangled proteins for application in the stress-relaxing protein hydrogels. The integration of large-scale sequence mining, generative modeling, and materials characterization is fine, and the work is of potential interest to a broad audience. However, some design methodological issues and computational details, and realization processes of the entanglement-crosslinked protein hydrogels are not well described in its current form of the manuscript. The authors should make a major revision on additional analyses, clarification, and discussions as listed in the following.

1.Only nine designs which were selected after multiple rounds of virtual screening and random sampling (three per binding-energy group) were experimentally tested. The authors did not report overall failure rates or the distribution of discarded candidates, making it difficult to assess representativeness and to exclude success-story bias.

2.The candidate pool sizes differ substantially across groups (~54, 458, and 49). Randomly selecting three designs per group may not adequately capture structural or sequence diversity, and the rationale for this sample size is not justified.

3. The MD simulations may be too short to argue the structural stability. For the entangled proteins, the key challenge may be a problem of the kinetic folding accessibility. The current evidences do not clearly distinguish between the structures that are locally stable and those that can realistically fold into the entangled topology.

4.The hydrogel networks certainly contain multiple potential relaxation mechanisms (entanglement, SpyTag/SpyCatcher crosslinks, ELP chain dynamics). It remains unclear how non-entanglement-related contributions to the observed relaxation spectra are excluded. This should be discussed well.

5.The authors did not analyze whether inter-chain binding energy is correlated with other structural descriptors (e.g., |GLN|, interface area, secondary structure content). Without such analysis, the independence of conditional control may be not established.

6.The reported success rate depends on specific threshold choices (say, the pLDDT > 80, and $|GLN| \geq 0.7$). Clearly, no sensitivity analysis is provided, and this metric reflects internal pass rates rather than predictive accuracy. Is there a better way to estimate the prediction accuracy?

(Remarks on code availability)

Reviewer #3

(Remarks to the Author)
In this work, the authors focus on introducing entanglement into proteins for tuning the mechanical properties. The de novo design of entangled proteins has been achieved with a deep learning-based model of natural sequences with potential entanglement and diffusion-based generative model. This work achieved the design and production of hydrogel macroscopic protein materials via conditional protein design. However, there are limitations of the model building and critical experimental evidence are missing to support several conclusions in this work. I would like the following comments to be addressed.

The current approach to entangled homodimer dataset construction faces a fundamental challenge: static folding predictions often deviate from experimental reality, particularly for complex topologies that are kinetically difficult to form. Furthermore, the dataset may be biased toward proteins that resemble existing natural folds, limiting diversity. While the current work will be difficult for this major revision, I have suggestions for future work dataset construction.
The limitation in structural diversity can be mitigated by clustering the UniProt source data at a low identity threshold (e.g., 30%). This ensures the model learns the underlying physics of chain intertwining rather than evolutionary redundancy.
To minimize the gap between computation and lab results, I suggest implementing high-recycling runs in AlphaFold 2 or 3

alongside ESMfold. Using iPAE as a filtering metric—specifically for these oligomeric interfaces—would provide higher confidence in the topological validity of the designs before moving to experimental validation.
That being said, I would like to see a metric added to the SI showing sequence identity distribution of the 79890 sequences that the authors used for this current work.

In the Results section of “unconditional design of entangled homodimers” (line 177), the authors claim that the designed sequences show low sequence identity to the training dataset. However, Figure S3b shows a sequence identity distribution centered at ~50% with significant variance (large error bars). In the context of de novo design, 50% identity often represents a level of homology where structural and even functional conservation is expected. Please revise this statement and provide a histogram of the identities rather than just a mean/error bar.

In this work, the authors attempted to design entangled homodimer proteins and several statements in the main text indicate that entangled homodimers form in the designs (eg. line 212 “TangleDiff can design functional entangled homodimers”). However, critical structural evidence shows entangled homodimers. Please try TEM or HRTEM characterization or X-ray crystallography on at least one of the designs. The AF2 predictions in Fig3 can not be used as experimental evidence and SEC only suggests dimer formation but not entanglement or assembly.

In the results section using Rosetta to calculate the binding energy, line 279, the authors used sequence lengths from 40-50 amino acids. A 40AA protein is kinetically favorable for entanglement folding but such a short sequence usually doesn't have enough surface area to achieve that binding energy score without being "over-packed" or having massive hydrophobic exposure. Since entangled structures naturally bury more surface area than standard dimers, the reported REU values are likely a proxy for the extent of entanglement rather than the specific quality of the interface. To validate that the group (< -160 REU) represents good design rather than just increased Buried Surface Area (BSA), it would be beneficial to see the Binding Energy Density (REU/BSA) reported for the three energy groups.

The authors interpret the correlation between Rosetta binding energy and specific secondary structure patterns (e.g., beta-sheets for strong binding vs. RHH for intermediate energy) as a property of the designs (Line 289). However, because these structures are derived solely from AF2 predictions, they must be treated as computational hypotheses rather than experimental results. Please revise the way this information is being delivered.

Missing evidence of covalent crosslinks being formed in the hydrogel. Please provide mass spec or SDS-page gel results of A-X-A + B-X-B and A-X-A+B-B-B.

Alpha-helix secondary structures have two characteristic "dips" at 222 nm and 208 nm, and a strong positive peak at 192 nm. Please provide the full CD spectra (wavelength 190 nm -260 nm) of the assemblies in Fig. 5f. It’s currently cut off at ~200 nm.

The results in Fig. 5 lack a necessary negative control to decouple the effects of protein entanglement from the effects of high-density covalent crosslinking. At the reported high concentration of 8% wt, the SpyTag/SpyCatcher system can induce gelation in most globular proteins via network percolation, regardless of their internal topology. To prove that the designed 'entangled' domains are the primary drivers of the observed viscoelasticity and stress relaxation, the authors must provide a comparison against a non-entangled control protein of similar molecular weight and hydropathy, processed under identical conditions (8% wt, same crosslinker density). For example, the authors can design a non-entangled analog of their current entangled designs as a negative control.

(Remarks on code availability)
While the provided code and instructions are sufficient to ensure the reproducibility of the computational pipeline, the scientific validity of the 'unconditional design' claim is compromised by potential data bias. The lack of verified sequence diversity in the training dataset, combined with the fact that most of the designed sequences maintain >50% identity to natural homologs, suggests that the model is operating within a narrow, homology-driven subspace. To demonstrate true de novo generative capability, the authors must prove that the model can successfully design entangled topologies that are evolutionarily distant from the training set

Reviewer #4

(Remarks to the Author)
I co-reviewed this manuscript with one of the reviewers who provided the listed reports. This is part of the Nature Communications initiative to facilitate training in peer review and to provide appropriate recognition for Early Career Researchers who co-review manuscripts.

(Remarks on code availability)

Reviewer #5

(Remarks to the Author)
Review of: Deng et al., “Generating Protein Hydrogels with Customizable Stress Relaxation Behavior via Deep Learning-Driven Entanglement Design,”

Summary:

In this manuscript, Deng et al. present an elegant and technically impressive framework for the de novo generation of “entangled” protein dimers, with potential applications for the design of biomedical protein hydrogels. The integration of large-scale computational screening, conditional generative modeling, and experimental validation represents an important methodological advance. Despite the novelty and promise of the approach, the manuscript, in its current form, requires significant clarification, additional benchmarking, and improved interpretation before it can be considered suitable for publication. We hope the authors find the comments below constructive and helpful in strengthening the manuscript.

I co-reviewed this manuscript with one of the reviewers who provided the listed reports. This is part of the Nature Communications initiative to facilitate training in peer review and to provide appropriate recognition for Early Career Researchers who co-review manuscripts.

Major comments:

• The authors mine “entangled” domains from the UniRef sequence database and use these sequences to train a generative machine-learning model, highlighting the success rate of their designs. However, the manuscript does not include a direct comparison between the de novo designed sequences and the natural sequences identified by the authors themselves. In Figure 3, the de novo–generated topologies appear to closely mirror those of natural sequences. The manuscript would be significantly strengthened by experimentally testing representative natural sequences and directly comparing their performance to the generative designs, to assess whether and how the proposed approach outperforms naturally occurring solutions.

• Alongside the previous comment, the authors also perform conditional generation of de novo sequences spanning a wide range of binding energies. It would be highly informative to compare these binding energies to those of the natural sequences mined from UniRef, and to clarify whether the designed sequences enable access to a broader energetic range than is observed in nature.

• The authors rely on the GLN metric as their definition of “entangled” homodimers. The manuscript would be strengthened by showing representative examples spanning the range of GLN values and the corresponding structures (a few such examples appear in the Supplementary Figures). Given that this metric is central to the filtering and selection process, it should be more clearly defined and illustrated in the main text. In addition, it would be helpful if the authors could explain why the search was restricted to C2 symmetries specifically, whether this choice reflects an experimental constraint or a theoretical/design consideration.

Other comments:

Line 18: Please define stress relaxation behavior. This is key to the paper and not defined properly.

Line 22: Please clarify what the properties are in “control properties”

Line 24: Again, please define “entangled proteins”, this is instrumental to the paper, but the definition is not stated in the abstract

Line 28: “in-silico success rate exceeding 70%”, can the authors please comment on whether this is comparable to state of the art or better?

Line 37-39: This statement appears somewhat overreaching. A substantial body of prior work has already established the use of de novo design for engineering protein-based materials. We therefore suggest toning down the claim, particularly with respect to the generality of the proposed framework.

Line 51: Please define “entangled proteins”, this is instrumental to the paper.

Line 53: Please clarify what is p53dim?

Line 58: What are knots and links in protein topologies? This definition is important to to understanding the manuscript.

Line 62-64: The meaning of this sentence is unclear. The authors should clarify what is meant by “varying chain-intertwining across an expansive design space” and how de novo designed entangled domains are being applied in the context of viscoelasticity.

Line 68: Please define “chain intertwining.”

Line 95: Could the authors please clarify what is meant by an “entanglement judge”?

Figure 1: In panel c, could the authors please clarify the origin of the number 12,742,356? Is this value derived from the initial filtering stage? Please also see the earlier major comment regarding the GLN metric—showing the GLN distribution in the main figure would help readers understand the basis for the |GLN| > 0.7 threshold. In addition, in panel g, the term “cellular

organisms" is unclear and should be clarified.

Figure 2: In panel e, the y-axis label is missing.

Line 175: It would be helpful if the authors could place the reported ~70% success rate in the context of existing approaches and clarify whether this performance represents state-of-the-art or better results.

Figure 3: In panel b, could the authors include the number of sequences in each bin of the box plot? For example, how many of the 4,000 designs fall into the pLDDT > 80 bin? In panels d and e, it would be helpful to report the corresponding |GLN| metric for each structural example shown. For the UMAP analysis, please clarify whether it was performed in a one-hot–encoded sequence space or using an LLM-based embedding. Additionally, the UMAP appears to contain fewer than 4,000 points; the authors should comment on this discrepancy. Finally, the last example shown in panel e appears to be a highly "unnatural" dimer—could the authors clarify whether this corresponds to the c0_2898 design

Figure 4: In panel a, what does "Control" refer to? In panel b, could the authors provide the number of sequences in each bin? For consistency, it would be helpful to do the same elsewhere where applicable. In panel e, increasing the visibility of the symmetric unit would improve clarity, as it is currently difficult to distinguish the individual monomers. Additionally, it is interesting that only the smaller (blue) domains lack close sequence identity matches; could the authors comment on this observation and its possible implications?

Line 276-292 and Figure 4: It is well known that Rosetta energy scales with sequence length; therefore, a linear correlation between length and energy units is not particularly informative. A more informative metric would be one that is normalized by length. The authors should consider reporting or switching to a length-normalized energy metric.

Figure 5: In panel a, the main differences between the two modalities used for the different constructs are not clear, particularly for non-expert readers. How are these modalities expected to differ in their biophysical properties, and why is it important to test both? In panel b, it would be helpful to report the molecular weight (MW) of each design. In panel c, the color scheme makes it difficult to identify which profile corresponds to which design; stratifying the designs based on secondary structure could improve clarity. In addition, the interpretation of the different profiles is not immediately obvious, and a schematic illustrating the different modalities would aid understanding. Moreover, the designed domains are sufficiently long to allow quantitative analysis of their secondary-structure content (helix, β-sheet, and coil). Such an analysis would provide critical insight into whether the designs are indeed folding into their intended structures. In panel e, there is no negative control, making it unclear how challenging it is to form hydrogels using the SpyTag/SpyCatcher constructs alone. Including a natural protein as a reference would strengthen the analysis. This comment also applies to Figure 6. Panel d is very nice!

Figure 6: For non-expert readers, these plots are difficult to interpret. The authors should consider including a schematic illustrating what G′ and G″ represent, as well as the meaning of H(τ). In particular, what does the magnitude of the peak in H(τ) signify physically? It appears that most designs exhibit relatively flat H(τ) profiles, and stratification by predicted binding energy does not substantially clarify the observed differences. Are these profiles consistent with the authors' expectations, and if so, how? Binding energy alone seems unlikely to explain the variance in the data; the authors may wish to consider and discuss alternative or additional metrics, such as interface surface area, interface chemistry, or other structural features. Please also see the earlier comment regarding the inclusion of negative controls and natural protein controls.

Line 379: The manuscript refers to the designs as "controllable"; please clarify what parameters are controllable and how this control is achieved.
Methods:

Line 391: What does 50-100 refer to? Do the authors mean amino acids?
Supplementary Information

Line 57: The authors should add a color legend for readability, as the meaning of the yellow, red, blue, and green colors is not defined in the figure or the figure legend.

(Remarks on code availability)

Version 1:

Reviewer comments:

Reviewer #1

(Remarks to the Author)
My comments have been fully addressed in the revised manuscript. I truly believe this paper is a strong contribution to the design of protein based materials with AI. I therefore enthusiastically support its acceptance in its current form.

(Remarks on code availability)

Reviewer #2

(Remarks to the Author)
The authors have made very detailed responses and revisions related to my comments/suggestions, and also to those by other reviewers. They made some additional studies, including more longer simulations, and added more relevant descriptions and discussions, and so on. I think, the manuscript is ok now, and I recommend its publication.

(Remarks on code availability)

Reviewer #3

(Remarks to the Author)
The authors have addressed most of my previous comments and concerns. I have one remaining recommendation regarding terminology on pages 11–12 when discussing the A-X-A + B-X-B and A-X-A + B-B-B systems.

The authors describe A-X-A + B-X-B as "physically crosslinked" and A-X-A + B-B-B as containing "both physical and covalent crosslinks, (page 11, line 356-357)" implying that A-X-A + B-X-B lacks covalent bonds. However, this is incorrect — both networks undergo SpyTag/SpyCatcher ligation, which forms spontaneous and irreversible covalent isopeptide bonds. Both networks therefore contain covalent bonds by definition. The distinction between the two networks is not the presence or absence of covalent bonds, but rather the network topology arising from the functionality of the building blocks: A-X-A + B-X-B has functionality = 2, producing potentially linear covalent chains held together by physical entanglement of the X domains, while A-X-A + B-B-B has functionality = 3, producing a covalently crosslinked branched network with additional physical entanglement contributions. The authors should revise their terminology throughout the page 11-12 to accurately reflect this distinction.

My suggestion on revision “Both A-X-A + B-X-B and A-X-A + B-B-B networks are formed through SpyTag/SpyCatcher covalent isopeptide ligation. However, the A-X-A + B-X-B network has a building block functionality of 2, resulting in linear covalent chains whose gel-like properties arise from physical entanglement of the designed X domains. In contrast, the A-X-A + B-B-B network has a functionality of 3, forming a covalently crosslinked branched network in which both network junction points and physical entanglements contribute to mechanical properties.”

I would recommend acceptance of this manuscript after minor changes of language to improve scientific accuracy.

(Remarks on code availability)
The repository provides detailed and sufficient documentation in the README to navigate the pipeline. The scripts were inspected and do not contain visible syntax errors. Full execution of the pipeline was beyond the scope of this review due to time constraints, but the code structure and documentation appear sufficient for reproducibility.

Reviewer #4

(Remarks to the Author)
I co-reviewed this manuscript with one of the reviewers who provided the listed reports. This is part of the Nature Communications initiative to facilitate training in peer review and to provide appropriate recognition for Early Career Researchers who co-review manuscripts.

(Remarks on code availability)
The authors have addressed all of my concerns thoroughly and thoughtfully. I believe the manuscript is now suitable for publication in its current form. Congratulations to the authors on an excellent and well-executed study.

Reviewer #5

(Remarks to the Author)
The authors have addressed all of my concerns thoroughly and thoughtfully. I believe the manuscript is now suitable for publication in its current form. Congratulations to the authors on an excellent and well-executed study.

(Remarks on code availability)

Open Access This Peer Review File is licensed under a Creative Commons Attribution 4.0 International License, which permits use, sharing, adaptation, distribution and reproduction in any medium or format, as long as you give appropriate credit to the original author(s) and the source, provide a link to the Creative Commons license, and indicate if changes were made.
In cases where reviewers are anonymous, credit should be given to 'Anonymous Referee' and the source.
The images or other third party material in this Peer Review File are included in the article's Creative Commons license, unless indicated otherwise in a credit line to the material. If material is not included in the article's Creative Commons license and your intended use is not permitted by statutory regulation or exceeds the permitted use, you will need to obtain permission directly from the copyright holder.
To view a copy of this license, visit https://creativecommons.org/licenses/by/4.0/

Response Letter

To facilitate reviewing, we have used the following styling approach in our response letter.

Italic text	*Original comments from the reviewers*
Normal text	Direct response to the comments
Blue text	Revised content that corresponds to the change made in the revised manuscript

We have prepared a table of contents to aid navigation.

Table of Contents

Reviewer #1 Comments and Response 2

Reviewer #2 Comments and Response 7

Reviewer #3 Comments and Response 16

Reviewer #4 Comments and Response 25

Reviewer #5 Comments and Response 26

References 47

Reviewer #1 Comments and Response

The authors present an impressive and well-executed study that integrates pretrained protein language models with physically grounded molecular interaction constraints to enable conditional de novo design of entangled protein complexes with programmable binding strengths and topological features. The computational framework is elegant, and the experimental validation—particularly the successful expression, assembly, and rheological characterization of the designed proteins—provides compelling evidence for the effectiveness of the approach.

The work convincingly demonstrates that pretrained protein language models can function as powerful latent encoders for conditional protein generation when combined with physically meaningful constraints, bridging molecular-level design and macroscopic material properties. Overall, this is a high-quality contribution with clear conceptual novelty and strong experimental support.

Reply: We sincerely thank you for your efforts and kind comments.

(1) Figures 1f and 1g primarily provide taxonomic and functional distribution statistics of the curated dataset. While informative, they are not essential for supporting the central methodological and conceptual advances of the manuscript. Consider moving these panels to the Supplementary Information to improve the focus and pacing of the main text.

Reply: Thank you for the valuable suggestion. We have removed these two panels from the main figure to enhance the focus and pacing. The statistics originally presented in these figures have been included in Supplementary Fig. 5a and 5b.

(2) In line 137, the word "nosing" in the phrase "trained to align the denoising and nosing process" should be corrected to "noising."

Reply: Thank you for your careful reading. We have corrected "nosing" to "noising" (page 5, line 152) and have also ensured that all instances of 'noise' in the text are spelled correctly.

(3) In line 159, the capitalization of adaLN-zero is inconsistent with other occurrences in the manuscript (e.g., AdaLN-Zero). Please ensure consistent capitalization and nomenclature throughout the text and figures.

Reply: Thank you for the correction. We have changed all occurrences to 'adaLN-Zero,' in accordance with its first introduction (Peebles, W. et al., arXiv March 2, 2023. https://doi.org/10.48550/arXiv.2212.09748).

(4) In Figure 3a and the associated text, the structure prediction pipeline should be described

more explicitly as involving both ESMFold and AlphaFold2, rather than being presented as image-based results. Clarifying how predictions from the two models are used (e.g., comparison, validation, or filtering) would improve methodological transparency.

Reply: Thank you for your valuable suggestion to improve the methodological transparency of our manuscript. To evaluate the generation performance of TangleDiff, we used two models (ESMFold and AlphaFold2) to predict the structures of generated sequences and computed metrics such as mean pLDDT and entangling percentage based on both predicted structures. This approach helps to confirm that TangleDiff can generate foldable, homodimerically entangled sequences without bias from the structure predictor selected. We have clarified our evaluation pipeline and the motivation of using both ESMFold and AlphaFold2 on page 6, line 185-192, line 198-200, which is also shown as follows:

"To evaluate the unconditional generation performance of TangleDiff, we designed 4,000 sequences with lengths sampled from an empirical distribution and used both ESMFold and AlphaFold2 to predict their homodimeric structures **for comparison**. We assessed the generation performance based on metrics including pLDDT and the percentage of entanglement derived from these predictions. TangleDiff can produce reasonable sequences that fold into reliable entangled homodimeric structures, with an average pLDDT of approximately 80 and an entangling percentage exceeding 80%, as reported by both ESMFold (Figure 3a) and AlphaFold2 (Figure 3b). … The consistency of metrics derived from both ESMFold- and AlphaFold2-predictions confirms TangleDiff's robust generation performance, independent of the structure predictor used."

(5) The Molecular Dynamics section in the Methods requires further clarification and correction. In particular, the reported simulation box length of 1 nm is unrealistically small for a protein homodimer system and would not yield physically meaningful results. The authors should clarify whether this value is a typographical error. If so, the correct box dimensions should be reported. Otherwise, the simulations should be repeated using an appropriately sized solvation box, and the revised setup and results should be described.

Reply: We apologize for this oversight. In our setup, we defined the dimensions of the simulation box to ensure that the protein was placed at least 1 nm away from the box edge instead of using a simulation box length of 1 nm. We have corrected the method description on page 18, line 591-593, which is also shown as follows:

"We defined a cubic simulation box filled with water. The protein was centered in the box, and the dimensions of the box were set to ensure a minimum distance of 1 nm between the protein and the box edge."

(6) The manuscript relies heavily on the absolute Gauss Linking Number (GLN) as the primary metric for identifying and quantifying entanglement. While GLN is well motivated, it would strengthen the work to briefly discuss its robustness with respect to structural uncertainty (e.g.,

prediction noise or thermal fluctuations), or to justify the chosen threshold (|GLN| ≥ 0.7) more explicitly, possibly with references or sensitivity analysis.

Reply: Thanks for the insightful comment. To demonstrate the robustness of GLN values with respect to structural uncertainty, we did a quick molecular simulation of p53dim, a typical entangled homodimer that have been frequently used in previous studies,[1–3] at 300 K and measured both the structural deviations (RMSD) and GLN deviations during the simulation. We put the results in Supplementary Fig. 4, which is also shown below for your reference:

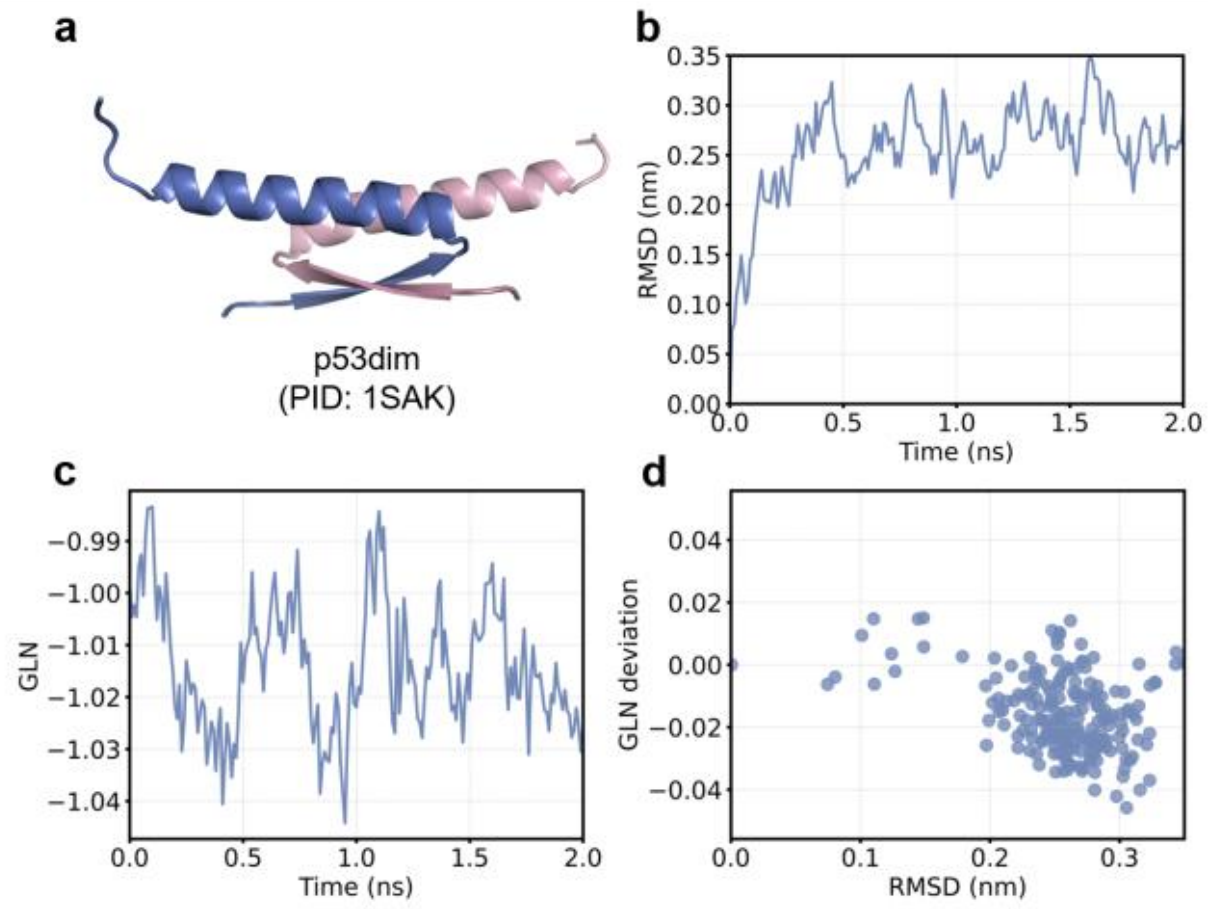

Supplementary Fig. 4. Sensitive analysis of GLN values against structural deviations (e.g. thermal fluctuations). (a) The structure of p53dim, a typical intertwined homodimeric domain used for topological engineering. (b) Root mean square deviation (RMSD) versus time during a 2 ns molecular simulation of p53dim at 300 K. (c) Gauss Linking Number (GLN) versus time during a 2 ns molecular simulation of p53dim at 300 K. (d) Correlation between RMSD and GLN deviation. GLN exhibits little changes during the simulation.

Though p53dim deviated with an RMSD of approximately 0.3 nm from the original, the absolute deviation of GLN remained within 0.05, demonstrating GLN's robustness with respect to thermal fluctuations. Related discussions have been added to the main text on page 3, line 106-109, which is also shown as follows:

"A quick molecular simulation of p53dim,[40] a typical entangled homodimeric domain, demonstrates GLN's insensitivity to thermal fluctuations and small structural deviations (Supplementary Fig. 4), reinforcing its robustness as a detection criterion."

To justify the chosen threshold, we have added a panel in Figure 1 (Figure 1f, also shown below) to show more structure examples that span the range of different |GLN| values, giving a virtual sense of what entanglement extent the 0.7 threshold represents.

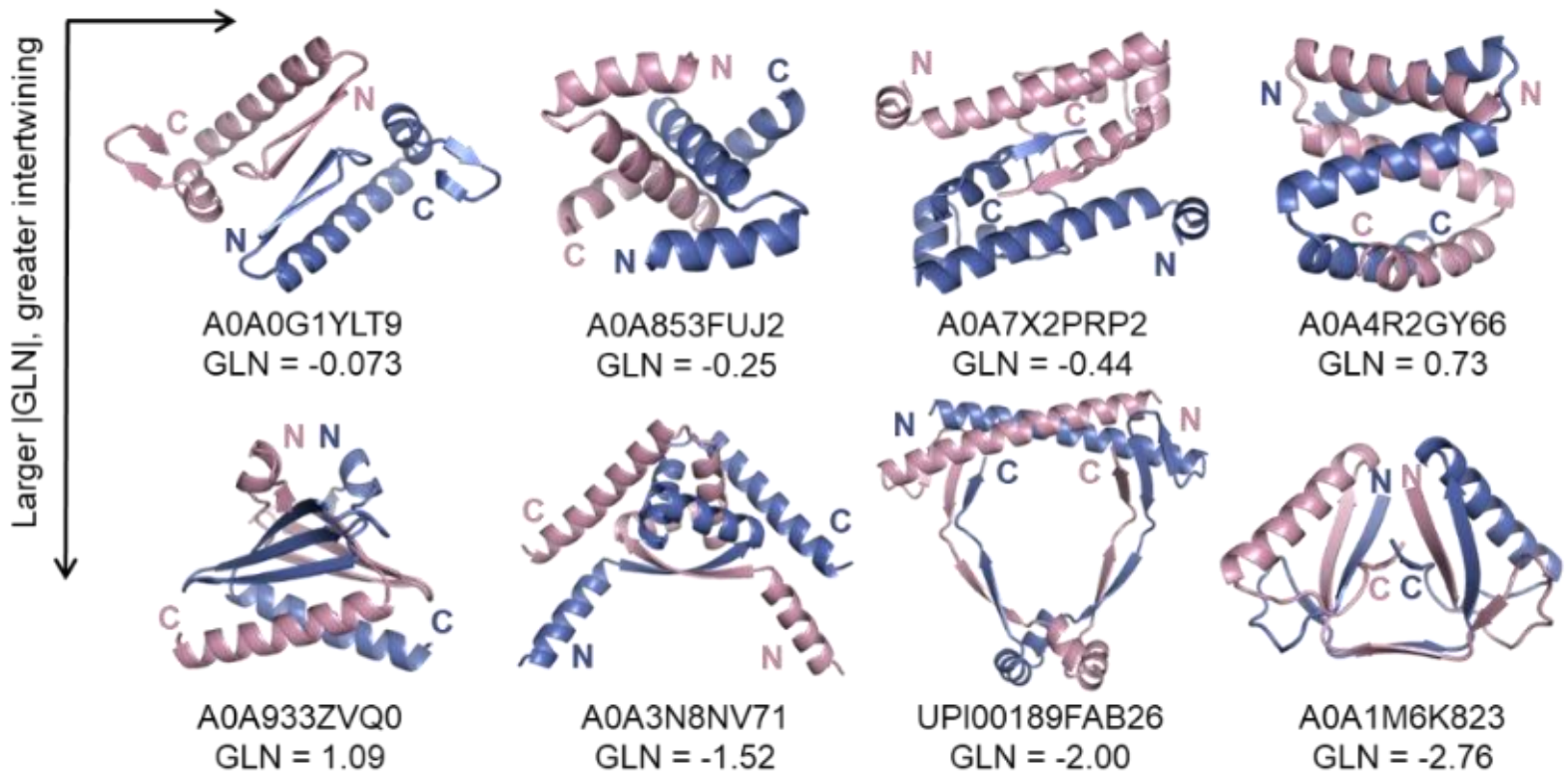

Furthermore, we have added a reference to illustrate that this threshold was selected in accordance with a previous study,[4] which demonstrated that using |GLN| ≥ 0.7 as an entanglement criterion allowed ESMFold to achieve high precision (> 0.8) in identifying entangled structures when tested on two experimentally resolved datasets. We added related discussion and citation in the main text on page 3, line 109-114, which is also shown as follows:

"In this study, we established a |GLN| cutoff of 0.7 to differentiate between entangled and unentangled structures. Although this cutoff may vary depending on different definitions of entanglement or specific requirements for entanglement extent, previous results have shown that using 0.7 as a |GLN| threshold allows ESMFold to effectively identify entangled homodimers with high precision (> 0.8).[41]"

(7) The authors note that the conditioning framework can, in principle, be extended to other molecular-level properties. Including a brief discussion or example (even speculative) of additional conditioning targets—such as entanglement depth, secondary-structure composition, or kinetic stability—would further highlight the generality and future potential of the approach.

Reply: Thank you for your valuable suggestion. We have added a related discussion in the Discussion section on page 15, line 490-494, to highlight the extended application potential of TangleDiff. The addition is as follows:

"It is also important to note that while this study trained TangleDiff with a focus on inter-chain binding energy, the conditioning framework can be extended to other customized molecular-level properties, such as the extent of entanglement, kinetic stability, secondary structure composition—which affects stiffness—and terminal distances, a crucial parameter in topological engineering.[37]"

(8) Given the complexity of the computational pipeline, it would be beneficial to clearly indicate which components (trained models, datasets, scripts) are publicly available and which are not, and to provide guidance on how a reader might reproduce or adapt the workflow for related protein design problems.

Reply: Thank you for the valuable suggestion. We have rewritten the statement in the Data

Availability section and Code Availability section to clarify and detail what is publicly available, as quoted below:

"Data availability

The UniRef50 clusters are available at https://www.uniprot.org/. Training sequences with their calculated features are available at https://github.com/daniel-dpq/TangleDiff.git. The ESMFold-predicted homodimeric structures of training sequences (with their random coil terminals cropped) are available at FigShare (https://doi.org/10.6084/m9.figshare.29604998.v1). Raw data used for all figures/tables are available at https://doi.org/10.6084/m9.figshare.32658021. Raw data of SDS-PAGE are available in the supplementary file.

Code availability

Scripts used for training data screening (structure prediction and metric calculation) are available at https://github.com/daniel-dpq/TangleDiff.git. Codes for model training and inference, and scripts of the evaluation pipeline are also available at https://github.com/daniel-dpq/TangleDiff.git. Scripts for MD simulations and virtual screening of designed sequences prior to experiments are available at https://doi.org/10.6084/m9.figshare.32658021."

To foster the reproducibility of this work, we have additionally included the script that was used for the training data screening pipeline in our GitHub repository (https://github.com/daniel-dpq/TangleDiff.git). We have also provided detailed guidance on how to reproduce our work or customize one's own training by using their own data or specifying their own molecular-level properties.

Reviewer #2 Comments and Response

In this manuscript, the authors made a study on the computational–experimental framework of the design of entangled proteins for application in the stress-relaxing protein hydrogels. The integration of large-scale sequence mining, generative modeling, and materials characterization is fine, and the work is of potential interest to a broad audience. However, some design methodological issues and computational details, and realization processes of the entanglement-crosslinked protein hydrogels are not well described in its current form of the manuscript. The authors should make a major revision on additional analyses, clarification, and discussions as listed in the following.

Reply: Thank you for your insightful comments and for recognizing the potential impact of our study on the design of entangled proteins for stress-relaxing hydrogels. We have meticulously revised our manuscript in accordance with your suggestions and have provided detailed responses to each point below.

1.Only nine designs which were selected after multiple rounds of virtual screening and random sampling (three per binding-energy group) were experimentally tested. The authors did not report overall failure rates or the distribution of discarded candidates, making it difficult to assess representativeness and to exclude success-story bias.

Reply: We appreciate your concern regarding the limited number of designs tested in our study and the potential introduction of bias in the reported experimental success rate. We acknowledge that our capacity to conduct experimental tests was constrained by time and resource limitations. Due to this limitation, the primary goal of the virtual screening and random sampling process was to foster the success of entangled protein design from a limited experimental sample size, demonstrating that TangleDiff is capable of designing experimentally viable entangled proteins for hydrogel synthesis. Accordingly, we designed a virtual screening pipeline followed by a random sampling process. No manual inspection was involved in this process, which minimizes the likelihood of success-story bias. More details of the selection process are described below.

Firstly, to help better illustrate the virtual screening process, we have included Supplementary Fig. 12 (as shown below), which presents the distribution of various metrics utilized during the virtual screening process, clearly illustrating the distribution of both accepted and discarded candidates. This process is to ensure that the structures are reliable, likely to form entanglement, express successfully and be soluble. Additionally, Supplementary Table 1 details the number of remaining candidates after each screening step, highlighting the discarded portion. Related texts have been added on page 10, line 328-331, which is shown as follows.

"The distribution of different metrics employed, and the number of designs retained after each screening step in each group are presented in Supplementary Fig. 12 and Supplementary Table 1, respectively."

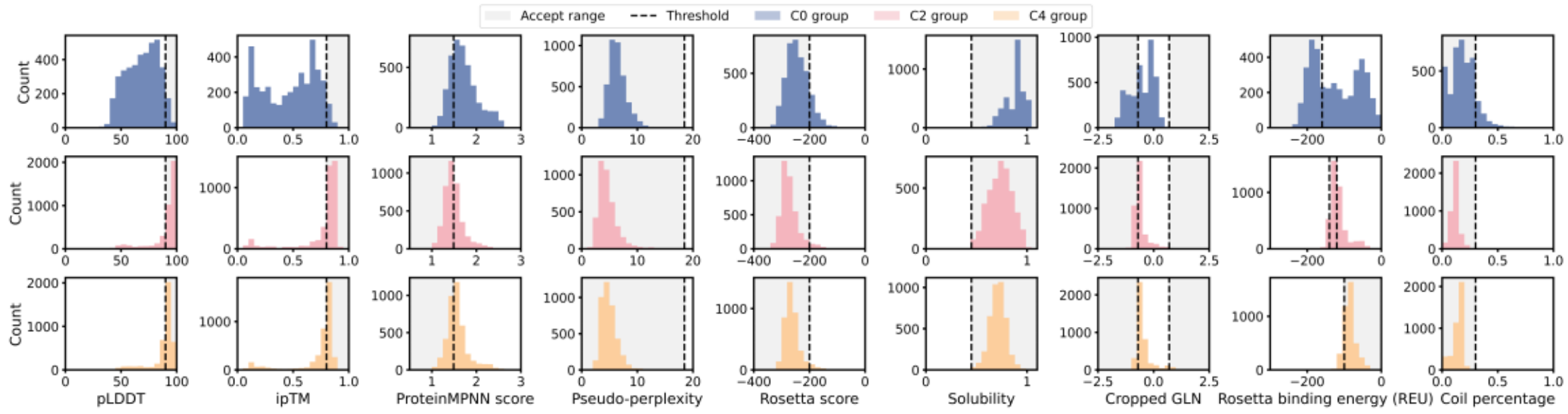

Supplementary Fig. 12. Distribution of various metrics employed during virtual screening for conditionally generated designs. Sequences from different condition groups are color-coded, with shaded areas representing the range of selected designs.

As for random sampling, we have added Supplementary Fig. 13, which displays the distribution of various structural features of the filtered designs. We also illustrate their sequence distribution in Supplementary Fig. 14 by reducing the ESM2 embeddings of these filtered sequences into a two-dimensional space with UMAP. In both figures, we clearly marked the positions of the nine designs selected for experiments. It can be seen that these designs are scattered in the design space and not clustered together.

Supplementary Fig. 13 and Supplementary Fig. 14 are shown below for your reference.

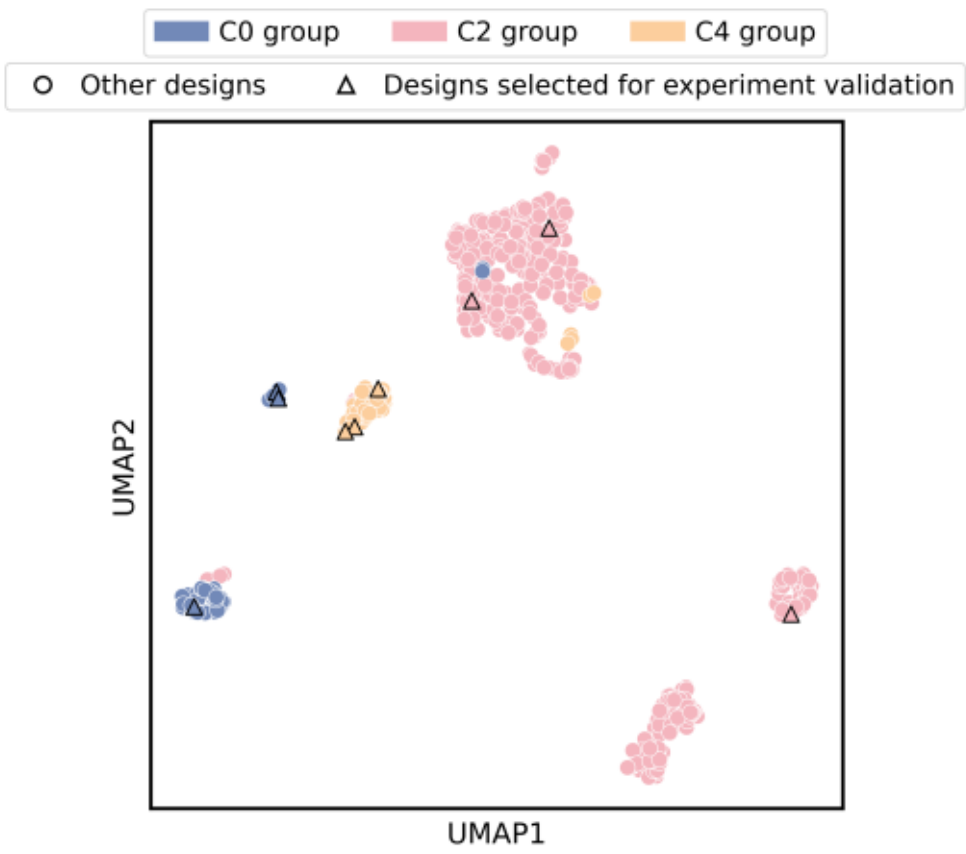

Supplementary Fig. 14. Conditionally generated sequences from distinct binding energy groups are embedded and projected into a two-dimensional space. Each sequence was embedded using ESM2 (650M to create a token-level representation with dimensions of (Length, 1280). This representation was then averaged over the length dimension, yielding a sequence-level representation with dimensions of (1280,). These sequence-level representations were subsequently projected into two dimensions using Uniform Manifold Approximation and Projection (UMAP). Designs selected for experimental validation are marked with triangles.

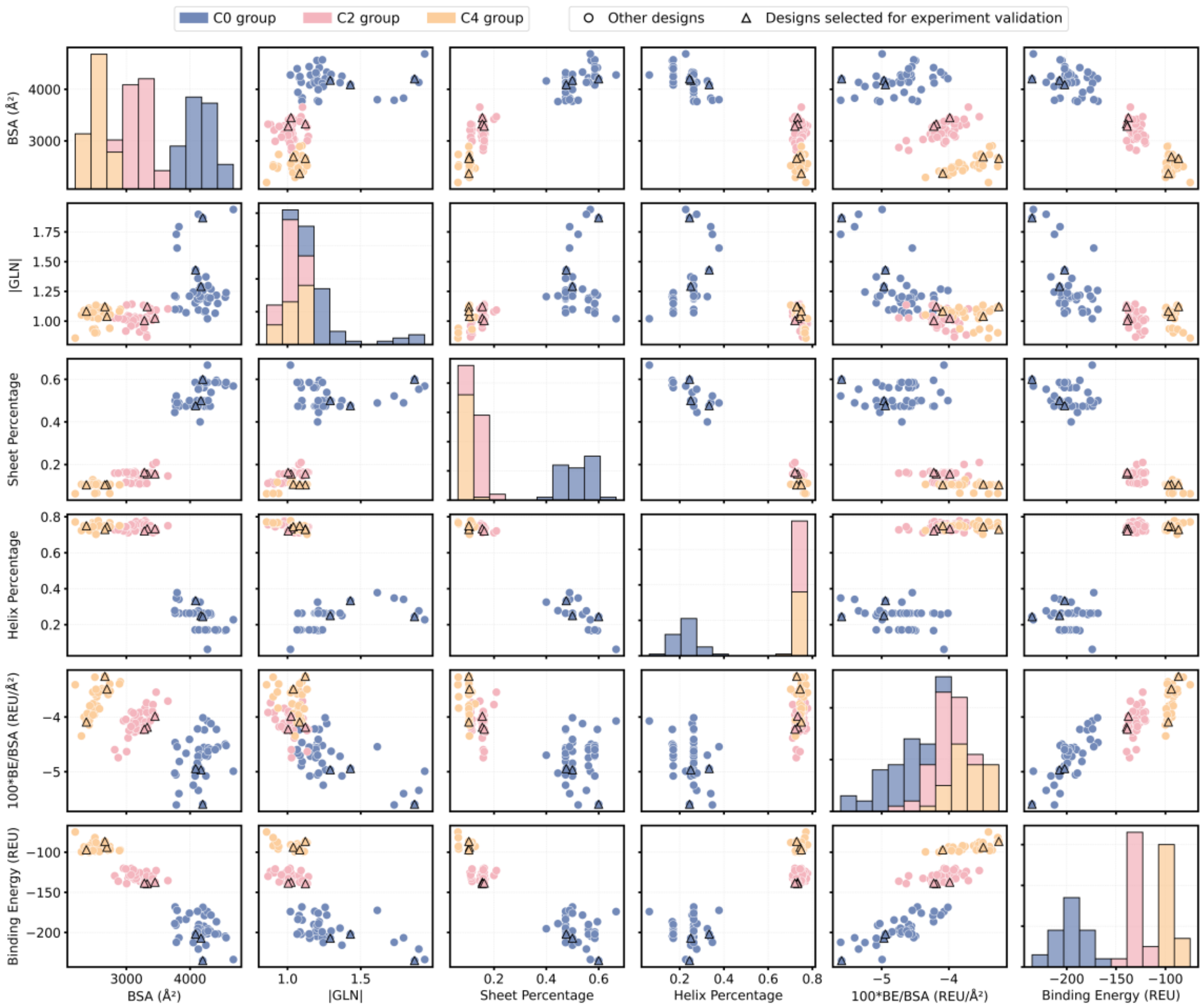

Supplementary Fig. 13. Correlation between various structural features of conditionally generated sequences following filtration through the virtual screening pipeline. The term "100*BE/BSA" denotes 100 times the Rosetta binding energy divided by the buried surface area (BSA). Designs selected for experimental validation are indicated with triangles.

2.The candidate pool sizes differ substantially across groups (~54, 458, and 49). Randomly selecting three designs per group may not adequately capture structural or sequence diversity, and the rationale for this sample size is not justified.

Reply: Thank you for your feedback regarding the candidate pool sizes and selection rationale. We agree that larger sample sizes could capture better structural or sequence diversity and decrease the selection bias. We selected three designs per group to maintain a manageable set for experimental validation while ensuring representation across different binding energy ranges. As mentioned in comment 1, the selected nine designs are not closely clustered across all characteristics, which mitigates concerns regarding success-story bias. Additionally, the success rate of these nine designs was high (7 out of 9 have successfully made into gels), suggesting a very low probability that the overall success rate is low while we happened to select only the successful ones.

To justify the sample size, we have added related discussions in the main text on page 10-11,

line 332-334, which is also shown as follows:

“To ensure a manageable set for experimental validation while maintaining representativeness, three entangled designs from each binding energy range were randomly selected for further experimental validation and protein hydrogel preparation (Figure 4f, g, Supplementary Fig. 1). Clustering analyses demonstrated that these designs are diversely distributed in the design space (Supplementary Figs. 13 and 14)”

3. The MD simulations may be too short to argue the structural stability. For the entangled proteins, the key challenge may be a problem of the kinetic folding accessibility. The current evidences do not clearly distinguish between the structures that are locally stable and those that can realistically fold into the entangled topology.

Reply: Thank you for raising this critical point. We acknowledge the 20-ns simulation in the previous version is too short to present the entanglement stability. In the revised version, we re-implement all MD simulations for 200 ns, which we believe is sufficiently long enough to reveal the structural stability of entangled proteins. The MD result is demonstrated in the Supplementary Fig. 15, which is also shown below:

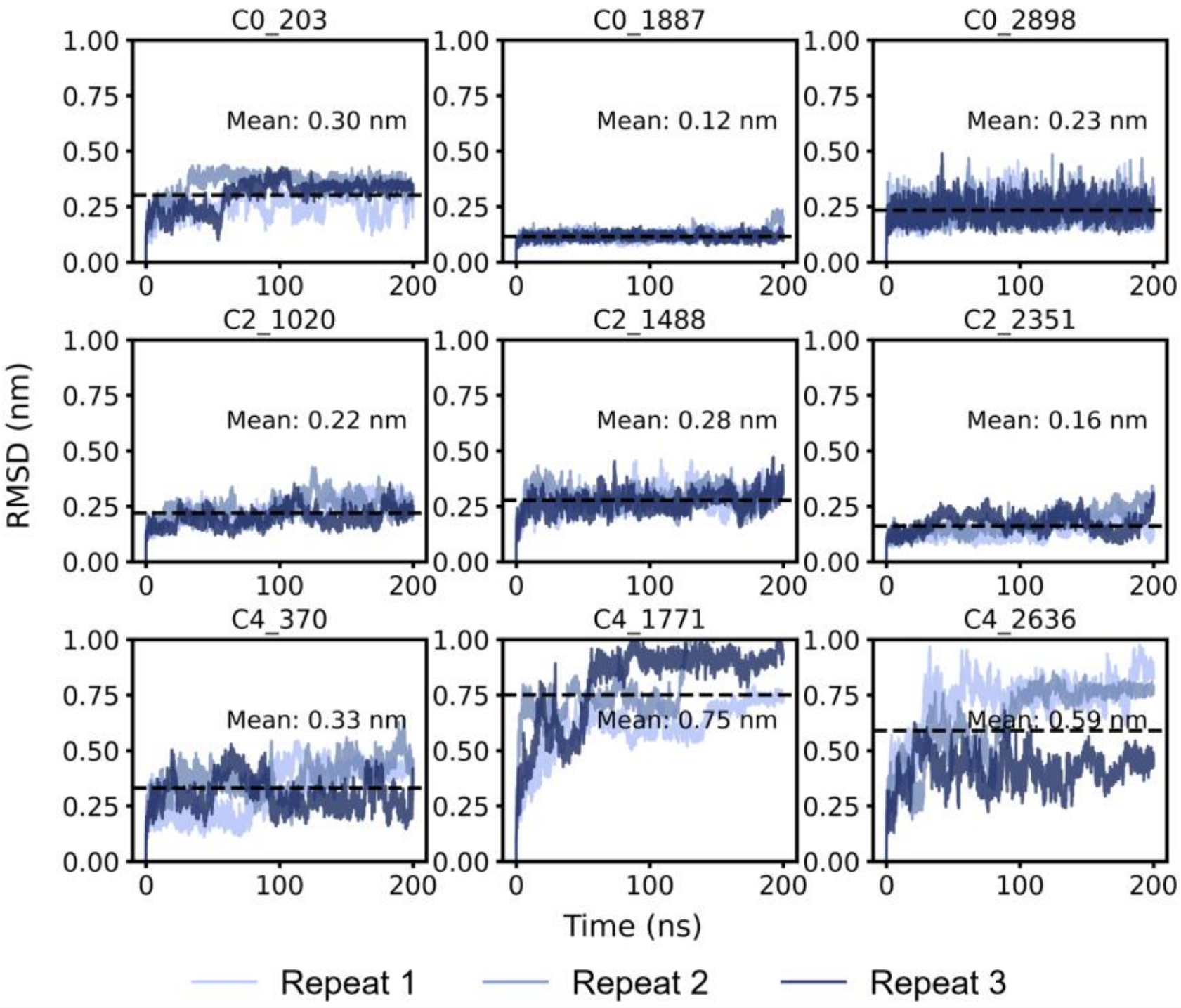

The trends persist in the updated MD results, with designs from C4 group (weakest binding energy) showing the largest structural fluctuations. Based on the new MD results, we recalculated the binding energy with gmx_MMPBSA and updated the values in Figure 4d and Supplementary Table 2. The updated Figure 4d is shown below.

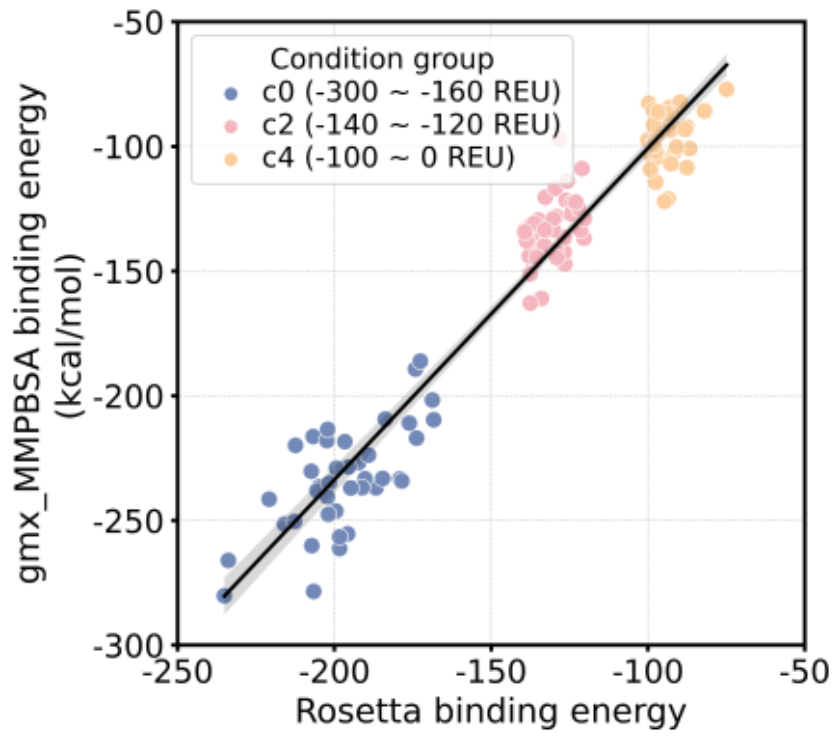

4.The hydrogel networks certainly contain multiple potential relaxation mechanisms (entanglement, SpyTag/SpyCatcher crosslinks, ELP chain dynamics). It remains unclear how non-entanglement-related contributions to the observed relaxation spectra are excluded. This should be discussed well.

Reply: Thank you for this insightful suggestion. To exclude the non-entanglement-related contributions, we have added a negative control group. We prepared A-X-A + B-X-B and A-X-X + B-B-B protein networks using the design C4_1771 under the same conditions. C4_1771 has a similar length to the other designs but has been demonstrated to lack a dimeric form, as confirmed by SEC-MALS results shown in Figure 5d, indicating that it does not form entangled structures. Therefore, the A-C4_1771-A + B-C4_1771-B and A-C4_1771-A + B-B-B networks contain no entanglement crosslinks. The results of these analyses are shown in Supplementary Fig.18, which is also presented below:

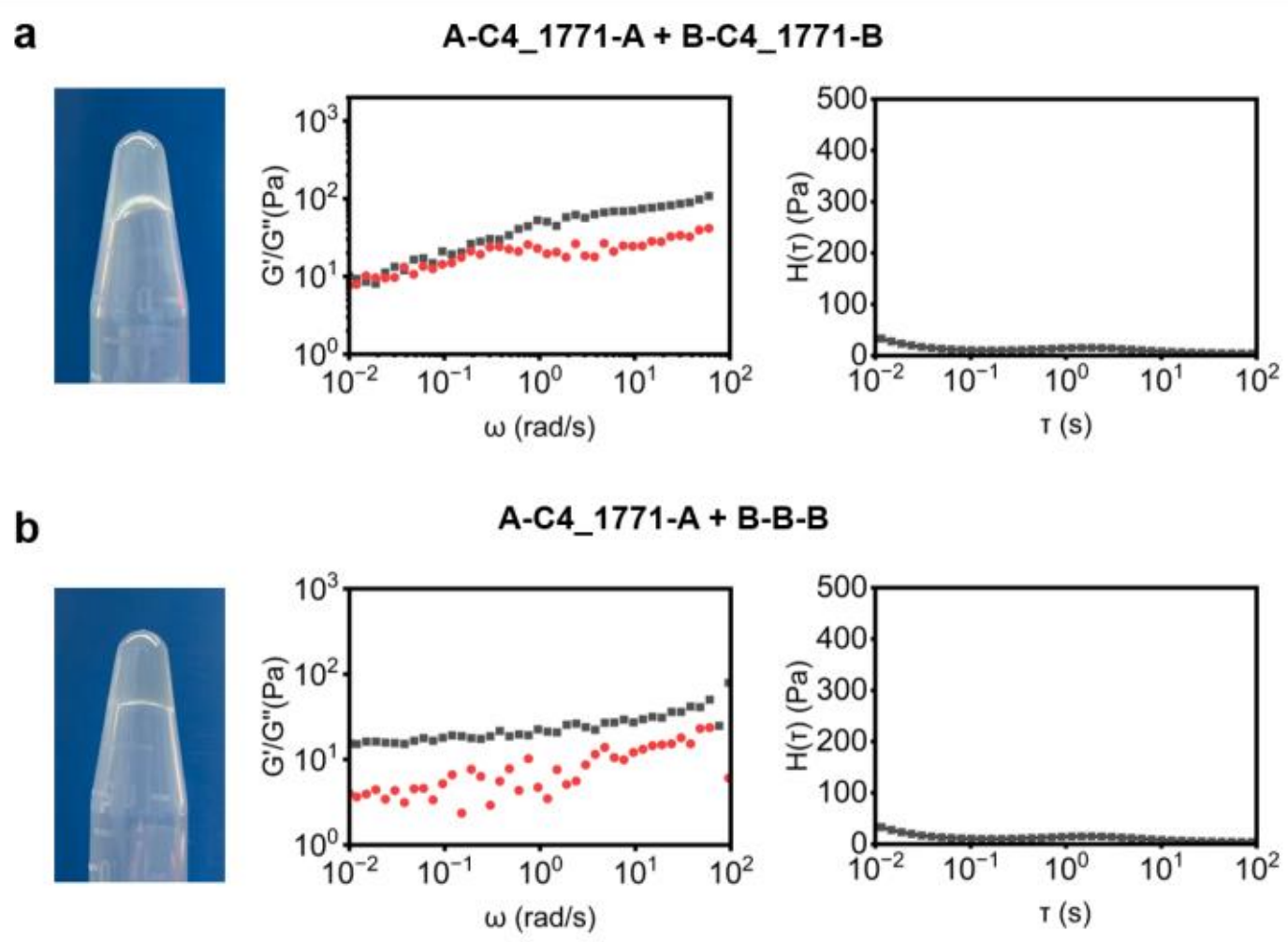

Supplementary Fig. 18. Protein networks with design C4_1771 of the (a) A-X-A + B-X-B type and (b) A-X-A + B-B-B type. Left: a photo of solution mixture. Middle: dynamic mechanical analysis. Right: continuous relaxation spectra.

Unlike the other designs that formed stable gels, both the A-C4_1771-A + B-B-B and A-C4_1771-A + B-C4_1771-B solutions remained as viscous liquids, gradually flowing down the walls of the container. Additionally, these samples did not exhibit continuous modulus curves, even for the A-C4_1771-A + B-B-B network with covalent crosslinks, and their storage

modulus (G’) was significantly lower than that of the other designs. The relaxation spectra H(τ) also showed no evidence of stress relaxation behavior. These results demonstrate that extra physical crosslinks other than covalent crosslinks were necessary for gel formation under our conditions and that the relaxation behavior observed in the hydrogels originated from physical entanglements within the networks, rather than from other compositions such as covalent crosslinks or ELP chain dynamics.

We have added related discussions in the main text on page 14, line 431-440, which is also shown as follows.

“As a negative control, we prepared A-X-A+B-X-B and A-X-A+B-B-B hydrogels from the design C4_1771 that has a similar length to the other designs but does not exhibit dimerization or entanglement (as indicated by the SEC-MALS results). Unlike the other designs that formed stable gels, networks made from C4_1771 of both two types remained viscous liquids that gradually flowed down the walls of the container (Supplementary Fig. 18). Their G’ was significantly lower than that of the other designs and the relaxation spectra also showed no evidence of stress relaxation behavior. This proves that extra physical crosslinks were necessary for the gel formation and that the relaxation behavior observed in the hydrogels originated from physical entanglements within the networks, rather than from other compositions such as covalent crosslinks or ELP chain dynamics.”

5.The authors did not analyze whether inter-chain binding energy is correlated with other structural descriptors (e.g., |GLN|, interface area, secondary structure content). Without such analysis, the independence of conditional control may be not established.

Reply: Thank you for raising this important issue. In response, we have included an analysis of the correlation between inter-chain binding energy and various structural descriptors, including length, buried surface area, |GLN|, sheet percentage, and helix percentage among all training data, which is presented in Supplementary Fig. 10 (shown below for your reference).

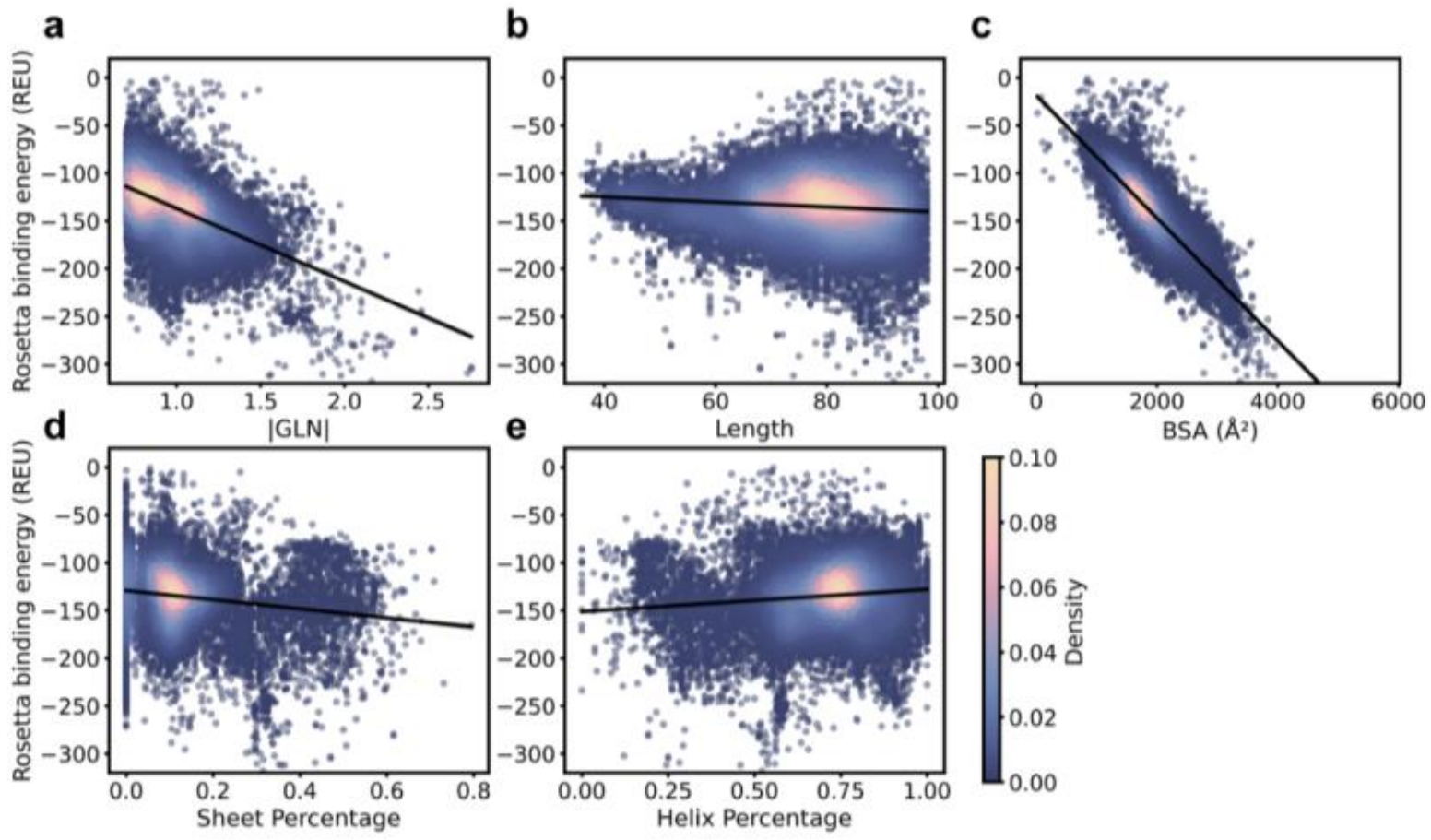

Supplementary Fig. 10. Correlation between Rosetta binding energy and: (a) absolute value of GLN (|GLN|), (b) sequence length, (c) buried surface area (BSA), (d) percentage of beta sheets, and (e) percentage of alpha helices. Binding energies were computed using Rosetta following structure relaxations. Data points represent all training sequences, with a linear fit applied.

Our analysis indicates that the correlation between binding energy and descriptors such as length and secondary structure content is insignificant. However, we found a strong correlation between binding energy and the entangling extent (|GLN|) as well as the interface area. Generally, a larger entangling extent generates greater interface area, leading to higher binding energy. The relaxation behavior of hydrogel networks is closely tied to chain mobility (the ability of chains or chain segments to undergo conformational rearrangements, translation, or reorientation over time). We argue that binding energy is the most direct and dominant factor influencing chain mobility. Additional factors, such as |GLN|, interface area, and binding energy per unit area, play roles in determining the interface binding energy and consequently affect the relaxation behaviors of hydrogels.

While binding energy is a dominant factor, we acknowledge that the other descriptors aforementioned may also influence chain mobility. In our rheological tests, we observed that some hydrogels exhibited relaxation times that did not align with our expectations based solely on their binding energy. We have discussed the possibility that these unexpected findings may arise from other factors in the main text on page 14, line 426-430, which is shown as follows.

"Additionally, relaxation peaks were not observed in hydrogels prepared from designs C4_2636, C2_1488, and C2_2351. We propose that these unexpected findings may be attributed to various factors, such as differing structural features (e.g., buried surface area, secondary structure content, terminal distances) or additional intermolecular interactions (for example, dimeric designs may interact to form higher-order assemblies)."

6.The reported success rate depends on specific threshold choices (say, the pLDDT > 80, and |GLN| ≥ 0.7). Clearly, no sensitivity analysis is provided, and this metric reflects internal pass rates rather than predictive accuracy. Is there a better way to estimate the prediction accuracy?

Reply: Thank you for your feedback. We apologize for the typo in our previous submission; the

reported success rate of over 70% was derived from a pLDDT threshold of > 70, not 80, and we have corrected this in the manuscript. We selected this threshold because predictions above this pLDDT level are generally considered to be ‘confident’.[5,6]

Additionally, we have included a sensitivity analysis in Figure 3c (shown below), which illustrates how the success rates vary with different pLDDT thresholds while keeping the |GLN| threshold constant.

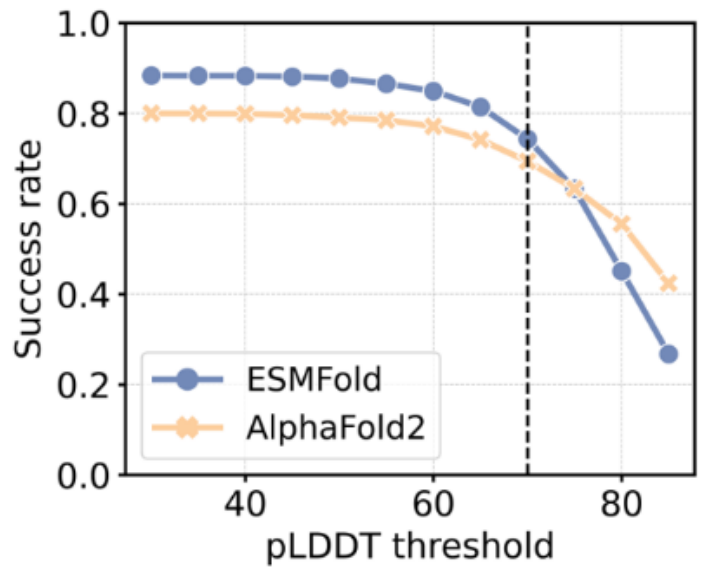

We did not vary the |GLN| threshold (|GLN| ≥ 0.7) in this sensitivity analysis to maintain consistency with the entanglement detection threshold used during the filtering of training data. Specifically, we define |GLN| ≥ 0.7 as indicative of entanglement throughout the manuscript. To justify our chosen threshold, we have added the following discussion in the main text on page 3, line 109-114:

“In this study, we established a |GLN| cutoff of 0.7 to differentiate between entangled and unentangled structures. Although this cutoff may vary depending on different definitions of entanglement or specific requirements for entanglement extent, previous results have shown that using 0.7 as a |GLN| threshold allows ESMFold to effectively identify entangled homodimers with high precision (> 0.8)).[41]”

We have also replaced the original bar plot in Figure 3 with two distribution plots of pLDDT (Figures 3a and 3b, shown below) to provide a more detailed insight into the metrics of the generated sequences.

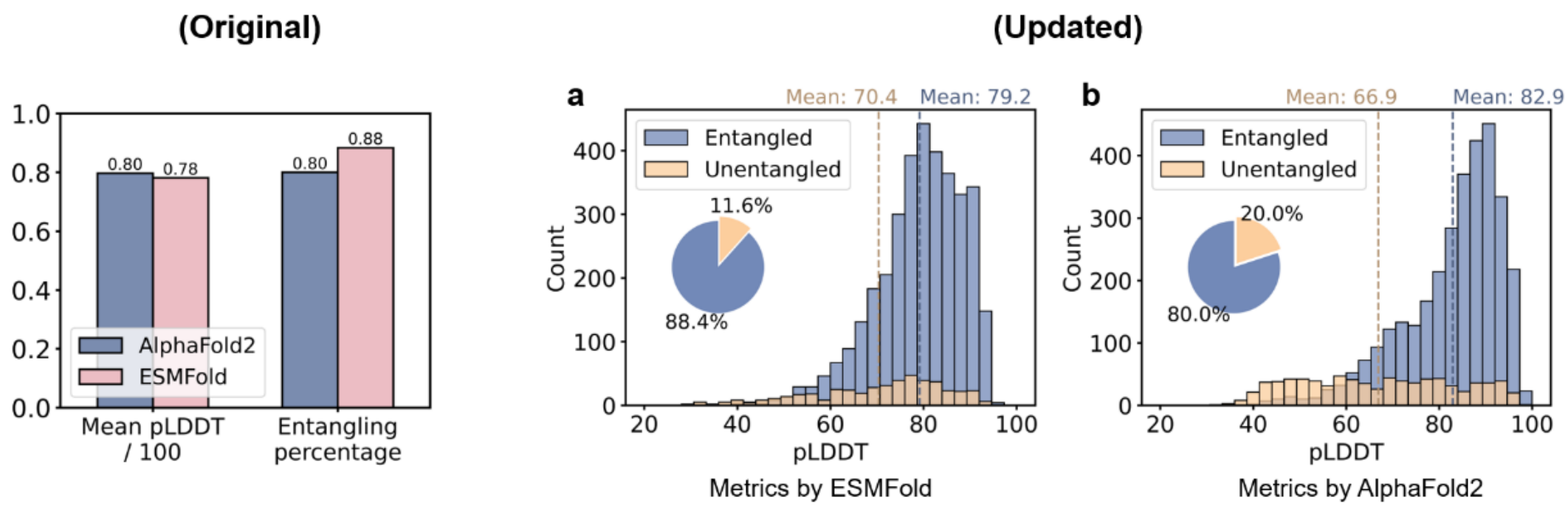

We acknowledge that the reported success rate reflects an internal pass rate. To provide a broader perspective on the performance of TangleDiff, we have compared it to other external sequence generative models by evaluating them using the same metrics, including pLDDT, entangling percentage (|GLN| ≥ 0.7), success rate (pLDDT > 70 and |GLN| ≥ 0.7), and sequence

pseudo-perplexity. These comparisons are presented in Supplementary Fig. 8, as shown below.

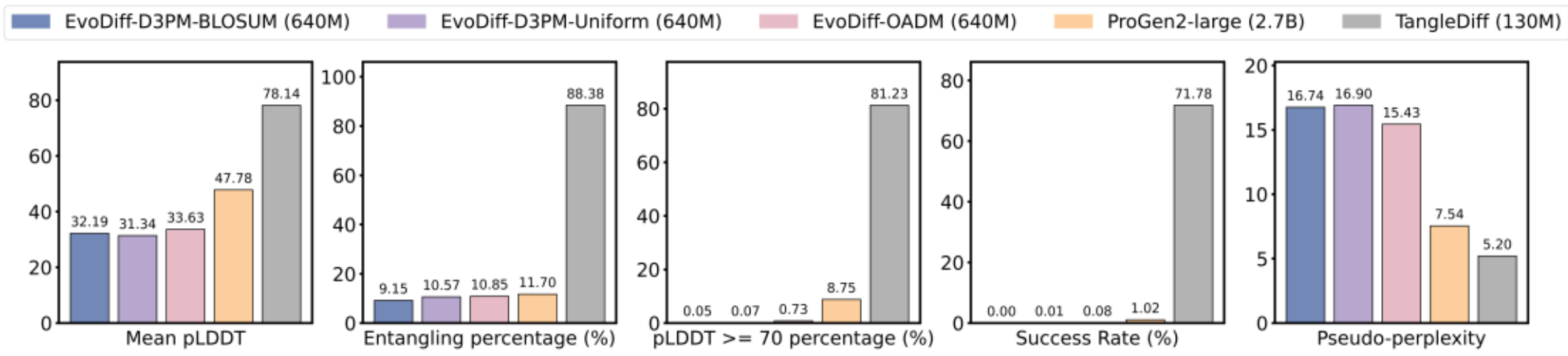

Supplementary Fig. 8. Generation performance of TangleDiff compared to other sequence generation models, including EvoDiff[7] and ProGen2[8]. TangleDiff outperformed the other models in both sequence quality and entangling percentage, achieving a success rate of 71.78% (percentage of designs that are both entangled and have a pLDDT > 70), significantly higher than the next best model, which achieved only 1.02%. All structures were predicted using ESMFold. Pseudo-perplexity is calculated by averaging the prediction probabilities provided by ESM2-650M across all positions in a sequence. This metric estimates how well the masked language model (in this case, ESM2-650M) comprehends the sequence. A lower pseudo-perplexity suggests that the sequence appears more natural and predictable to the model, while a higher pseudo-perplexity indicates that the sequence is unusual or unexpected.

TangleDiff demonstrates an overall *in silico* design success rate of 71.78%, significantly outperforming existing sequence generation models, with the best alternative achieving only 1.02%. We believe these comparisons provide a valuable external perspective on the effectiveness of TangleDiff in relation to our selected metrics. We have also added related discussion in the main text on page 6, line 194-198, which is quoted as follows.

"We consider a design to be successful if it was predicted to be entangled and had a pLDDT greater than 70, a threshold at which the structure prediction is deemed confident.[33,47] Consequently, TangleDiff demonstrates an overall in silico design success rate exceeding 70% (Figure 3c), significantly surpassing that of current sequence generation models, with the best alternative achieving only 1.02% (Supplementary Fig. 8)."

Reviewer #3 Comments and Response

In this work, the authors focus on introducing entanglement into proteins for tuning the mechanical properties. The de novo design of entangled proteins has been achieved with a deep learning-based model of natural sequences with potential entanglement and diffusion-based generative model. This work achieved the design and production of hydrogel macroscopic protein materials via conditional protein design. However, there are limitations of the model building and critical experimental evidence are missing to support several conclusions in this work. I would like the following comments to be addressed.

Reply: Thank you for your thoughtful evaluation of our work. We understand your concerns regarding the limitations in model building and the absence of critical experimental evidence to support certain conclusions in our study. In response to your feedback, we have carefully addressed each of the specific comments you provided, ensuring that we clarify our methodologies and strengthen our experimental foundations. We believe that these revisions will significantly enhance the quality of our work, and we are grateful for your constructive suggestions.

The current approach to entangled homodimer dataset construction faces a fundamental challenge: static folding predictions often deviate from experimental reality, particularly for complex topologies that are kinetically difficult to form. Furthermore, the dataset may be biased toward proteins that resemble existing natural folds, limiting diversity. While the current work will be difficult for this major revision, I have suggestions for future work dataset construction.

The limitation in structural diversity can be mitigated by clustering the UniProt source data at a low identity threshold (e.g., 30%). This ensures the model learns the underlying physics of chain intertwining rather than evolutionary redundancy.

To minimize the gap between computation and lab results, I suggest implementing high-recycling runs in AlphaFold 2 or 3 alongside ESMfold. Using iPAE as a filtering metric—specifically for these oligomeric interfaces—would provide higher confidence in the topological validity of the designs before moving to experimental validation.

That being said, I would like to see a metric added to the SI showing sequence identity distribution of the 79890 sequences that the authors used for this current work.

Reply:

We appreciate your constructive suggestions regarding the future work on dataset construction. We believe that implementing these improvements could significantly enhance the quality of the training data.

In the current work, we have included two panels in Supplementary Fig. 5 (f and g, also shown below) to demonstrate the sequence diversity of our training data. Specifically, Panel f details the number of training clusters relative to the number of training sequences when clustered at various identity thresholds (minimum sequence identity between clusters). Panel g presents the

distribution of pairwise sequence identities among the training sequences. We hope these two panels provide evidence on the diversity of the training sequences used in our study.

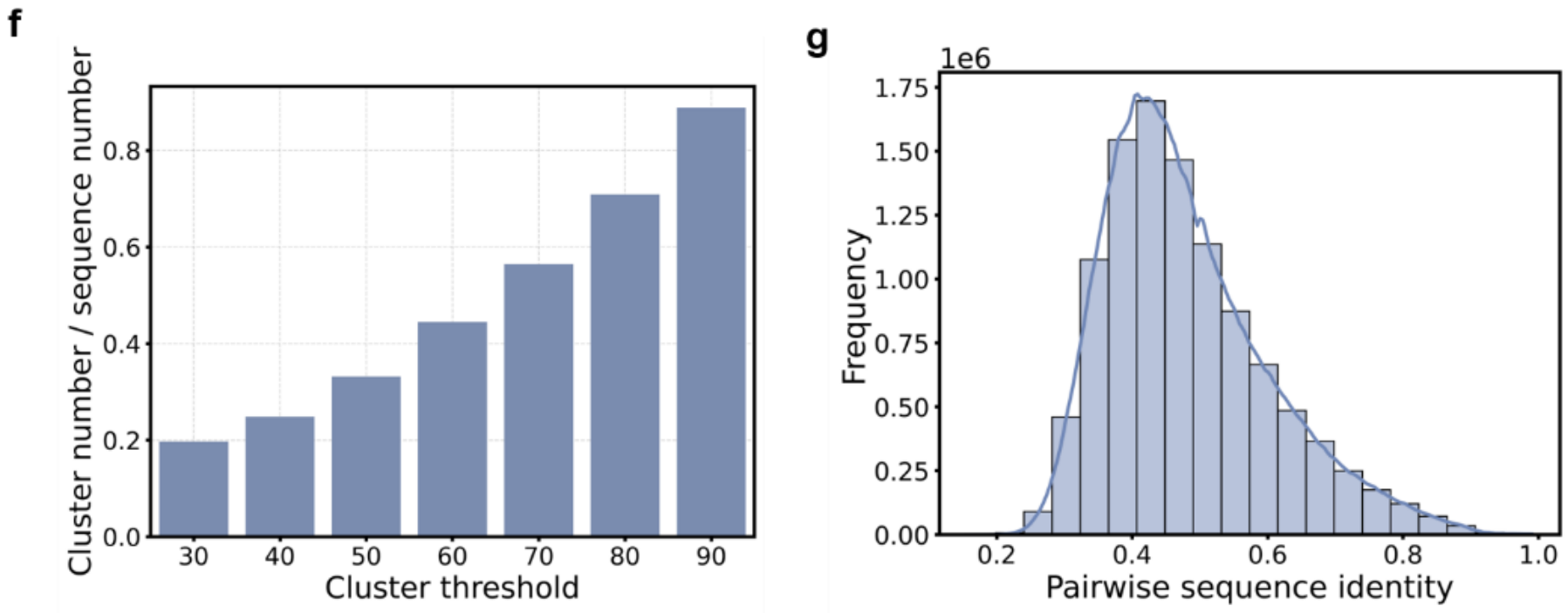

In the Results section of "unconditional design of entangled homodimers" (line 177), the authors claim that the designed sequences show low sequence identity to the training dataset. However, Figure S3b shows a sequence identity distribution centered at ~50% with significant variance (large error bars). In the context of de novo design, 50% identity often represents a level of homology where structural and even functional conservation is expected. Please revise this statement and provide a histogram of the identities rather than just a mean/error bar.

Reply:

Thank you for your valuable comments. In response, we have replaced the boxplots with histograms in Figure 3d (shown below) to provide a more detailed view of the sequence identity distribution.

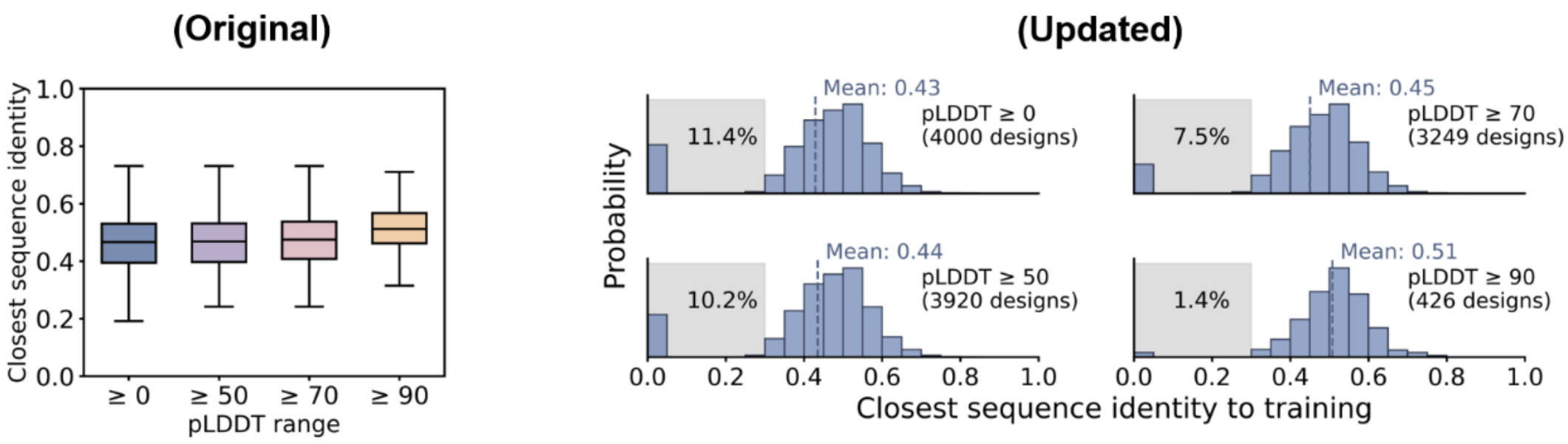

In our revised analysis, we have annotated the percentage of designs with closest training sequence identity lower than 30% (indicated by the shaded region), a threshold below which two sequences are generally considered as evolutionary distant. Specifically, we found that 11.4% of the designs have the closest training sequence identity of less than 30%, and among those with confident pLDDT scores (pLDDT > 70), 7.5% fall below this threshold. This outcome demonstrates TangleDiff's ability to generalize beyond the training homologs.

We revised the original statement in the main text on page 6, line 201-205, which is also quoted as follows:

"Additionally, TangleDiff designs novel entangled sequences. Most of the designed sequences exhibit closest sequence identities ranging from 0.4 to 0.6 compared to the training sequences, even among those with high pLDDT scores (Figure 3d) and across different sequence lengths (Supplementary Fig. 6b). Some designs are evolutionarily distant (closest sequence identity < 30%), highlighting TangleDiff's ability to generalize beyond natural entangled homodimers."

Though some designs have closest sequence identity < 30%, this percentage is relatively low. We believe the suggestions you provide in the first comment would mitigate this issue and we have discussed this potential improvement as a future research direction in the Discussion section on page 15, line 484-489, as quoted below:

"Although TangleDiff generates some novel sequences that are homologously or structurally distant from the training data (with closest sequence identities < 30% or closest TM-scores < 0.5), the proportion of such sequences is limited, which makes TangleDiff more suited for sequence-sensitive property design. This limitation can be likely mitigated by either increasing the diversity of training sequences through a broader-scale search or training model with a lower clustering threshold (e.g., 30%)."

In this work, the authors attempted to design entangled homodimer proteins and several statements in the main text indicate that entangled homodimers form in the designs (eg. line 212 "TangleDiff can design functional entangled homodimers"). However, critical structural evidence shows entangled homodimers. Please try TEM or HRTEM characterization or X-ray crystallography on at least one of the designs. The AF2 predictions in Fig3 can not be used as experimental evidence and SEC only suggests dimer formation but not entanglement or assembly.

Rely: Thanks for your comments. To provide experimental evidence of the structural entanglement in one of the designs, we designed our experiments following the approach that was previously applied to catenane synthesis and entanglement verification (Supplementary Fig. 19) [7,8]. Specifically, we expressed the construct B-C4_370-A. We purified the dimeric products and conducted tobacco etch virus (TEV) protease-mediated cleavage. The purified, partially digested and fully digested products were analyzed using SDS-PAGE. The cyclic monomer band which gets darken when partially digested and disappears when fully digested can be clearly observed. The darkening when partially digested indicates the production of more cyclic monomers during the digestion, which proves the existence of catenanes in the dimeric products, as well as in the structural entanglement in the designs before the covalent linkage was formed.

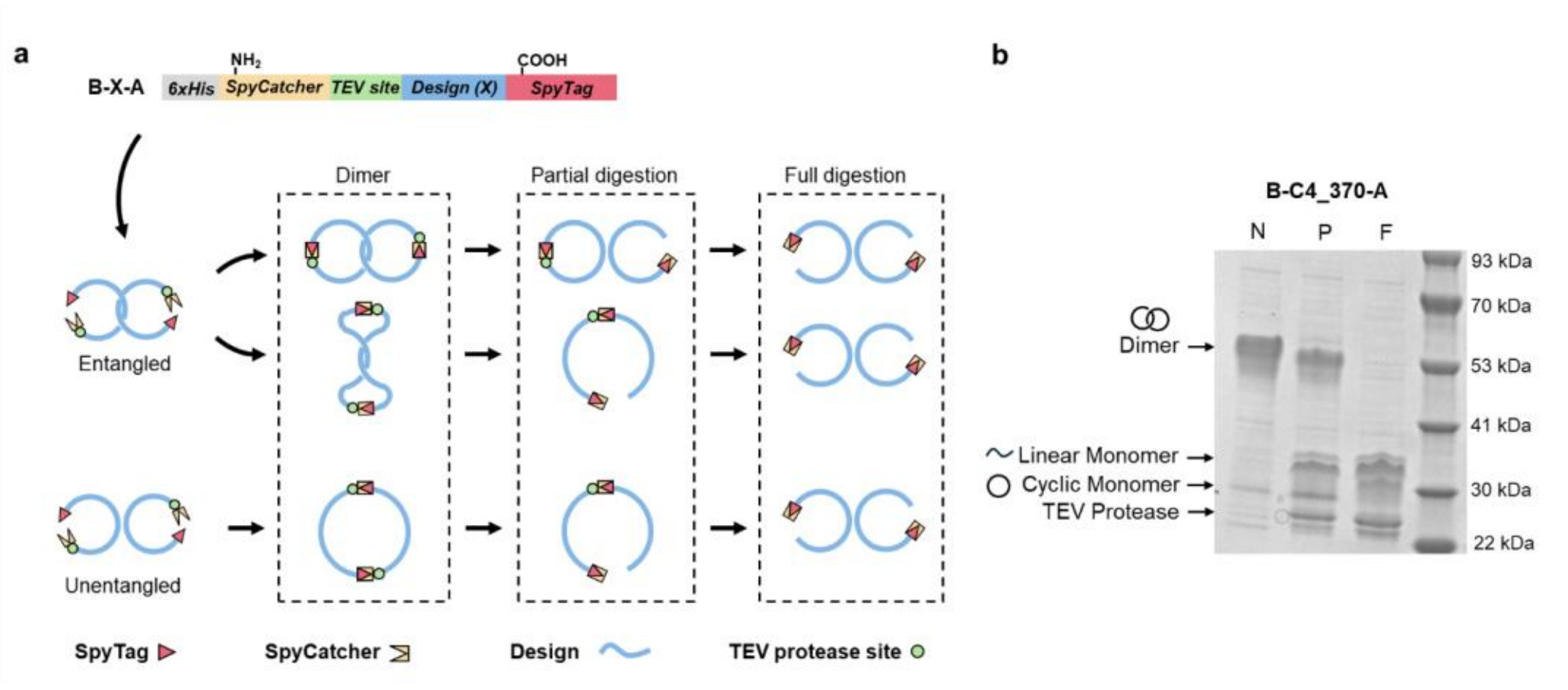

Supplementary Fig. 19. Experimental validation of entangled structures in the designs. (a) Schematic representation of the design of mechanically interlocked proteins as a proof of the entangled structures in designs. We designed the constructs (B-X-A) using the gene cassette previously applied to catenane synthesis.[4,5] We purified the dimeric products and conducted tobacco etch virus (TEV) protease-mediated cleavage. The purified, partially digested and fully digested products were analyzed using SDS-PAGE. The production of cyclic monomers during the digestion process proves the existence of catenanes in the dimeric products, as well as in the structural entanglement in the designs before the covalent linkage was formed. (b) SDS-PAGE analysis of the TEVp digestion products of B-C4_370-A. N = no digestion; P = partial digestions; F = full digestion.

We have added related discussions in the main text on page 14, line 441-448, which is shown as follows.

"To experimentally confirm the structural entanglement, we selected C4_370, which exhibited the most pronounced relaxation behaviors in the prepared hydrogels, as a representative design. We designed the constructs (B-C4_370-A) using the gene cassette previously applied to catenane synthesis.[60,61] Following purification of the dimeric products, we conducted tobacco etch virus (TEV) protease-mediated cleavage (Supplementary Method). The production of cyclic monomers during the digestion process provides evidence for the existence of catenanes in the dimeric products, as well as for the structural entanglement present in the designs prior to the formation of covalent linkages (Supplementary Fig. 19)."

In the results section using Rosetta to calculate the binding energy, line 279, the authors used sequence lengths from 40-50 amino acids. A 40AA protein is kinetically favorable for entanglement folding but such a short sequence usually doesn't have enough surface area to achieve that binding energy score without being "over-packed" or having massive hydrophobic exposure. Since entangled structures naturally bury more surface area than standard dimers, the reported REU values are likely a proxy for the extent of entanglement rather than the specific quality of the interface. To validate that the group (< -160 REU) represents good design rather than just increased Buried Surface Area (BSA), it would be beneficial to see the Binding Energy Density (REU/BSA) reported for the three energy groups.

Reply: Thank you for your valuable comments. In response to your suggestion, we have added a scatter plot in Figure 4 (Figure 4c, shown below) to demonstrate the correlation between

binding energy, binding energy density (100*BE/BSA, BE refers to binding energy) and the binding energy conditions specified during the generation. This plot corresponds to 400 × 5 sequences generated under five different conditions (400 for each).

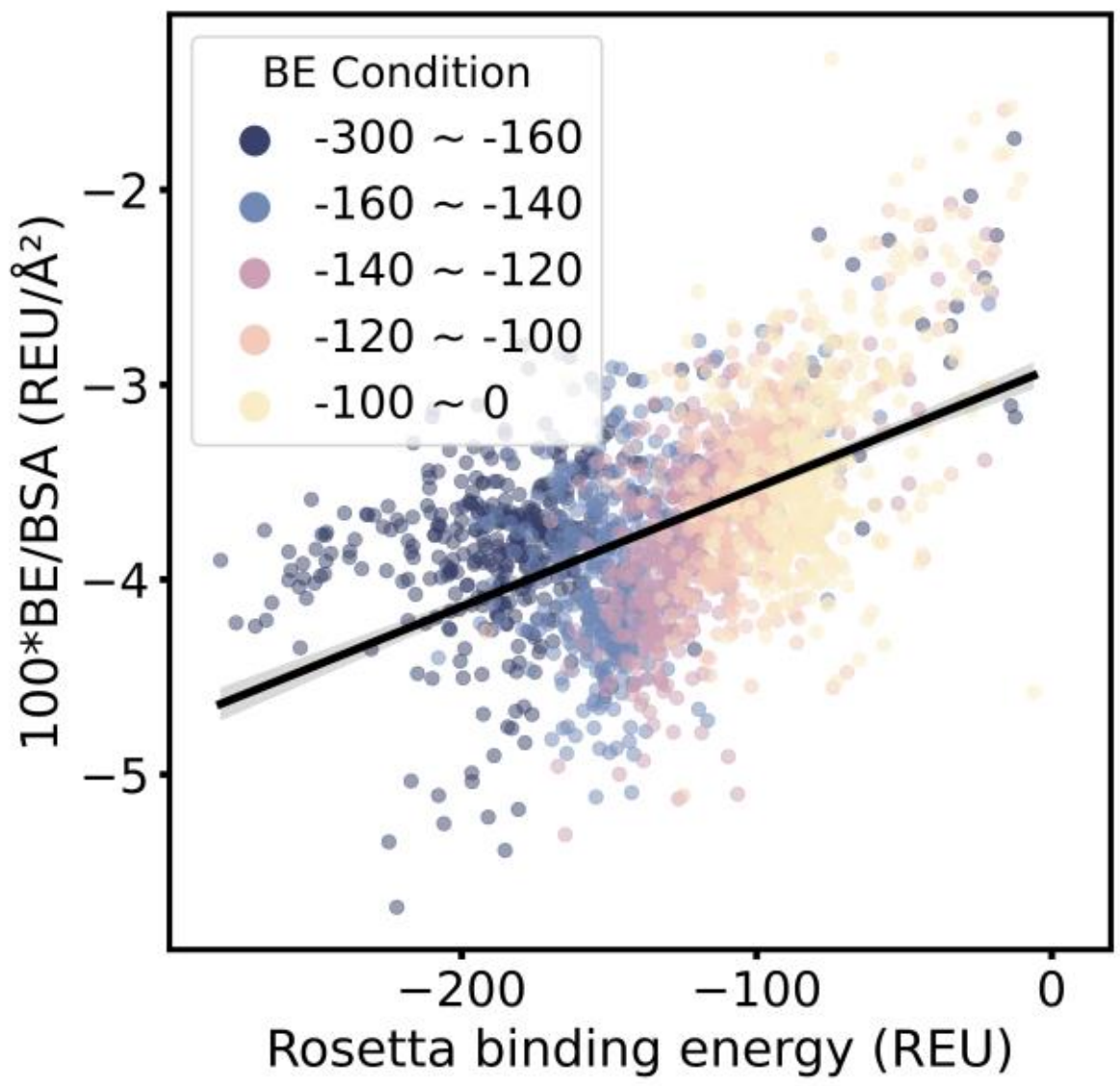

We find that the binding energy density correlates with the binding energy, as well as the binding energy condition. We have added related discussions in the main text on page 10, line 313-314, which is also shown as follows.

"Although not explicitly specified during generation, the binding energy per area of generated designs exhibited a trend consistent with the overall binding energy (Figure 4c)."

For the three groups (C0: < -160 REU, C2: > -100 REU, and C4: -140 ~ -120 REU) we generated for virtual screening and hydrogel preparation, we have also reported the distribution of the binding energy density (100*BE/BSA, REU/Å^2) and its correlation with buried surface area (BSA), GLN, and binding energy in Supplementary Fig. 13, which is presented below.

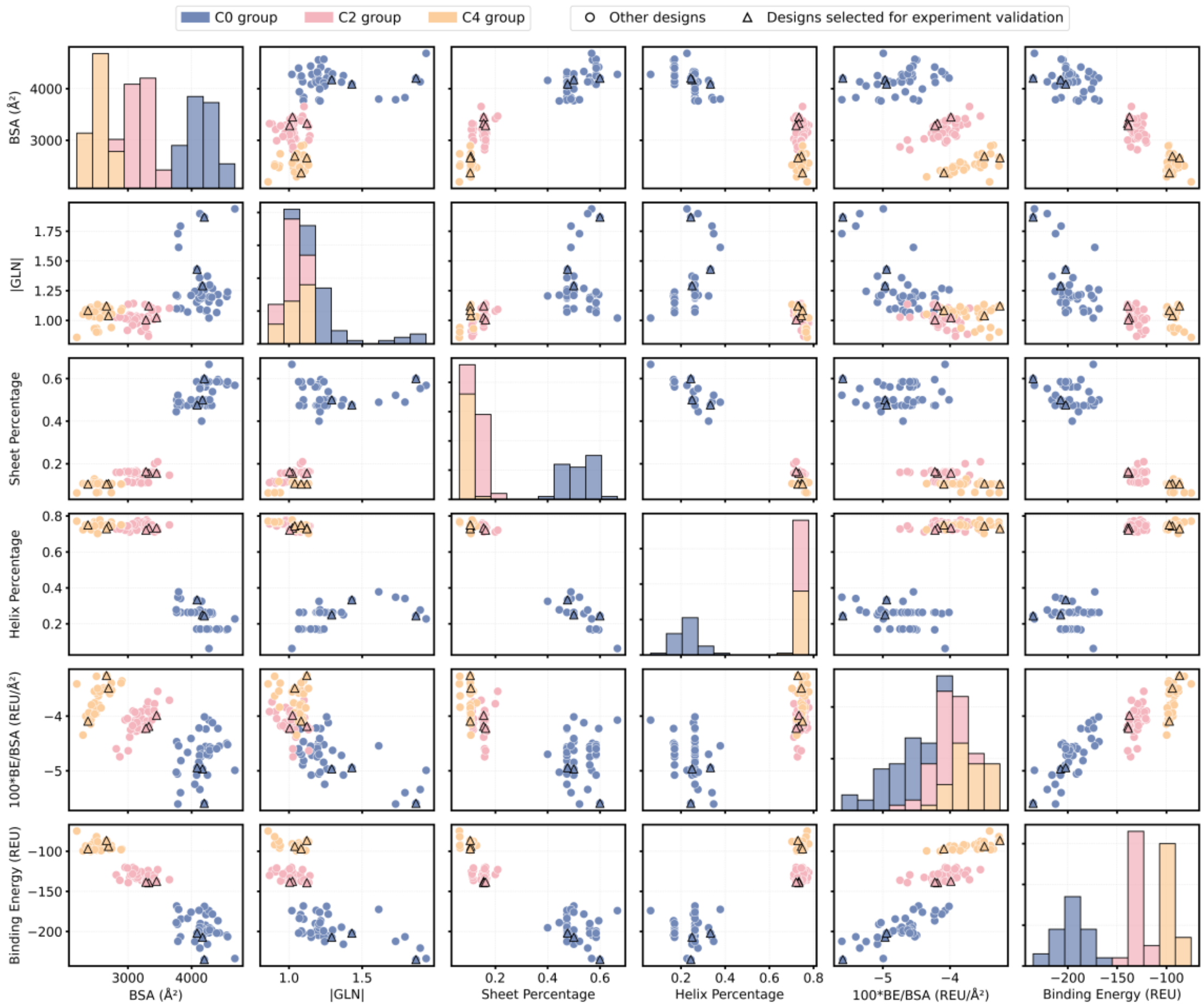

Supplementary Fig. 13. Correlation between various structural features of conditionally generated sequences following filtration through the virtual screening pipeline. The term "100*BE/BSA" denotes 100 times the Rosetta binding energy divided by the buried surface area (BSA). Designs selected for experimental validation are indicated with triangles.

The group (< -160 REU, colored blue) not only demonstrates higher buried surface area and |GLN| values but also reflects stronger binding strength at the unit interface. In this study, we conditioned TangleDiff on different binding energy groups rather than on binding energy per unit area. We suppose that binding energy, an overall reflection of BSA, extent of entanglement, and binding quality, is more closely related to the relaxation behaviors of hydrogel networks.

The authors interpret the correlation between Rosetta binding energy and specific secondary structure patterns (e.g., beta-sheets for strong binding vs. RHH for intermediate energy) as a property of the designs (Line 289). However, because these structures are derived solely from AF2 predictions, they must be treated as computational hypotheses rather than experimental results. Please revise the way this information is being delivered

Reply: Thank you for your careful attention. We have revised the manuscript to clarify that the structures mentioned and demonstrated are predicted by AlphaFold2. This is reflected on page 11, line 335-338, and is quoted as follows:

“Interestingly, designs with distinct binding energies exhibited different entanglement structure patterns in their AlphFold2-predicted structures. In designs with strong binding energies (< -160 REU), entanglements are primarily predicted to be formed with beta sheets.”

We also revised the caption of Figure 4f from “The structures of …” to “The AlphaFold2-predicted structures of …”

Missing evidence of covalent crosslinks being formed in the hydrogel. Please provide mass spec or SDS-page gel results of A-X-A + B-X-B and A-X-A+B-B-B.

Reply: Thank you for your suggestion. While we acknowledge the importance of additional evidence, we believe that the current experimental data sufficiently support the presence of covalent crosslinks in our hydrogel networks.

Specifically, the A-X-A + B-X-B network does not form covalent crosslinks, as each monomer contains only two reactive sites (functionality = 2). In contrast, the formation of covalent crosslinks in the A-X-A + B-B-B network can be inferred by comparing its properties to those of the A-X-A + B-X-B network. The A-X-A + B-X-B demonstrate liquid behavior at low frequency ranges. The A-X-A + B-B-B networks exhibit a higher storage modulus and demonstrate solid behavior even at low frequency ranges.

Alpha-helix secondary structures have two characteristic "dips" at 222 nm and 208 nm, and a strong positive peak at 192 nm. Please provide the full CD spectra (wavelength 190 nm -260 nm) of the assemblies in Fig. 5f. It’s currently cut off at ~200 nm.

Rely: Thank you for your valuable comments. We have conducted a new CD scan and extended the wavelength range to 190 nm – 260 nm, as shown in Figure 5c (shown below). This provides a more complete representation of the CD spectra for the assemblies.

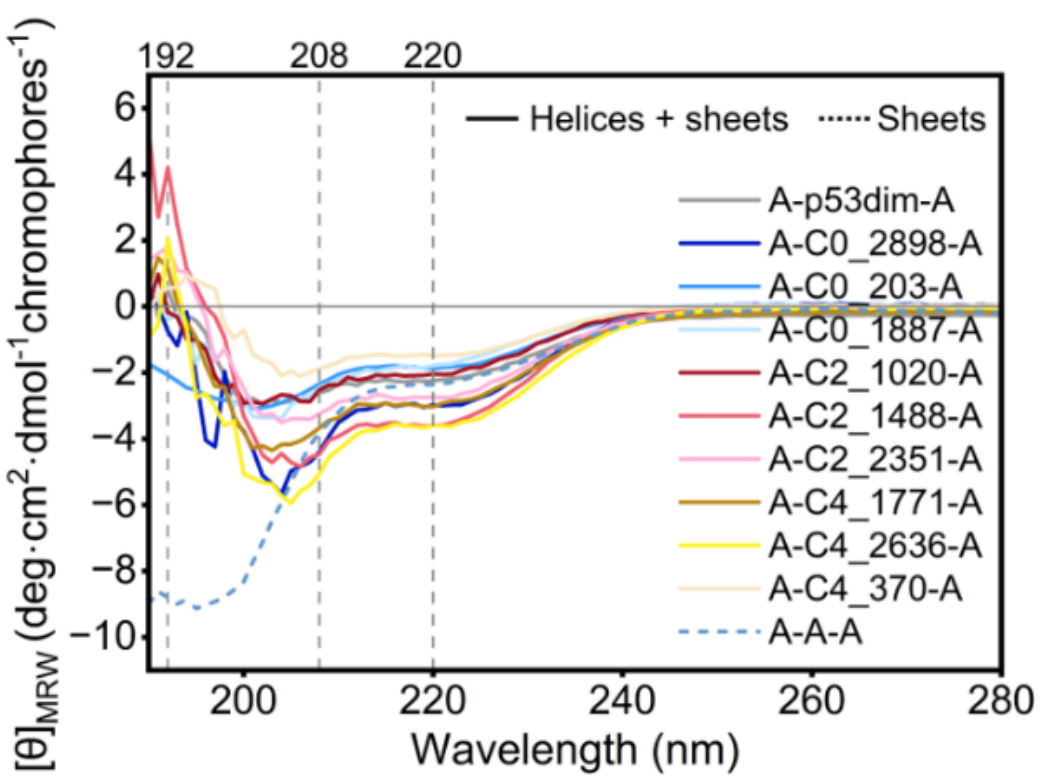

The results in Fig. 5 lack a necessary negative control to decouple the effects of protein entanglement from the effects of high-density covalent crosslinking. At the reported high concentration of 8% wt, the SpyTag/SpyCatcher system can induce gelation in most globular

proteins via network percolation, regardless of their internal topology. To prove that the designed 'entangled' domains are the primary drivers of the observed viscoelasticity and stress relaxation, the authors must provide a comparison against a non-entangled control protein of similar molecular weight and hydropathy, processed under identical conditions (8% wt, same crosslinker density). For example, the authors can design a non-entangled analog of their current entangled designs as a negative control.

Reply:

Thank you for your insightful comments. We have included a negative control in our revised manuscript, consisting of A-X-A + B-X-B and A-X-X + B-B-B protein networks prepared from the design C4_1771 under the same conditions. C4_1771 has a similar length to the other designs but has been demonstrated to have no dimeric form, as confirmed by SEC-MALS results shown in Figure 5d, indicating that it is not entangled. The results are demonstrated in Supplementary Fig. 18, which is also shown as follows.

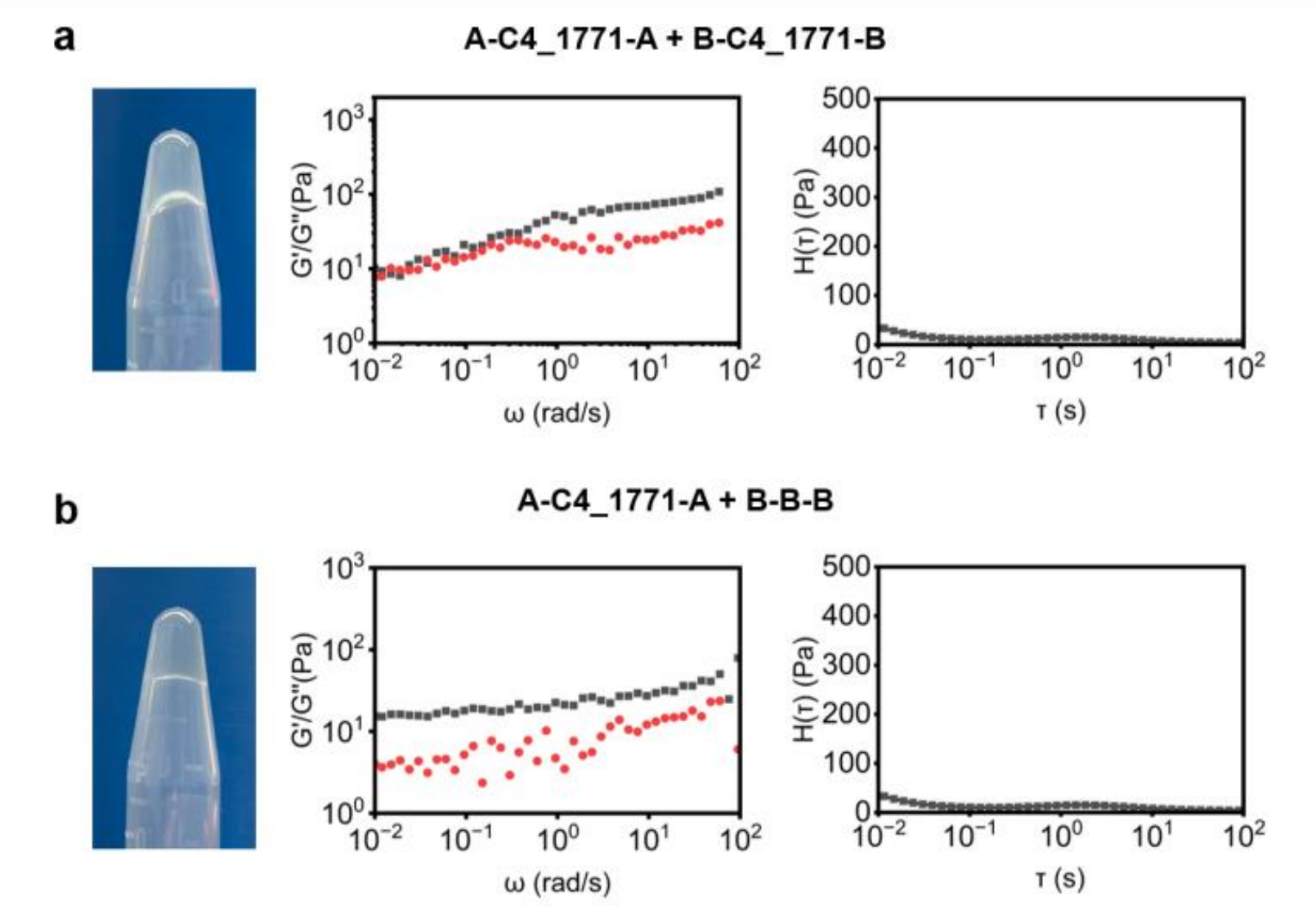

Supplementary Fig. 18. Protein networks with design C4_1771 of the (a) A-X-A + B-X-B type and (b) A-X-A + B-B-B type. Left: a photo of solution mixture. Middle: dynamic mechanical analysis. Right: continuous relaxation spectra.

Unlike the other designs that formed stable gels, both the A-C4_1771-A + B-B-B and A-C4_1771-A + B-C4_1771-B solutions remained as viscous liquids, gradually flowing down the walls of the container. Additionally, these samples did not exhibit continuous modulus curves, even for the A-C4_1771-A + B-B-B network with covalent crosslinks, and their storage modulus (G’) was significantly lower than that of the other designs. The relaxation spectra H(τ) also showed no evidence of stress relaxation behavior. These results prove that extra physical crosslinks other than covalent crosslinks were necessary for gel formation under our conditions and that designed 'entangled' domains are the primary drivers of the observed viscoelasticity and stress relaxation.

We have added related discussions in the main text on page 14, line 431-440, which is also shown as follows.

“As a negative control, we prepared A-X-A+B-X-B and A-X-A+B-B-B hydrogels from the design C4_1771 that has a similar length to the other designs but does not exhibit dimerization or entanglement (as indicated by the SEC-MALS results). Unlike the other designs that formed stable gels, networks made from C4_1771 of both two types remained viscous liquids that gradually flowed down the walls of the container (Supplementary Fig. 18). Their G’ was significantly lower than that of the other designs and the relaxation spectra also showed no evidence of stress relaxation behavior. This proves that extra physical crosslinks were necessary for the gel formation and that the relaxation behavior observed in the hydrogels originated from physical entanglements within the networks, rather than from other compositions such as covalent crosslinks or ELP chain dynamics.”

Reviewer #4 Comments and Response

I co-reviewed this manuscript with one of the reviewers who provided the listed reports. This is part of the Nature Communications initiative to facilitate training in peer review and to provide appropriate recognition for Early Career Researchers who co-review manuscripts.

Reply: We appreciate your effort!

Reviewer #5 Comments and Response

Review of: Deng et al., "Generating Protein Hydrogels with Customizable Stress Relaxation Behavior via Deep Learning-Driven Entanglement Design,"

Summary:

In this manuscript, Deng et al. present an elegant and technically impressive framework for the de novo generation of "entangled" protein dimers, with potential applications for the design of biomedical protein hydrogels. The integration of large-scale computational screening, conditional generative modeling, and experimental validation represents an important methodological advance. Despite the novelty and promise of the approach, the manuscript, in its current form, requires significant clarification, additional benchmarking, and improved interpretation before it can be considered suitable for publication. We hope the authors find the comments below constructive and helpful in strengthening the manuscript.

Reply: We would like to express our sincere gratitude for your positive acknowledgment of the methodological advances presented in our manuscript and for your thoughtful comments. We appreciate the careful and constructive feedback and have made significant revisions in response, which we believe enhance the clarity and interpretation of our work substantially. Below, we provide a detailed point-by-point response to each of your comments.

I co-reviewed this manuscript with one of the reviewers who provided the listed reports. This is part of the Nature Communications initiative to facilitate training in peer review and to provide appropriate recognition for Early Career Researchers who co-review manuscripts.

Major comments:

• The authors mine "entangled" domains from the UniRef sequence database and use these sequences to train a generative machine-learning model, highlighting the success rate of their designs. However, the manuscript does not include a direct comparison between the de novo designed sequences and the natural sequences identified by the authors themselves. In Figure 3, the de novo–generated topologies appear to closely mirror those of natural sequences. The manuscript would be significantly strengthened by experimentally testing representative natural sequences and directly comparing their performance to the generative designs, to assess whether and how the proposed approach outperforms naturally occurring solutions.

Reply:

We appreciate your suggestion regarding the comparison entangled sequences sourced from natural databases.[9] We would like to clarify that our core argument is not that the de novo designed motifs are necessarily better than natural sequences, but that de novo design helps us achieve more flexible specification of the extent of entanglement as well as other properties. The purpose of f and g panels in the original Figure 3 was to demonstrate that the model can design structures that closely mirror natural structures (panel f) or significantly different from known structures (panel g). According to this, we have made the following clarifications and

revisions.

First, we have conducted additional computational analyses between the de novo designed sequences and natural sequences to highlight the advantage of doing de novo design compared to mining natural sequences:

1) The de novo design approach generates novel sequences that extend beyond natural sequence space.

We have replaced the original boxplot in Figure 3 with specific sequence identity distribution data to provide a detailed depiction of sequence novelty.

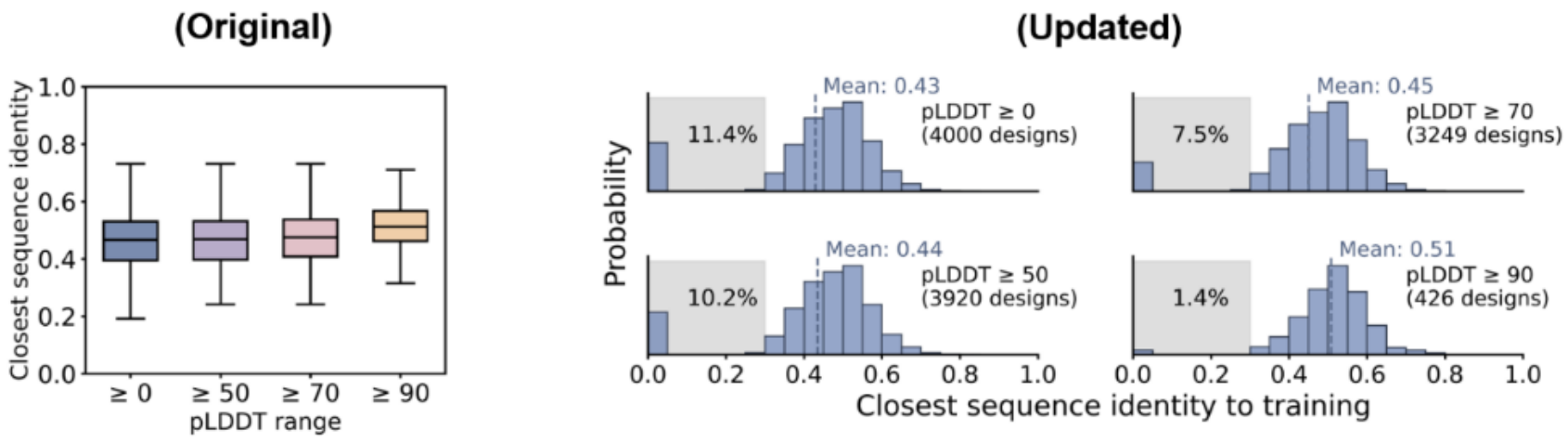

TangleDiff designs entangled sequences that exhibit an average sequence identity of 0.43 compared to training/natural sequences. 11.4% of the designs have the closest training sequence identity of less than 30%, and among those with confident pLDDT scores (pLDDT > 70), 7.5% fall below this threshold.

To assess the structural similarity between our designs and training sequences, we utilized Foldseek to report the TM-scores for the closest structures found in the training dataset. The results are presented in Supplementary Fig. 7 (shown below).

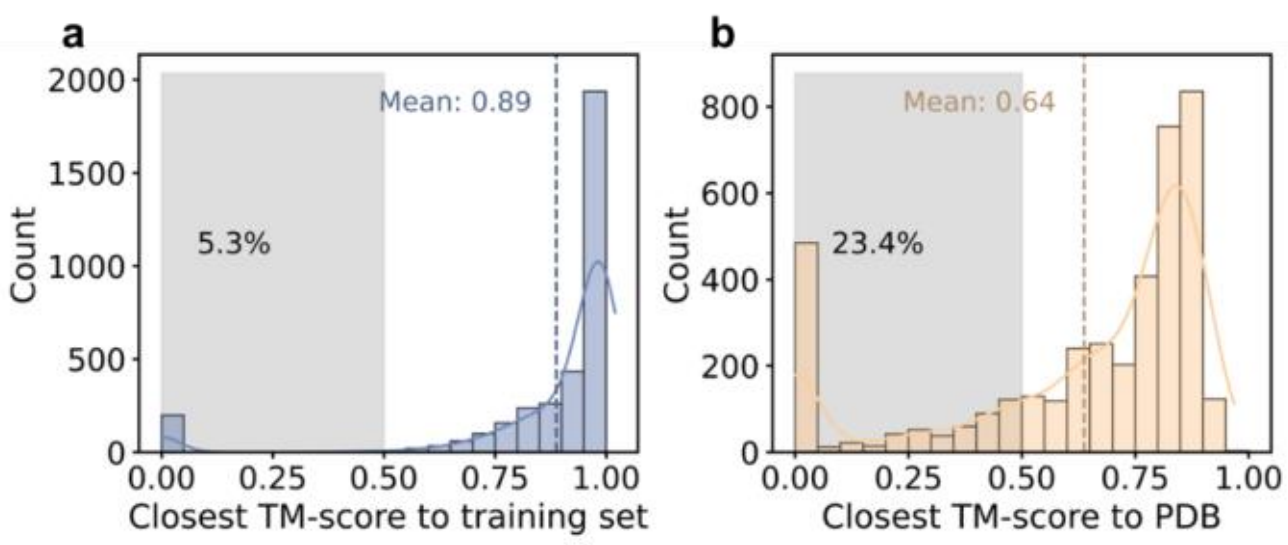

Supplementary Fig. 7. Structural distance of 4,000 unconditionally sampled designs to the (a) training structures and (b) PDB database. The closest TM-score was reported by Foldseek.[7]

5.3% of designs have closest TM-scores < 0.5 compared to the training set. While sequence novelty is evident, structural novelty is more constrained, suggesting that TangleDiff is more suited for sequence-sensitive property designs, such as binding energy, as discussed in this work. We have discussed this limitation and propose potential solutions in the Discussion section on page 15, line 484-489, which is quoted as follows:

"Although TangleDiff generates some novel sequences that are homologously or structurally distant from the training data (with closest sequence identities < 30% or closest TM-scores < 0.5), the proportion of such sequences is limited, which makes TangleDiff more suited for

sequence-sensitive property design. This limitation can be likely mitigated by either increasing the diversity of training sequences through a broader-scale search or training model with a lower clustering threshold (e.g., 30%)."

We have also compared the binding energy distribution of natural sequences with that of the generated designs. Our findings indicate that the designed sequences extend beyond natural boundaries, featuring binding energies not observed in nature (Supplementary Fig. 11; further details can be found in the next comment).

2) The de novo design approach enables the specification of desired conditions or features during generation, such as the inter-chain binding energies demonstrated in this work.

We have demonstrated that both the binding energy and binding energy density can be effectively guided during the generation (Figure 4bc, shown below).

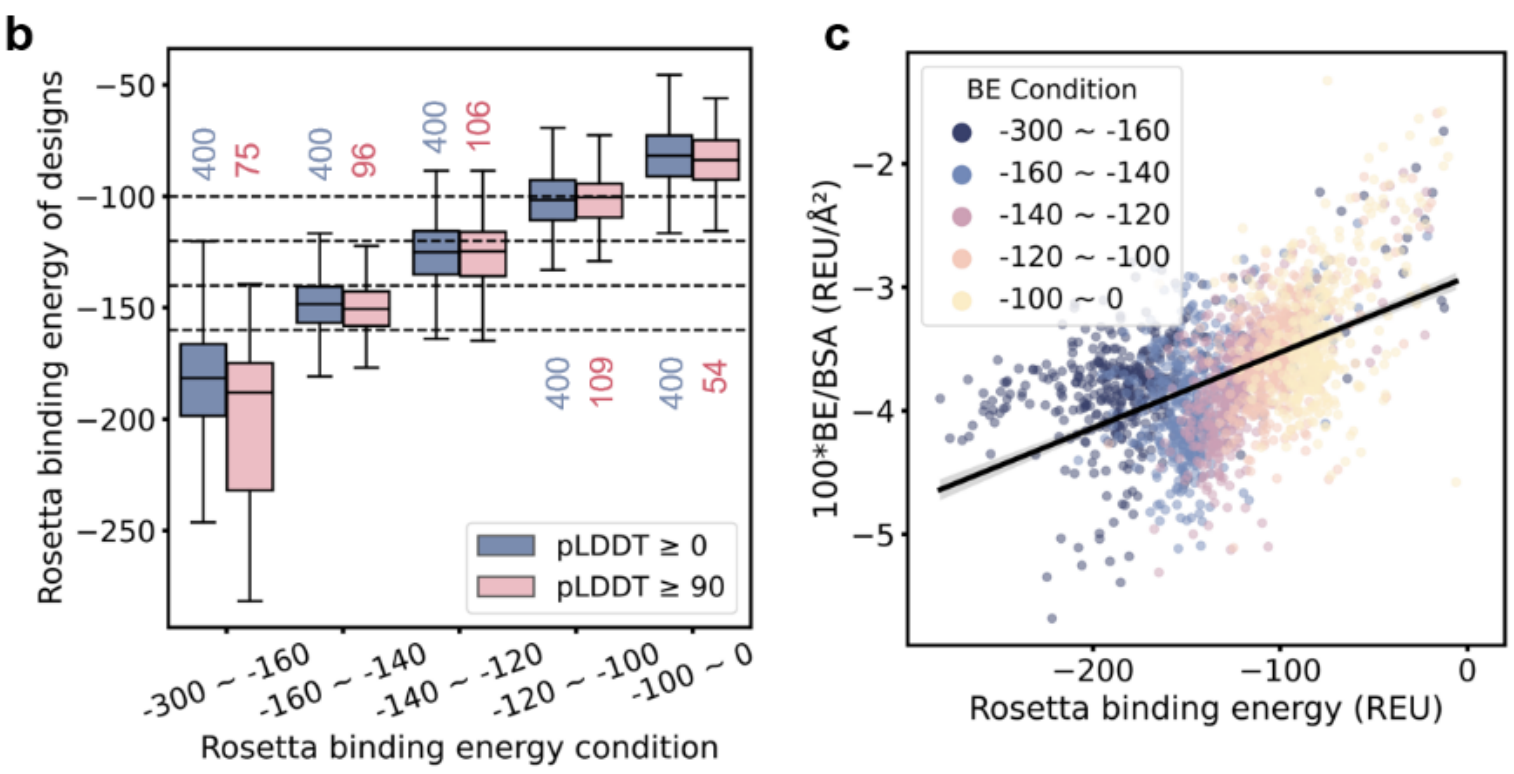

We have also discussed other possible features or conditions that can be explored in the future in the Discussion section on page 15, line 490-496, which is also shown as follows.

"It is also important to note that while this study trained TangleDiff with a focus on inter-chain binding energy, the conditioning framework can be extended to other customized molecular-level properties, such as the extent of entanglement, kinetic stability, secondary structure composition—which affects stiffness—and terminal distances, a crucial parameter in topological engineering.[37] The diffusion framework of TangleDiff also enables seamless integration of a property discriminator, facilitating customized conditional generation based on various criteria without the need for additional training.[63]"

We have discussed these two advantages mentioned above in the Discussion section on page 14, line 458-460, which is also shown as follows.

"In contrast to entangled protein mining from natural sources,[41,62] our de novo design approach generates novel protein sequences and structures that may not exist in nature while enabling tailored designs that meet specific criteria."

• *Alongside the previous comment, the authors also perform conditional generation of de novo sequences spanning a wide range of binding energies. It would be highly informative to*

compare these binding energies to those of the natural sequences mined from UniRef, and to clarify whether the designed sequences enable access to a broader energetic range than is observed in nature.

Reply: Thank you for your valuable suggestions. We have compared the binding energies of the generated sequences with those of the natural sequences mined from UniRef. The results can be found in Supplementary Fig. 11, which is also displayed below.

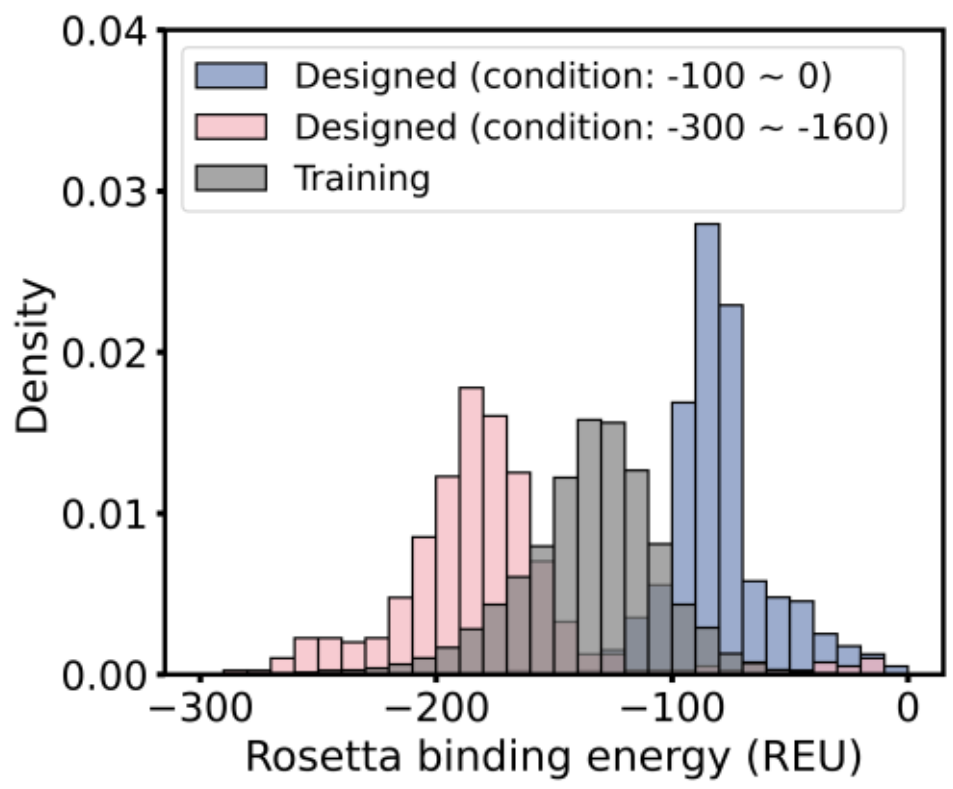

Supplementary Fig. 11. Binding energy distribution of 400 designed sequences generated with condition -100 to 0 REU, 400 designed sequences generated with condition -300 to -160 REU, and all training sequences.

Our findings indicate that the designed sequences cover a broader energetic range than what is observed in nature, further demonstrating the generalization capabilities of TangleDiff. We have included related discussions on page 10, line 315-317, and the relevant passage is quoted as follows:

"Furthermore, the binding energy range of these designs can even exceed those of the natural sequences mined from UniRef (Supplementarty Fig. 11), demonstrating TangleDiff's strong generalizability beyond nature."

• *The authors rely on the GLN metric as their definition of "entangled" homodimers. The manuscript would be strengthened by showing representative examples spanning the range of GLN values and the corresponding structures (a few such examples appear in the Supplementary Figures). Given that this metric is central to the filtering and selection process, it should be more clearly defined and illustrated in the main text. In addition, it would be helpful if the authors could explain why the search was restricted to C2 symmetries specifically, whether this choice reflects an experimental constraint or a theoretical/design consideration.*

Reply: Thank you for valuable suggestions. We have extended the discussion and illustration of GLN metric in both main text and figures. We added eight example structures in Figure 1f (shown below) with different |GLN| ranging from 0 to 2.8, giving a direct virtual sense of how |GLN| correlates with the entanglement extent.

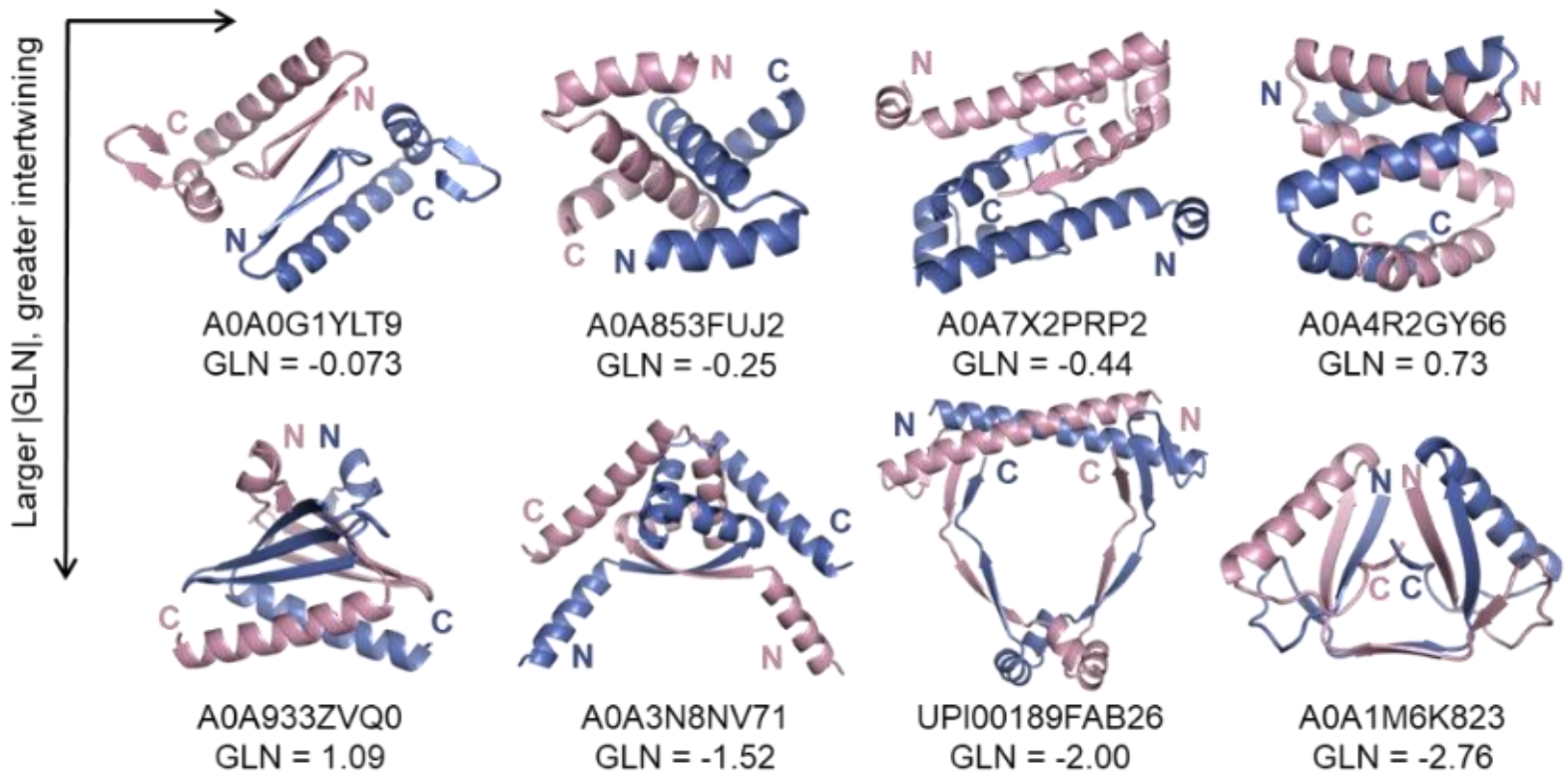

We have also added a figure in the Supplementary information (Supplementary Fig. 3, shown below) to demonstrate what |GLN| originally quantifies (the number of times two curves winding with each other) for two closed curves.

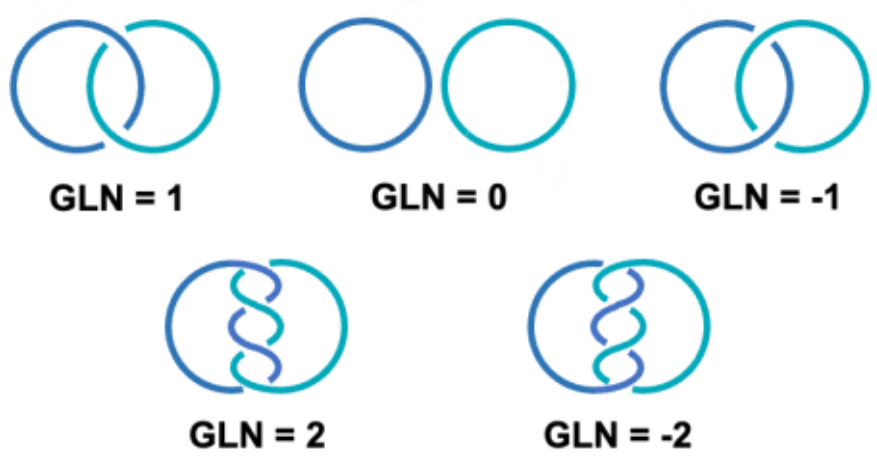

Supplementary Fig. 3. Gauss Linking Number for two closed curves. The absolute value quantifies the number of times that two closed curves wind around each other. The sign indicates the winding direction.

We also extended the discussion about GLN in the main text including its meanings, advantages over other entanglement detection techniques and the detection threshold on page 3, line 96-114, which is also shown as follows:

"To collected entangled structures, we queried an absolute Gauss Linking Number (GLN) greater than or equal to 0.7.[24,34–36] GLN is a numerical invariant originally introduced to quantify the number of times that two closed curves wind around each other in three-dimensional space (Supplementary Fig. 3). For two open curves, such as protein chains, GLN is no longer invariant but still captures the entanglement extent, with a larger absolute GLN indicating a more severe entanglement[37,38] (Figure 1f). For example, dimers with a |GLN| of 0 have their subchains stacked together without any crossings, while dimers with a |GLN| of 2 exhibit subchains that wind around each other nearly twice. Compared to other entanglement detection techniques that rely on random chain closures, the GLN of two open chains is less computationally intensive and more suitable for large-scale analysis.[27,39] A quick molecular simulation of p53dim,[40] a typical entangled homodimeric domain, demonstrates GLN's insensitivity to thermal fluctuations and small structural deviations (Supplementary Fig. 4), reinforcing its robustness as a detection criterion (see Methods and Materials for details). In this study, we established a |GLN| cutoff of 0.7 to differentiate between entangled and unentangled structures. Although this cutoff may vary depending on different definitions of entanglement or specific requirements for entanglement extent, previous results have shown that using 0.7 as a |GLN| threshold allows ESMFold to effectively identify entangled

homodimers with high precision (> 0.8)."

We constrained our screening scope to homodimers with C2 symmetry due to the fact that ESMFold exhibits poor performance in identifying entangled proteins with more subunits or higher-order symmetries aside from C2, such as C4 and D2.[4] However, this leaves a promising future research direction. We believe that the development of accurate high-throughput algorithms to discover highly complex entangled proteins would further expand the diversity of the entangled protein toolbox and facilitate the design of hydrogel networks. We added related discussion in the Discussion section on page 15, line 496-505, which is quoted below:

"As TangleDiff is a sequence generation model, it can easily be extended to generate entangled homotrimers with C3 symmetry or even higher-order structures by training with appropriate data. This work focuses solely on entangled homodimers because the accuracy of ESMFold in identifying entangled homotrimers or higher-order assemblies is currently limited, which also constrains the performance of our computational screening pipeline. Therefore, there is a need for the development of high-throughput algorithms to discover complex entangling motifs. We believe that entangled proteins with higher-order symmetries, such as C3 or D2, would significantly enhance the complexity and diversity of entanglement-crosslinked protein networks, which makes this an attractive step to address in the future"

Other comments :

Line 18: Please define stress relaxation behavior. This is key to the paper and not defined properly.

Reply: Thank you for your feedback. We have added a definition for stress relaxation behavior (page 1, line 18-19), which is also shown as follows:

"Protein hydrogels are promising artificial extracellular matrices (ECMs) for 3D stem cell and organoid culture due to their favorable stress relaxation behavior (**a decrease in stress in response to strain**)."

Line 22: Please clarify what the properties are in "control properties"

Reply: Thank you for your feedback. The term "properties" in this context refers to any protein characteristics one may care about. In this study, we specifically refer to binding energy. We have included "binding energy" as a specific example in the revised text on page 1, line 21. The updated sentence reads as follows:

"However, designing these motifs with tailored properties **such as binding energy,** remains a major challenge due to the difficulty of simultaneously controlling these properties while ensuring entanglement."

Line 24: Again, please define "entangled proteins", this is instrumental to the paper, but the

definition is not stated in the abstract

Reply: We have added the definition of "entangled proteins" in the abstract, which is also shown as follows:

"Inter-chain entangled protein motifs, **in which different protein chains are interlaced**, represent a powerful strategy to synthesize such hydrogels"

Line 28: "in-silico success rate exceeding 70%", can the authors please comment on whether this is comparable to state of the art or better?

Reply: Thank you for your feedback. To the best of our knowledge, there is no generative model developed for entangled sequence design. We have compared this with some state-of-art protein sequence generative models and added related comments on page 1, line 24-26, which is quoted as:

"TangleDiff generates diverse foldable entangled sequences with an in-silico success rate exceeding 70%, markedly outperforming current models (~1%)."

Line 37-39: This statement appears somewhat overreaching. A substantial body of prior work has already established the use of de novo design for engineering protein-based materials. We therefore suggest toning down the claim, particularly with respect to the generality of the proposed framework.

Reply: Thank you for your insightful feedback. We have narrowed down the scope of our description to entanglement design and entanglement-based biomaterials. The revised statement (page 1, line 31-32) is shown as follows.

"This work establishes a general strategy for entangled protein design, opening avenues for entanglement-based biomaterial innovation."

Line 51: Please define "entangled proteins", this is instrumental to the paper.

Reply: To be more specific and precise here, we have changed the 'entangled protein domains' here to 'inter-chain entangled protein domains' to distinguish them from self-entangling monomers. We have defined the entangled domains immediately following this sentence on page 2, line 44-48, which is also shown as follows:

"The introduction of inter-chain entangled protein domains into protein hydrogels is an effective approach to facilitate stress relaxation behaviors. The inter-chain entangled proteins are assemblies with chains physically interlaced. They provide noncovalent crosslinks in the networks, which enhance chain mobility and allow for faster force dissipation during mechanical remodeling.[14]"

Line 53: Please clarify what is p53dim?

Reply: We have clarified that p53dim refers to the "p53 dimerization domain." Both p53dim and HP0242 are homodimers consisting of two entangled chains. To provide further context, we have cited relevant references. The revised text can be found on page 2, line 49, and is quoted as follows:

"However, the existing toolbox of inter-chain entangled motifs is limited to a few natural proteins, namely the p53 dimerization domain (p53dim),[15,16] HP0242,[17,18] and their mutants,[19]"

Line 58: What are knots and links in protein topologies? This definition is important to to understanding the manuscript.

Reply: We have added brief definitions of knots and links in the corresponding section. Additionally, we have cited references that provide rigorous mathematical definitions and their extensions to proteins. The revised text can be found on page 2, line 55-56, and is quoted as follows:

"Still, the specific challenge of designing entangled topologies, such as knots (monomers with self-entangling backbones)[24,25] or links (two chains interlinked through entanglements)[26,27] from scratch remains particularly difficult"

Line 62-64: The meaning of this sentence is unclear. The authors should clarify what is meant by "varying chain-intertwining across an expansive design space" and how de novo designed entangled domains are being applied in the context of viscoelasticity.

Rely: When we refer to "varying chain-intertwining across an expansive design space," we mean designing entangled dimers with diverse sequential and structural characteristics that encompass a wide range of possibilities. The de novo designed entangled domains are incorporated into the hydrogel networks as physical crosslinking points. We have revised our statement on page 2, line 60-62, which is shown as follows.

"Systematically varying sequential and structural characteristics of entangled dimers across an expansive design space and applying de novo designed entangled domains as physical crosslinks to viscoelastic protein networks remains central challenges in the field."

Line 68: Please define "chain intertwining."

Reply: We have added the definition on page 2, line 66-67, and is quoted as follows:

"Our approach began with mining sequence databases to identify features conducive to chain intertwining (two chains entangled with each other)."

Line 95: Could the authors please clarify what is meant by an "entanglement judge"?

Rely: By stating that we "cropped their terminal coil regions whose conformational uncertainty may affect entanglement judge," we mean that the random coil regions in the predicted structures may influence the determination of entanglement. The coil terminals of the two chains predicted by ESMFold may be sufficiently long to intertwine, leading to entanglements that, despite high pLDDT scores, are formed through coil structures that lack robustness due to their inherent flexibility. Therefore, we cropped them before computing the GLN to ensure that the entanglement was not formed by the terminal coils in our training set.

We have revised this statement for clarity (page 3, line 93-96), which is quoted as follows:

"We predicted homodimeric structures using ESMFold[34] and cropped their terminal coil regions to ensure that the subsequent entanglement detection step excludes structures where entanglement is formed by these terminal regions. The entanglements formed by the terminal coils are not robust due to their inherent flexibility (Supplementary Fig. 2)."

Figure 1: In panel c, could the authors please clarify the origin of the number 12,742,356? Is this value derived from the initial filtering stage? Please also see the earlier major comment regarding the GLN metric—showing the GLN distribution in the main figure would help readers understand the basis for the |GLN| > 0.7 threshold. In addition, in panel g, the term "cellular organisms" is unclear and should be clarified.

Reply:

The updated Figure 1 is shown as follows for your reference.

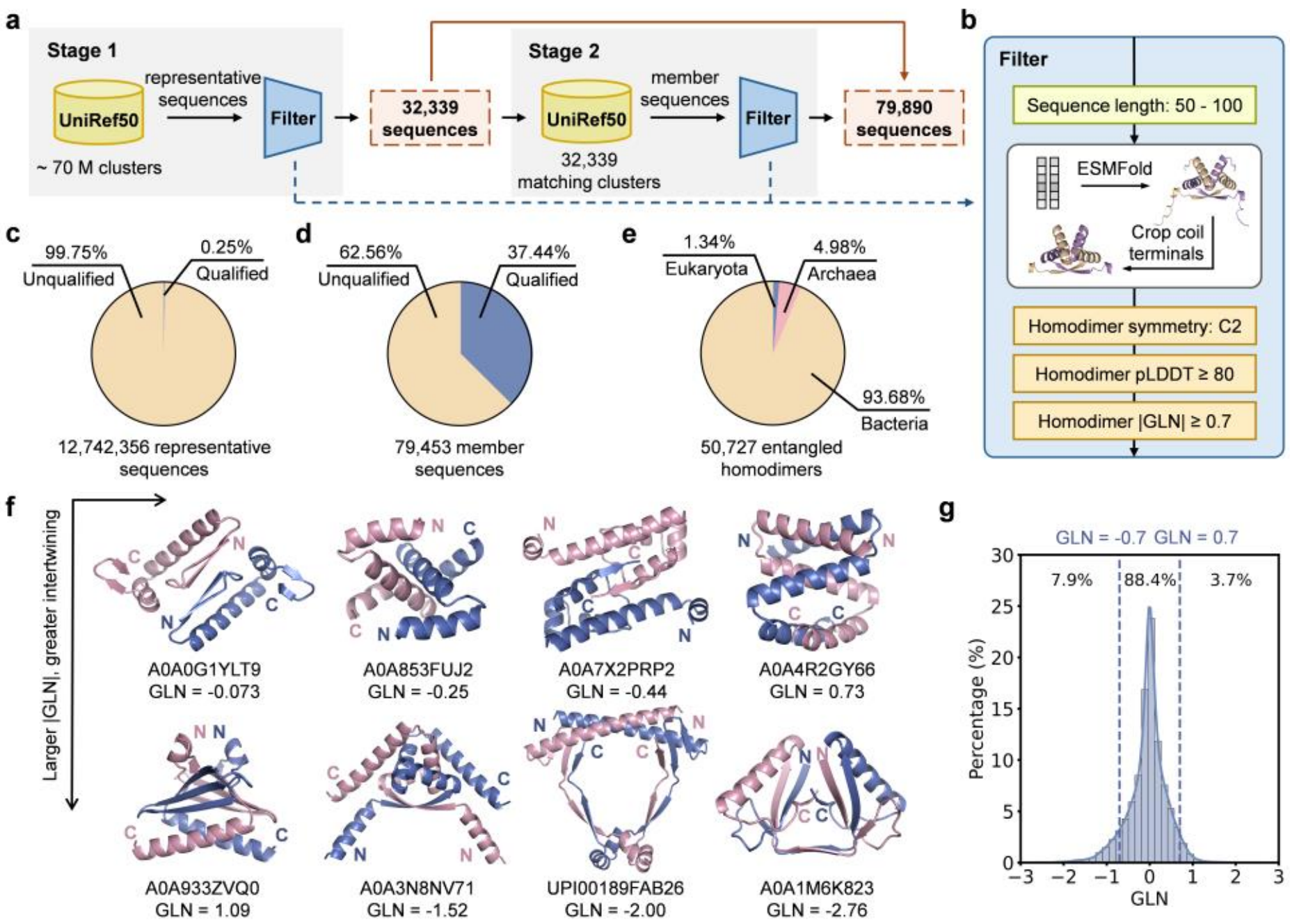

In panel c, 12,742,356 represents the total number of representative sequences rather than three

separated numbers.

We have added panel g, the GLN distribution to help understand the basis for the |GLN| > 0.7 threshold.

We have moved the original panel g to Supplementary Fig. 5 as suggested by another reviewer. And we have clarified the "cellular organisms" in the caption, which is also quoted as follows:

"Some sequences are not annotated with a specific organism in the UniProt database but are categorized broadly as "cellular organisms" (living entities composed of one or more cells, which are distinguished from non-cellular entities such as viruses)."

Figure 2: In panel e, the y-axis label is missing.

Reply: Thank you for your feedback. The y axis is a dimensionless number between 0 and 1. The legend explains what each curve represents.

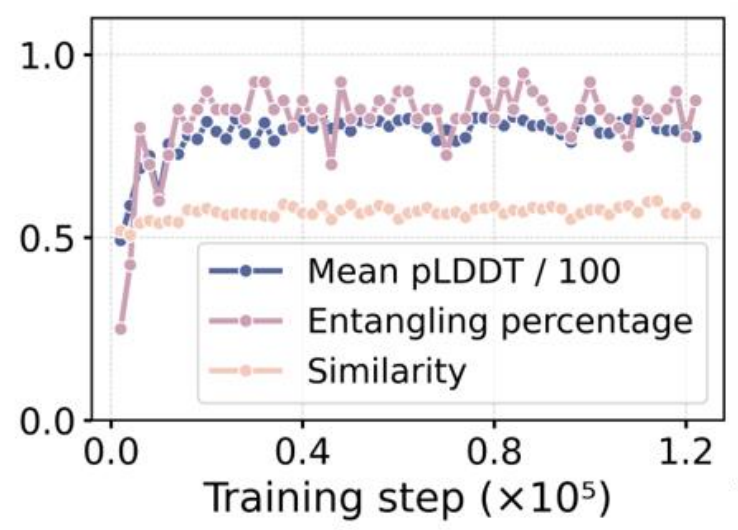

Line 175: It would be helpful if the authors could place the reported ~70% success rate in the context of existing approaches and clarify whether this performance represents state-of-the-art or better results.

Reply: Thank you for your feedback. As mentioned in the previous comment, to our best knowledge, there is no generative model developed for entangled sequence design. But we have compared our approach with current state-of-the-art protein sequence generative models. Related results are shown in Supplementary Fig. 8:

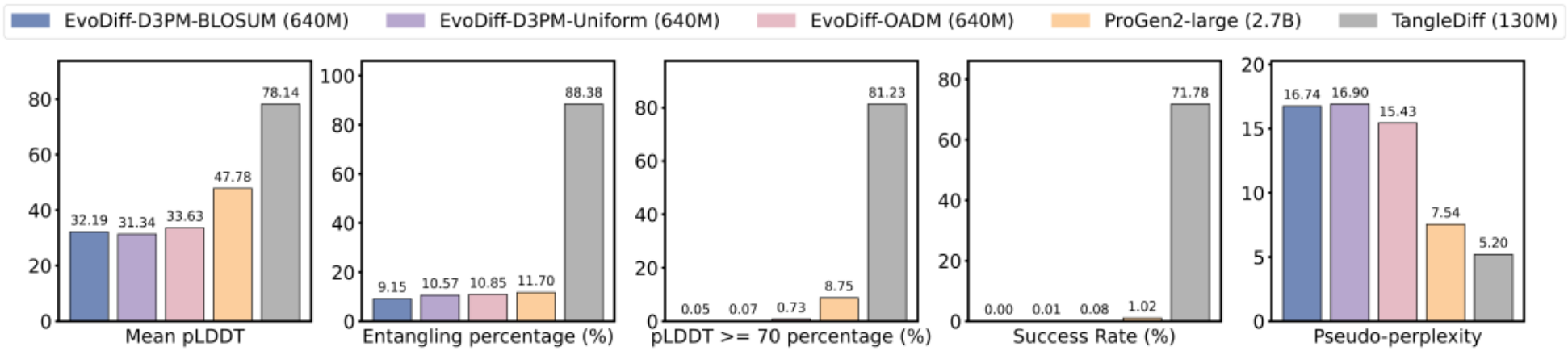

Supplementary Fig. 8. Generation performance of TangleDiff compared to other sequence generation models, including EvoDiff[7] and ProGen2[8]. TangleDiff outperformed the other models in both sequence quality and entangling percentage, achieving a success rate of 71.78% (percentage of designs that are both entangled and have a pLDDT > 70), significantly higher than the next best model, which achieved only 1.02%. All structures were predicted using ESMFold. Pseudo-perplexity is calculated by averaging the prediction probabilities provided by ESM2-650M across all positions in a sequence. This metric estimates how well the masked language model (in this case, ESM2-650M) comprehends the sequence. A lower pseudo-perplexity suggests that the sequence appears more natural and predictable to the model, while a higher pseudo-perplexity indicates that the sequence is unusual or unexpected.

Discussions have been added to the main text on page 6, line 196-198, which is also shown as follows.

"Consequently, TangleDiff demonstrates an overall in silico design success rate exceeding 70% (Figure 3c), significantly surpassing that of current sequence generation models, with the best alternative achieving only 1.02% (Supplementary Fig. 8)."

Figure 3: In panel b, could the authors include the number of sequences in each bin of the box plot? For example, how many of the 4,000 designs fall into the pLDDT > 80 bin? In panels d and e, it would be helpful to report the corresponding |GLN| metric for each structural example shown. For the UMAP analysis, please clarify whether it was performed in a one-hot–encoded sequence space or using an LLM-based embedding. Additionally, the UMAP appears to contain fewer than 4,000 points; the authors should comment on this discrepancy. Finally, the last example shown in panel e appears to be a highly "unnatural" dimer—could the authors clarify whether this corresponds to the c0_2898 design

Reply:

For panel b (now panel d in the revised version), we have updated the box plots to display sequence identity distribution. We have annotated the number of sequences that fall into each pLDDT range next to each distribution plot for better clarity.

In panels d and e (now panels f and g in the revised version), we have added the corresponding GLN values for each structural example.

Regarding the UMAP analysis, we have verified that the plot contains 4,000 points. It may appear fewer as many of them are overlapped. We have clarified in the figure caption that the UMAP analysis was conducted using LLM-based embeddings, as stated:

"Uniform Manifold Approximation and Projection (UMAP) of the protein-level ESM2-650M sequence embeddings of both natural and designed entangled homodimers."

Lastly, the example shown in panel e is not the C0_2898 design. This example was generated

through unconditional generation, whereas C0_2898 was produced under the condition of binding energy < -160 REU. However, they do share similar structures.

The updated Figure 3 is shown as follows for your reference.

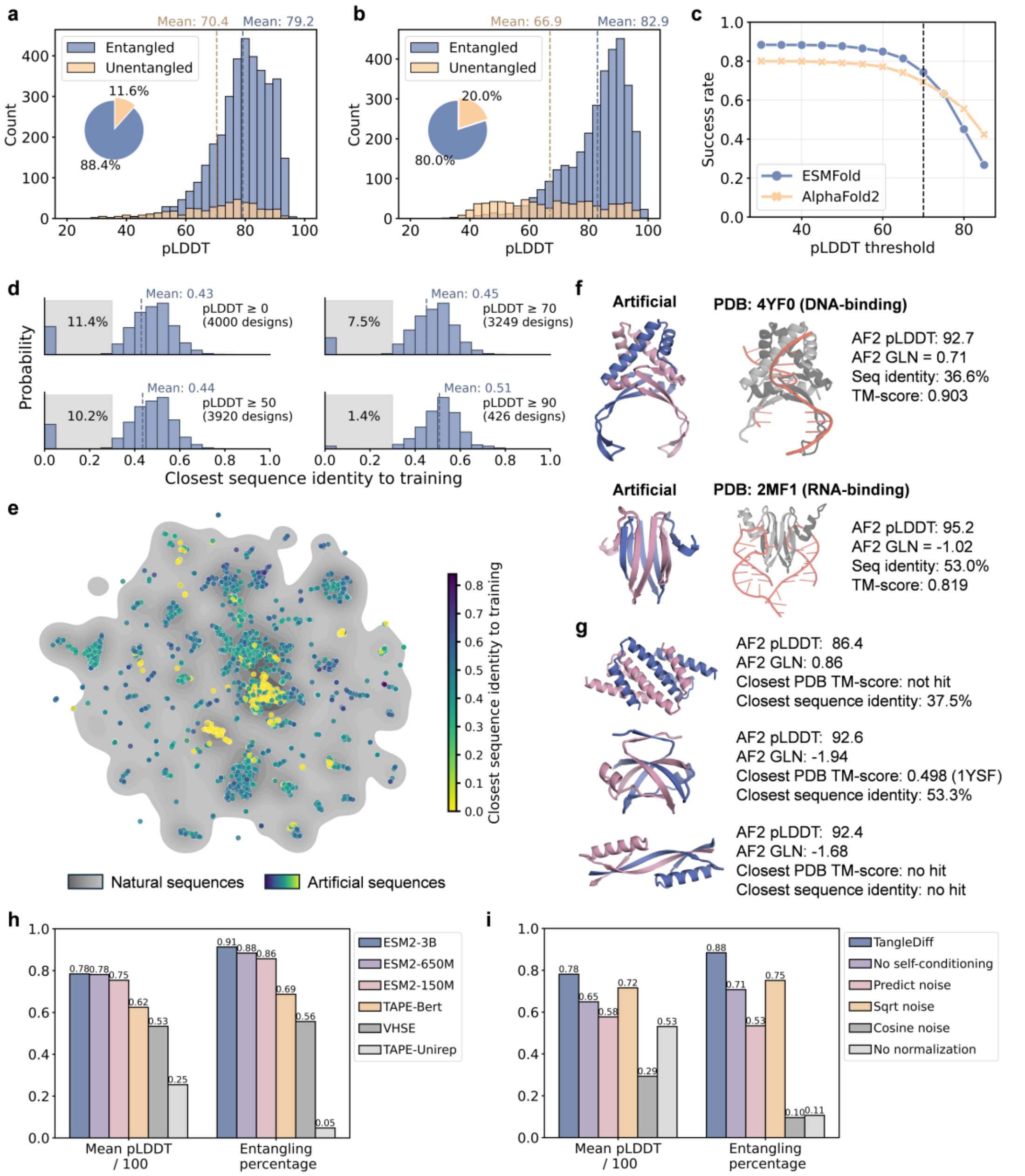

Figure 4: In panel a, what does "Control" refer to? In panel b, could the authors provide the number of sequences in each bin? For consistency, it would be helpful to do the same elsewhere where applicable. In panel e, increasing the visibility of the symmetric unit would improve clarity, as it is currently difficult to distinguish the individual monomers. Additionally, it is interesting that only the smaller (blue) domains lack close sequence identity matches; could the authors comment on this observation and its possible implications?

Reply:

Updated Figure 4 is shown as follows for your reference.

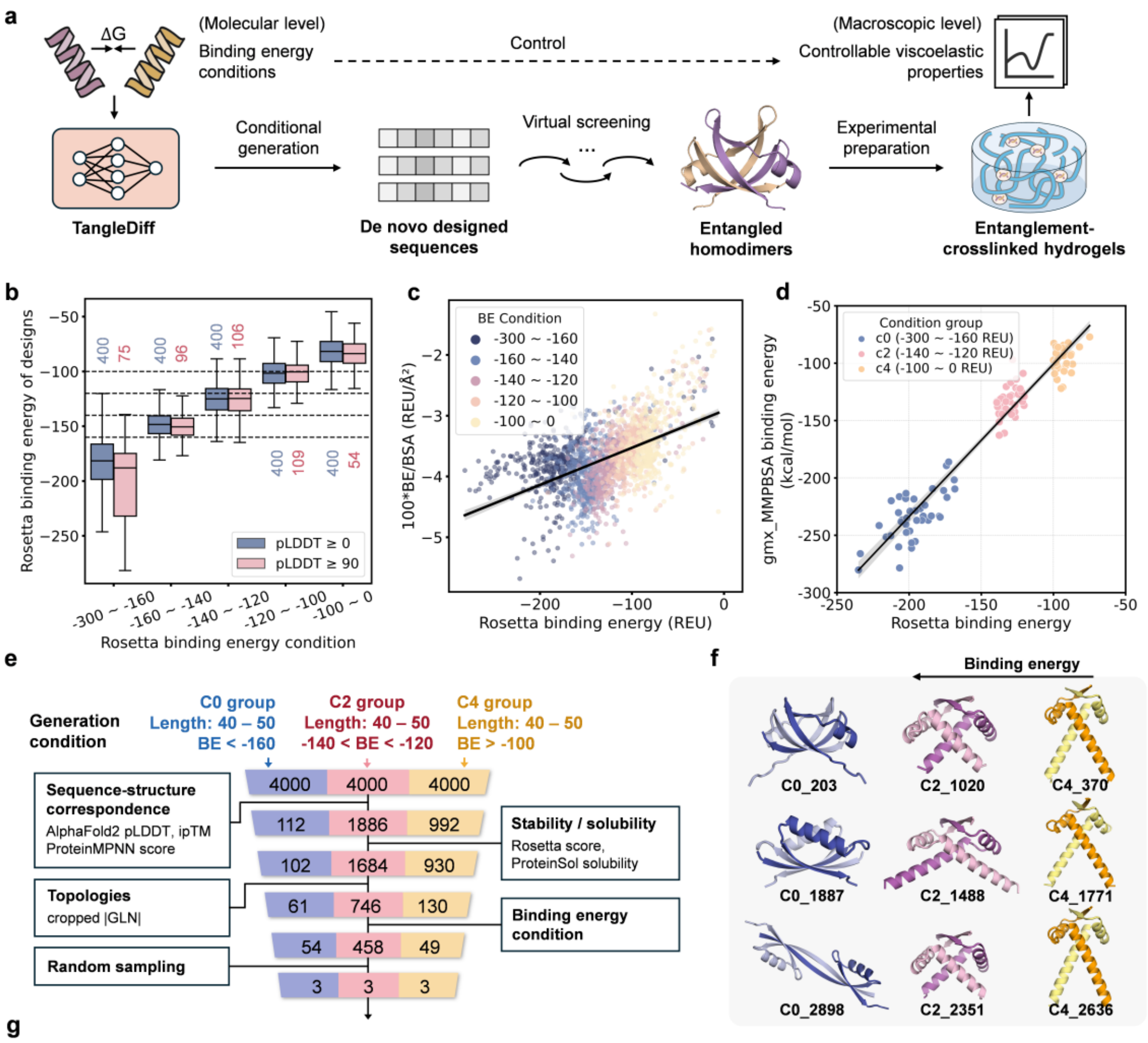

g

Design ID	C0_2898	C0_203	C0_1887	C2_1020	C2_2351	C2_1488	C4_1771	C4_2636	C4_370
Closest sequence identity	No hit	No hit	No hit	37.5%	62.9%	56.8%	41.7%	40.4%	43.2%

In panel a, "Control" refers to the ability to manipulate the viscoelastic properties of the prepared hydrogels based on the binding energy conditions specified during generation. This allows for the synthesis of hydrogels with varying viscoelastic properties. We have clarified this in the caption:

"Schematic of **controlling** rheological behaviors of entanglement-crosslinked hydrogels by conditioning the generation of TangleDiff on binding energies."

In panel b, we have annotated the number of sequences in each bin. As previously mentioned, the other boxplot (Figure 3d) has been replaced by distribution plots. We have annotated the number of sequences that fall into each pLDDT range beside each distribution plot.

For panel e (now panel f in the revised version), we have optimized the color scheme to enhance the visibility of different symmetric units, making it easier to distinguish between individual

chains. Regarding the observation that only the smaller (blue) domains (designs with stronger binding energies) lack close sequence identity matches, we hypothesize that this may be attributed to the inherent structural diversity and variability of beta sheets compared to alpha helices as this group formed entangled structures through twisted beta sheets. The structural diversity of beta sheets, which includes variations such as parallel or antiparallel orientations and differing degrees of twist, likely contributes to the higher novelty observed. We discuss this phenomenon in the main text on page 11, line 340-343, which is quoted as follows:

"All designs with strong binding energies (< -160 REU) lack similar sequence matches in the training set, demonstrating the highest novelty among three groups. This observation may be attributed to the greater structural diversity and variability of beta sheets compared to alpha helices.[58]"

Line 276-292 and Figure 4: It is well known that Rosetta energy scales with sequence length; therefore, a linear correlation between length and energy units is not particularly informative. A more informative metric would be one that is normalized by length. The authors should consider reporting or switching to a length-normalized energy metric.

Reply: Thank you for your valuable comments. In response to your suggestion, we have reported another metric, 100 times binding energy over the buried surface area (100*BE/BSA, REU/Å^2) to demonstrate the binding energy per interface area. We have reported the correlation between the binding energy, the binding energy per interface area and the binding energy condition in Figure 4c (shown above).

The binding energy conditions specified during generation not only correlate with the Rosetta binding energy, but also the binding energy per interface area. We have added related discussions in the main text on page 10, line 313-314, which is shown as follows.

"Although not explicitly specified during generation, the binding energy per area of generated designs exhibited a trend consistent with the overall binding energy (Figure 4c)."

In this study, we conditioned TangleDiff on different binding energy groups rather than on binding energy per unit area. We suppose that binding energy, an overall reflection of BSA, extent of entanglement, and binding quality, is more closely related to the relaxation behaviors of hydrogel networks.

Figure 5: In panel a, the main differences between the two modalities used for the different constructs are not clear, particularly for non-expert readers. How are these modalities expected to differ in their biophysical properties, and why is it important to test both? In panel b, it would be helpful to report the molecular weight (MW) of each design. In panel c, the color scheme makes it difficult to identify which profile corresponds to which design; stratifying the designs based on secondary structure could improve clarity. In addition, the interpretation of the different profiles is not immediately obvious, and a schematic illustrating the different modalities would aid understanding. Moreover, the designed domains are sufficiently long to

allow quantitative analysis of their secondary-structure content (helix, β-sheet, and coil). Such an analysis would provide critical insight into whether the designs are indeed folding into their intended structures. In panel e, there is no negative control, making it unclear how challenging it is to form hydrogels using the SpyTag/SpyCatcher constructs alone. Including a natural protein as a reference would strengthen the analysis. This comment also applies to Figure 6. Panel d is very nice!

Reply:

Updated Figure 5 is shown as follows for your reference.

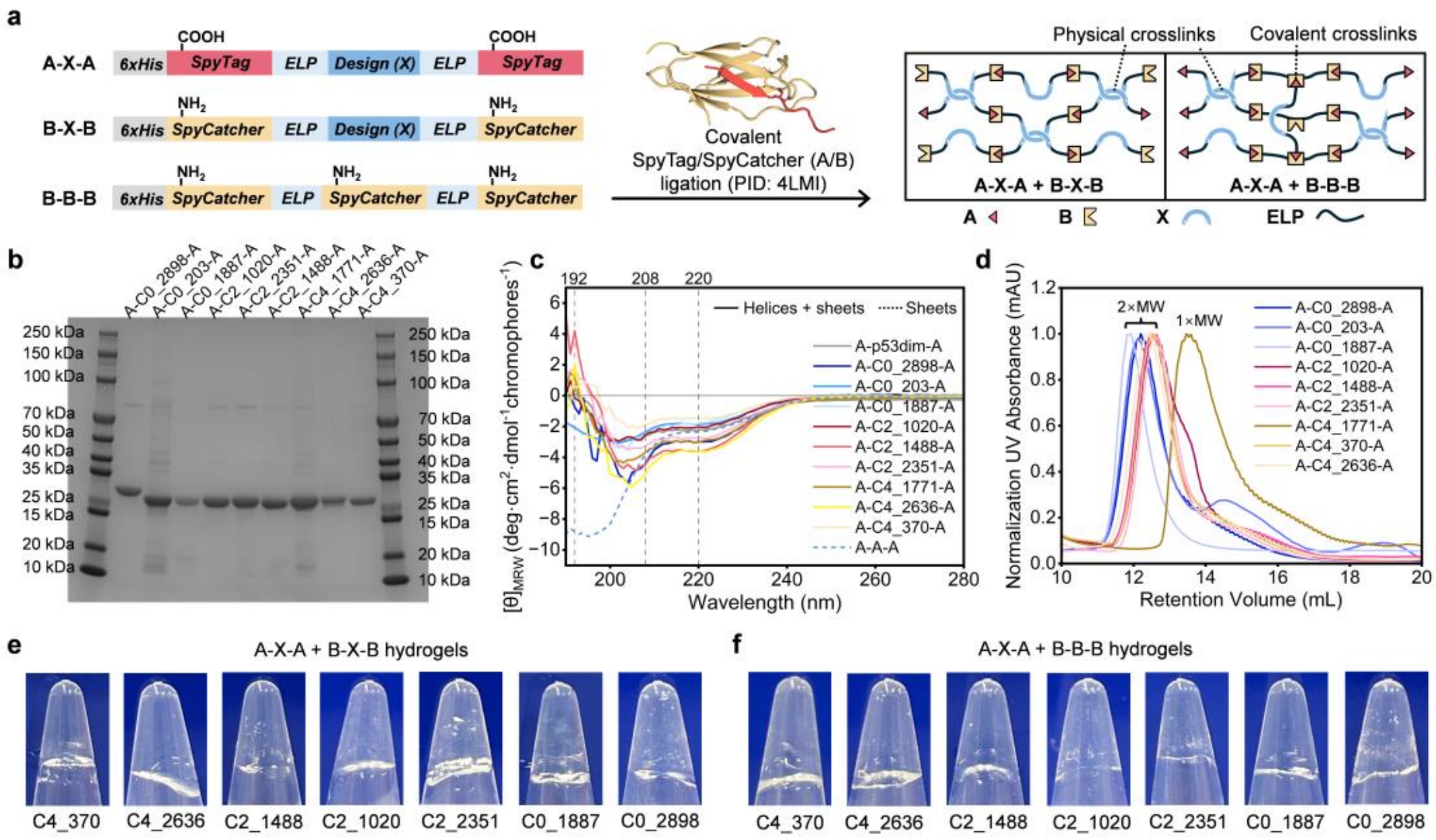

Panel a: The A-X-A + B-X-B networks contain only crosslinks formed by physical entanglements, whereas the A-X-A + B-B-B networks incorporate both physical and covalent crosslinks. As a result, the A-X-A + B-B-B network is expected to exhibit higher mechanical strength (e.g., a higher storage modulus, G'). We have annotated positions of covalent and physical crosslinks in the schematic.

We tested both because comparing these two helps highlight the contribution of physical crosslinks and covalent crosslinks respectively. We have also added related justification on page 12, line 383-388, and page 14, line 422-426, which is quoted as follows:

"The A-X-A + B-X-B networks consist solely of crosslinks formed by physical entanglements, whereas the A-X-A + B-B-B networks contain both physical and covalent crosslinks. Although the A-X-A + B-B-B networks demonstrate greater applicability for cell incubation due to their higher mechanical strength, we also prepared the A-X-A + B-X-B networks, which are entirely physically crosslinked, to highlight the contribution of chain entanglements."

"In contrast, the A-X-A + B-B-B networks exhibited relaxation peaks at the same positions but with higher peak heights, demonstrating that the introduction of covalent crosslinks increases the relaxation strength without affecting the relaxation time or introducing new relaxation

behaviors."

Panel b: We have reported the molecular weight of each design in Supplementary Fig. 1, beside the corresponding sequence information.

Panel c: We utilized two algorithms, SELCON[7] and SESCA[8], to quantitatively analyze the secondary structure composition via the ChiraKit website.[9] The results are shown below. We found that these algorithms provide significantly different results, making it challenging to obtain accurate quantitative measurements. Various factors contribute to this issue, including the structural diversity of β-sheets, which can exhibit significant differences in their CD spectra based on subtle variations in orientation (parallel vs. antiparallel) and twist. Additionally, secondary structure analysis algorithms are highly sensitive to protein concentration and cuvette path length; small deviations can lead to substantial differences. Background noise may also complicate the analysis.

Sample	SELCON2 (%)					SESCA (%)			
	Alpha	Beta	Turns	Unord	Total	Alpha	Beta	Coil	Total
A-C4_370-A	6.9	45.1	10.4	37.7	100.2	1.53	50.89	47.58	100
A-C4_1771-A	16.2	27.4	11.7	43.4	98.7	4.1	37.65	58.25	100
A-C4_2636-A	23.5	27.5	13.5	32.2	96.6	6.34	32.06	61.6	100
A-C2_1488-A	20.4	38.4	11.4	29.4	99.5	7.88	36.19	55.93	100
A-C2_1020-A	9.1	24.3	20.5	48	102	2.77	44.42	52.81	100
A-C2_2351-A	-1.4	34.9	20.1	45.1	98.7	3.24	40.75	56.01	100
A-C0_203-A	15.1	23.3	17	44.2	99.6	3.38	41.8	54.82	100
A-C0_2898-A	10.9	30.8	14.7	41.6	98	4.63	35.07	60.3	100
A-C0_1887-A	-1.8	33.1	24.4	44.8	100.5	2.51	44.13	53.36	100
A-A-A	11.6	30	15.2	44.4	101.2	2.03	25.72	72.25	100

Qualitatively, we assessed a negative control group, A-A-A, which consists solely of beta sheets and coils, alongside a positive control, A-p53dim-A, which includes alpha helices, beta sheets, and coils for comparison. All of our designs resemble A-p53dim-A, indicating a mixture of alpha helices and beta sheets. To improve clarity, we have differentiated between helices and the combination of helices and sheets by using dashed and solid lines, respectively. The panel has been updated accordingly.

Panel e: We have included a negative control in our revised manuscript, consisting of A-X-A + B-X-B and A-X-X + B-B-B protein networks prepared from the design C4_1771 under the same conditions. C4_1771 has a similar length to the other designs but has been demonstrated

to have no dimeric form, as confirmed by SEC-MALS results shown in Figure 5d, indicating that it is not entangled. The results are demonstrated in Supplementary Fig. 18, which is also shown as follows.

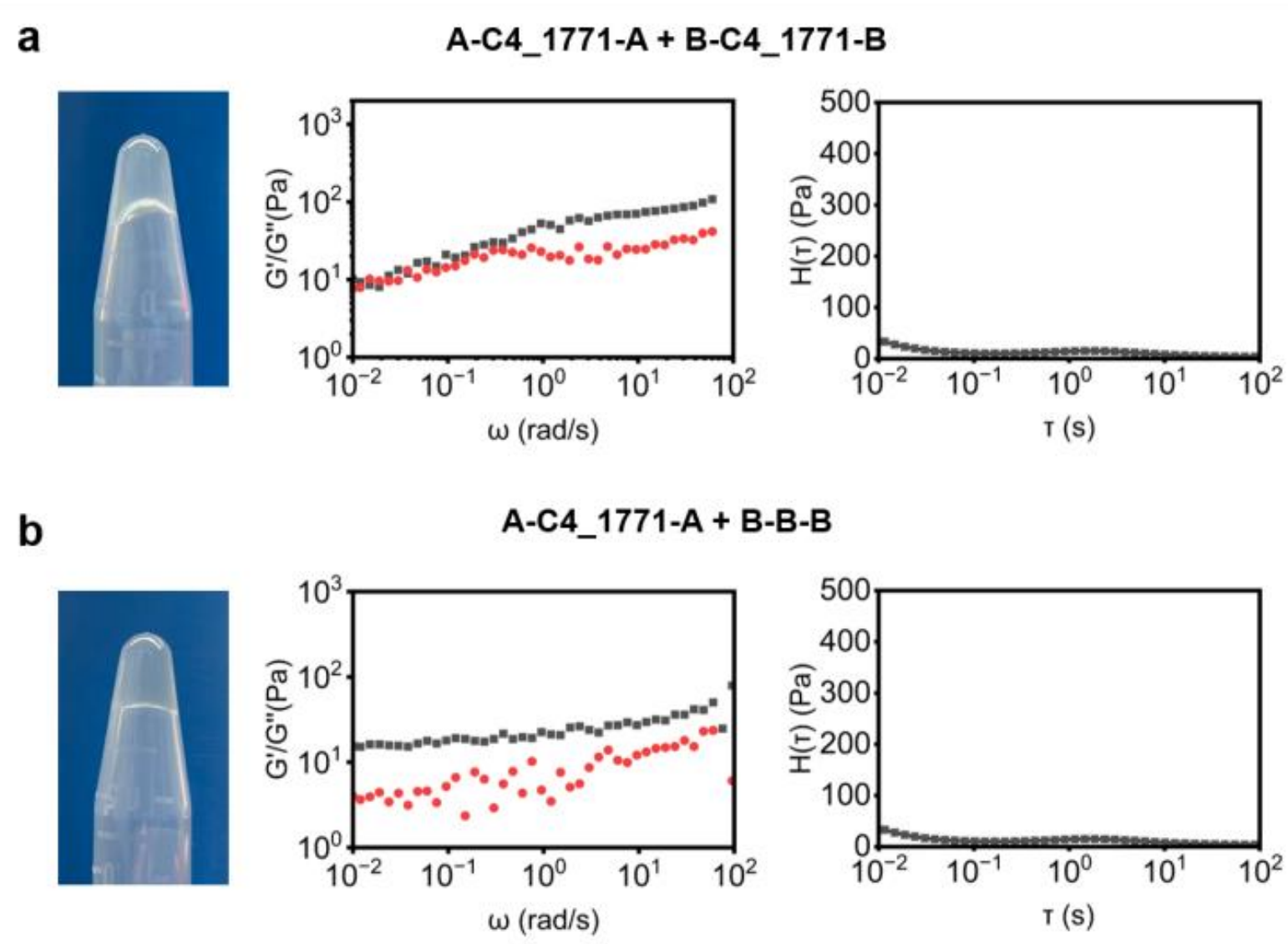

Supplementary Fig. 18. Protein networks with design C4_1771 of the (a) A-X-A + B-X-B type and (b) A-X-A + B-B-B type. Left: a photo of solution mixture. Middle: dynamic mechanical analysis. Right: continuous relaxation spectra.

Figure 6: For non-expert readers, these plots are difficult to interpret. The authors should consider including a schematic illustrating what G' and G" represent, as well as the meaning of H(τ). In particular, what does the magnitude of the peak in H(τ) signify physically? It appears that most designs exhibit relatively flat H(τ) profiles, and stratification by predicted binding energy does not substantially clarify the observed differences. Are these profiles consistent with the authors' expectations, and if so, how? Binding energy alone seems unlikely to explain the variance in the data; the authors may wish to consider and discuss alternative or additional metrics, such as interface surface area, interface chemistry, or other structural features. Please also see the earlier comment regarding the inclusion of negative controls and natural protein controls.

Reply:

Thanks for your constructive suggestions. Updated Figure 5 is shown as follows for your reference.

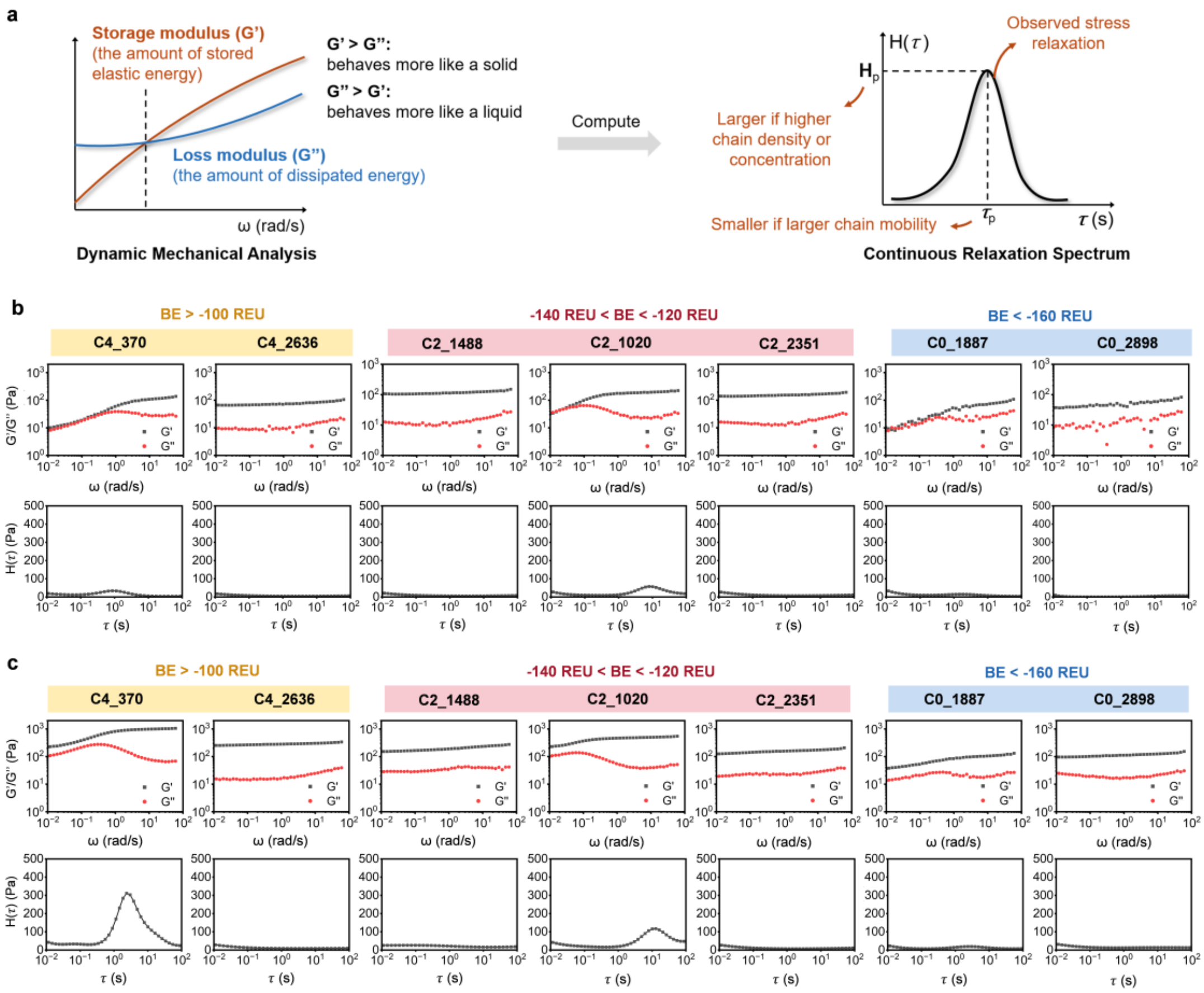

We have added panel a, a schematic to illustrate the meaning of G′, G″ and peaks in the H(τ) spectrum. Specifically, G' measures the amount of stored elastic energy and G″ measures the amount of dissipated energy. The relative amount of G' and G'' indicates whether the materials exhibit predominantly solid-like or fluid-like behavior. Peaks in the continuous relaxation spectra reflect the observed relaxation behavior, with their positions (relaxation times) determined by the chain mobility (the ability of chains or chain segments to undergo conformational rearrangements, translation, or reorientation over time) and their heights determined by the chain density. We expect that the inter-chain binding energy will affect the mobility between chains and then influence the peak positions in H(τ) spectra, with smaller peak positions indicating weaker binding energy. Related descriptions have been added in the main text on page 12, line 388-398, which is shown as follows.

"Dynamic mechanical analysis was performed to assess the rheological behaviors of the prepared networks by measuring the storage (G') and loss modulus (G''). This assessment indicates whether the materials exhibit predominantly solid-like or fluid-like behavior (Figure 6a). To quantify the full distribution of relaxation times and correlate the macroscopic stress-relaxing behavior with binding energies at the molecular level, we converted the frequency sweep data (G' and G'') into a series of continuous relaxation spectra $H(\tau)$ (Methods). Peaks in the continuous relaxation spectra reflect the relaxation behavior, with their positions (relaxation times) determined by the chain mobility (the ability of chains or chain segments to undergo conformational rearrangements, translation, or reorientation over time), which we

hypothesize is dominantly influenced by inter-chain binding energy (Figure 6a)."

The results of C4_370, C2_1020, C0_1887 and C0_2898 meet our expectations:

C4_370: weakest binding energy -> smallest peak position

C2_1020: medium binding energy -> larger peak position compared with C4

C0_1887, C0_2898: strongest binding energy -> no peaks, reflecting the relaxation time much higher

The results of C4_2636, C2_1488, C2_2351 do not meet our expectations. We have discussed possible reasons in the main text on page 14, line 426-430, which is shown as follows.

"Additionally, relaxation peaks were not observed in hydrogels prepared from designs C4_2636, C2_1488, and C2_2351. We propose that these unexpected findings may be attributed to various factors, such as differing structural features (e.g., buried surface area, secondary structure content, terminal distances) or additional intermolecular interactions (for example, dimeric designs may interact to form higher-order assemblies)."

We have also added the mechanical curves of two negative control networks (A-C4_1771-A + B-B-B and A-C4_1771-A + B-C4_1771-B) in Supplementary Fig. 18 (shown below) as a negative control. These samples did not exhibit continuous modulus curves, even for the A-C4_1771-A + B-B-B network with covalent crosslinks, and their storage modulus (G') was significantly lower than that of the other designs. The relaxation spectra H(τ) also showed no evidence of stress relaxation behavior.

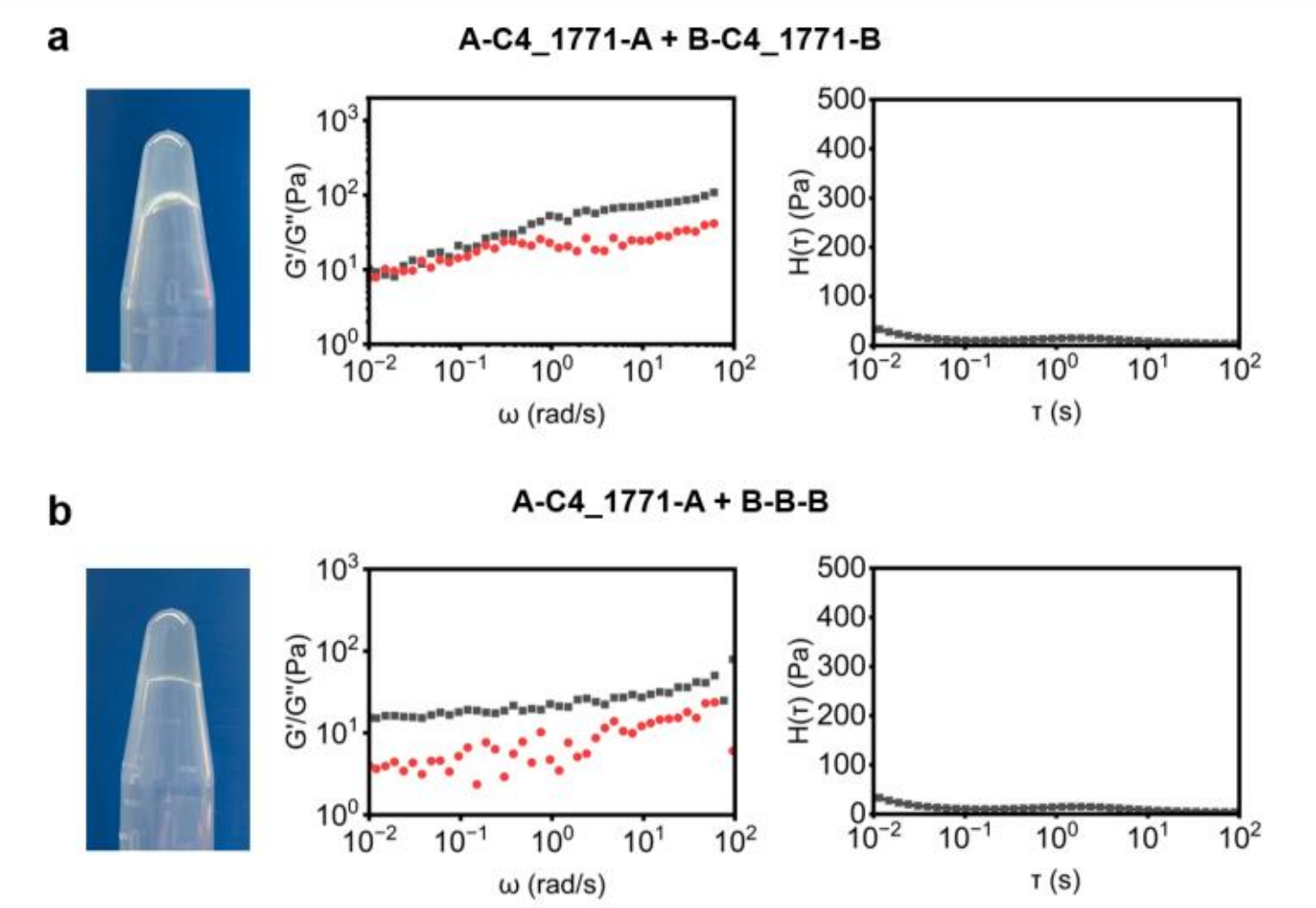

Supplementary Fig. 18. Protein networks with design C4_1771 of the (a) A-X-A + B-X-B type and (b) A-X-A + B-B-B type. Left: a photo of solution mixture. Middle: dynamic mechanical analysis. Right: continuous relaxation spectra.

We have added related discussions in the main text on page 14, line 431-440, which is also shown as follows.

"As a negative control, we prepared A-X-A+B-X-B and A-X-A+B-B-B hydrogels from the design C4_1771 that has a similar length to the other designs but does not exhibit dimerization

or entanglement (as indicated by the SEC-MALS results). Unlike the other designs that formed stable gels, networks made from C4_1771 of both two types remained viscous liquids that gradually flowed down the walls of the container (Supplementary Fig. 18). Their G' was significantly lower than that of the other designs and the relaxation spectra also showed no evidence of stress relaxation behavior. This proves that extra physical crosslinks were necessary for the gel formation and that the relaxation behavior observed in the hydrogels originated from physical entanglements within the networks, rather than from other compositions such as covalent crosslinks or ELP chain dynamics."

Line 379: The manuscript refers to the designs as "controllable"; please clarify what parameters are controllable and how this control is achieved.

Reply: The binding energies of designed entangled proteins are controllable. This is achieved by the adaLN-Zero module which is discussed in the model development section.

We have revised this statement for better clarity on page 15, line 473-476, which is also shown as follows:

"Our method advances the field of entanglement-crosslinked protein hydrogels by facilitating the efficient design of foldable, novel, and diverse entangled proteins with controllable binding energy through TangleDiff, a transformer-based diffusion model integrated with the adaLN-Zero module."

Methods:

Line 391: What does 50-100 refer to? Do the authors mean amino acids?

Reply: It refers to amino acids. We have added "a.a." for clarification on page 16, line 509-510, which is quoted as:

"We queried all representative sequences from UniRef50 with a length ranging from 50 a.a. to 100 a.a."

Supplementary Information

Line 57: The authors should add a color legend for readability, as the meaning of the yellow, red, blue, and green colors is not defined in the figure or the figure legend.

Reply: Thanks for the suggestion. We have added a legend annotating the meaning of different colors in Supplementary Fig. 1, which is also shown below:

Supplementary Figures

green: The polyhistidine tag used for the affinity purification of proteins
red: SpyTag
orange: SpyCatcher
blue: Designed sequences
grey: TEV protease site

(1) B-B-B (MW: 54.9 kDa)

```
001 MKGSSHHHHHHVDIPTTENLYFQGAMVDTLSGLSSEQGQSGDMTIEEDSA
051 THIKFSKRDEDGKELAGATMELRDSSGKTISTWISDGQVKDFYLYPGKYT
101 FVETAAPDGYEVATAITFTVNEQGQVTVNGKATKGDAHIDGPQGIWGQLD
151 GHGVGVPGVGVPGVGVPGEGVPGVGVPGVGVPGVGVPGVGVPGEGVPGVG
201 VPGVGVPGVGVPGVGVPGEGVPGVGVPGVGELGRGDDSATHIKFSKRDED
251 GKELAGATMELRDSSGKTISTWISDGQVKDFYLYPGKYTFVETAAPDGYE
301 VATAITFTVNEQGQVTVNGTSVPGVGVPGVGVPGEGVPGVGVPGVGVPGV
351 GVPGVGVPGEGVPGVGVPGVGVPGVGVPGVGVPGEGVPGVGVPGVGVPGG
401 LLDIPTTENLYFQGAMVDTLSGLSSEQGQSGDMTIEEDSATHIKFSKRDE
```

References

(1) Liu, Y.; Duan, Z.; Fang, J.; Zhang, F.; Xiao, J.; Zhang, W. Cellular Synthesis and X-ray Crystal Structure of a Designed Protein Heterocatenane. *Angew. Chem. Int. Ed.* **2020**, *59* (37), 16122–16127. https://doi.org/10.1002/anie.202005490.

(2) Wu, W.-H.; Bai, X.; Shao, Y.; Yang, C.; Wei, J.; Wei, W.; Zhang, W.-B. Higher Order Protein Catenation Leads to an Artificial Antibody with Enhanced Affinity and In Vivo Stability. *J. Am. Chem. Soc.* **2021**, *143* (43), 18029–18040. https://doi.org/10.1021/jacs.1c06169.

(3) Yang, T.; Da, X.-D.; Zhang, W.-B. Design and Biosynthesis of Topological Proteins. *Acta Polym. Sin.* **2021**, *51* (8), 804–816. https://doi.org/10.11777/j.issn1000-3304.2020.20065.

(4) Deng, P.; Zhang, Y.; Xu, L.; Lyu, J.; Li, L.; Sun, F.; Zhang, W.-B.; Gao, H. Computational Discovery and Systematic Analysis of Protein Entangling Motifs in Nature: From Algorithm to Database. *Chem. Sci.* **2025**, *16* (20), 8998–9009. https://doi.org/10.1039/D4SC08649J.

(5) Jumper, J.; Evans, R.; Pritzel, A.; Green, T.; Figurnov, M.; Ronneberger, O.; Tunyasuvunakool, K.; Bates, R.; Žídek, A.; Potapenko, A.; Bridgland, A.; Meyer, C.; Kohl, S. A. A.; Ballard, A. J.; Cowie, A.; Romera-Paredes, B.; Nikolov, S.; Jain, R.; Adler, J.; Back, T.; Petersen, S.; Reiman, D.; Clancy, E.; Zielinski, M.; Steinegger, M.; Pacholska, M.; Berghammer, T.; Bodenstein, S.; Silver, D.; Vinyals, O.; Senior, A. W.; Kavukcuoglu, K.; Kohli, P.; Hassabis, D. Highly Accurate Protein Structure Prediction with AlphaFold. *Nature* **2021**, *596* (7873), 583–589. https://doi.org/10.1038/s41586-021-03819-2.

(6) Lin, Z.; Akin, H.; Rao, R.; Hie, B.; Zhu, Z.; Lu, W.; Smetanin, N.; Verkuil, R.; Kabeli, O.; Shmueli, Y.; dos Santos Costa, A.; Fazel-Zarandi, M.; Sercu, T.; Candido, S.; Rives, A. Evolutionary-Scale Prediction of Atomic-Level Protein Structure with a Language Model. *Science* **2023**, *379* (6637), 1123–1130. https://doi.org/10.1126/science.ade2574.

(7) Sreerama, N.; Venyaminov, S. Yu.; Woody, R. W. Estimation of Protein Secondary Structure from Circular Dichroism Spectra: Inclusion of Denatured Proteins with Native Proteins in the Analysis. *Anal. Biochem.* **2000**, *287* (2), 243–251. https://doi.org/10.1006/abio.2000.4879.

(8) Nagy, G.; Grubmuller, H. Implementation of a Bayesian Secondary Structure Estimation Method for the SESCA Circular Dichroism Analysis Package. *Comput. Phys. Commun.* **2021**, *266*, 108022. https://doi.org/10.1016/j.cpc.2021.108022.

(9) Burastero, O.; Jones, N. C.; Defelipe, L. A.; Zavrtanik, U.; Hadži, S.; Hoffmann, S. V.; Garcia-Alai, M. M. ChiraKit: An Online Tool for the Analysis of Circular Dichroism Spectroscopy Data. *Nucleic Acids Res.* **2025**, *53* (W1), W158–W168. https://doi.org/10.1093/nar/gkaf350.
